# Biosynthesis of mushroom-derived type II ganoderic acids by engineered yeast

**DOI:** 10.1038/s41467-022-35500-1

**Published:** 2022-12-14

**Authors:** Wei Yuan, Chenjian Jiang, Qin Wang, Yubo Fang, Jin Wang, Meng Wang, Han Xiao

**Affiliations:** 1 University of Chinese Academy of Sciences, Beijing, 100049 China; 2 Key Laboratory of Systems Microbial Biotechnology, Tianjin Institute of Industrial Biotechnology, Chinese Academy of Sciences, Tianjin, 300308 China; 3 State Key Laboratory of Microbial Metabolism, Joint International Research Laboratory of Metabolic & Developmental Sciences, and Laboratory of Molecular Biochemical Engineering, School of Life Sciences and Biotechnology, Shanghai Jiao Tong University, 800 Dong-chuan Road, Shanghai, 200240 China

**Keywords:** Synthetic biology, Metabolic engineering, Natural product synthesis, Comparative genomics

## Abstract

Type II ganoderic acids (GAs) produced by the traditional medicinal mushroom *Ganoderma* are a group of triterpenoids with superior biological activities. However, challenges in the genetic manipulation of the native producer, low level of accumulation in the farmed mushroom, the vulnerabilities of the farming-based supply chain, and the elusive biosynthetic pathway have hindered the efficient production of type II GAs. Here, we assemble the genome of type II GAs accumulating *G. lucidum* accession, screen cytochrome P450 enzymes (CYPs) identified from *G. lucidum* in baker’s yeast, identify key missing CYPs involved in type II GAs biosynthesis, and investigate the catalytic reaction sequence of a promiscuous CYP. Then, we engineer baker’s yeast for bioproduciton of GA-Y (**3**) and GA-Jb (**4**) and achieve their production at higher level than those from the farmed mushroom. Our findings facilitate the further deconvolution of the complex GA biosynthetic network and the development of microbial cell factories for producing GAs at commercial scale.

## Abstract (web summary)

The biosynthetic pathway of type II ganoderic acids (GAs) in *Ganoderma lucidum*, a traditional medicinal mushroom, is unknown. Here, the authors assemble the genome of type II GAs accumulating accession, identify CYPs involving in type II GAs biosynthesis, and achieve their production in engineered baker’s yeast.

## Introduction

The mushroom *Ganoderma* has been used for medicinal purposes for more than 2000 years in China and other Asian countries^1^. Of the various secondary metabolites identified from *Ganoderma* species, the lanostane-type triterpenoids—ganoderic acids (GAs)—are the main bioactive compounds^2^. A number of GAs have shown in vitro and in vivo anti-cancer and anti-metastasis activities, rendering them as promising candidates for anti-cancer drugs^3,4^. GAs are predominantly isolated from mushroom biomass, as direct chemical synthesis is greatly hindered by their stereochemical complexity^5^. However, the GA content of *Ganoderma* is extremely low^6–12^, and increasing the content of specific GAs is difficult due to technical challenges with the genetic manipulation of *Ganoderma*^13^ and a lack of knowledge of GA biosynthesis. Moreover, the mushroom-farming-based supply chain is susceptible to the climate^14^, insect pests^15^, the availability of agricultural land^16^, and global crises, including the ongoing coronavirus disease 2019 pandemic^17,18^. Synthetic biology approaches using heterologous microbes (e.g., the baker’s yeast *Saccharomyces cerevisiae*) to biosynthesize GAs may overcome the aforementioned challenges.

**Fig. 1:**
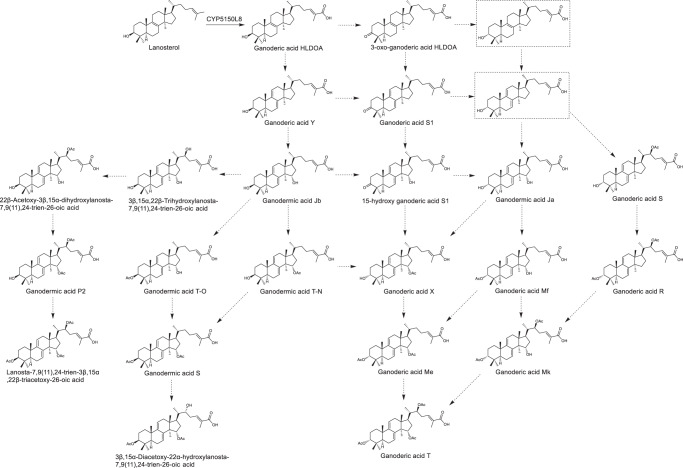
Proposed biosynthetic pathway of type II GAs from lanosterol. The solid arrow indicates that the catalytic activity of the enzyme has been confirmed. The dotted arrow indicates the proposed catalytic reactions. The dotted rectangle borders indicate the proposed intermediates. (24*E*)−3-oxo-5α-lanosta-7,9(11),24-trien-26-oic acid was named ganoderic acid S in previous studies^73,74^. To distinguish it from (24*E*)−3α-hydroxy-5α-lanosta-7,9(11),24-trien-22β-acetoxy-26-oic acid, a different compound previously reported with the same name^7,8,67^, (24*E*)−3-oxo-5α-lanosta-7,9(11),24-trien-26-oic acid is named ganoderic acid S1 in this paper.

GAs comprise a lanosterol skeleton with a series of post-modifications (e.g., hydroxylation, methylation, and acetylation). They are grouped into two types based on these modifications. Type II GAs have conjugated double bonds on their tetracyclic rings, while type I GAs have only one double bond on their tetracyclic rings^19^. Compared with type I GAs, type II GAs harbor more sophisticated modifications and exhibit stronger anti-tumor activities in general^2,20–24^. As such, their large-scale production has attracted extensive attention^25–27^. Although the biosynthetic pathway of lanosterol from acetyl-CoA has been characterized in *Ganoderma*, the enzymes responsible for the post-modifications of GAs remain elusive. In our previous study, CYP5150L8 was found to catalyze the three-step oxidation of lanosterol into the type I GA 3-hydroxy-lanosta-8,24-dien-26-oic acid (GA-HLDOA)^28^. GA-HLDOA was proposed to be transformed into a series of type II GAs (e.g., ganoderic acid Y (GA-Y), ganodermic acid Jb (GA-Jb), and ganoderic acid T (GA-T)) in *Ganoderma* through multiple post-modification steps that may involve many cytochrome P450 enzymes (CYPs, Fig. 1)^29,30^. The identification of CYP5150L8 enabled us to produce type I GAs in *S. cerevisiae*^28,31^. However, without knowing the key enzymes that transform the type I GA skeleton into type II GAs, the heterologous production of type II GAs remains impossible^32,33^.

Many efforts have been made for screening key enzymes in terpenoid biosynthesis^34–38^. To narrow down the candidates, diverse bioinformatic guided prioritization, including comparative transcriptomic analysis^39^, annotation and comparison of gene cluster^37,40,41^, protein family classification^38^, and standard sequence alignments^42^, were usually adopted prior to screening. On the other hand, functional identification of genes^36^ or clusters^34^ was also performed with trivial candidates’ prioritization. In our previous work, we have conducted a series of bioinformatics-guided prioritization of CYPs, and identified some functional CYPs (e.g., CYP5150L8^28^ and CYP5139G1^43^). However, we have not found any CYP functioning in the biosynthesis of type II GAs. The difficulty in mapping out the biosynthetic pathways of GAs may be attributed to two reasons. First, in basidiomycete mushrooms, the biosynthetic genes of secondary metabolites are usually scattered throughout the genome, making the bioinformatics-guided discovery of gene clusters challenging^44^. Second, for a complicated biosynthetic pathway that may involve multiple post-modifications on different sites, it is difficult to determine the exact sequence of modification steps. Thus, the use of an inappropriate substrate may preclude the discovery of the key enzyme with high specificity toward its natural substrate. Therefore, multiple substrates need to be considered when screening key enzymes involved in the later steps of GA biosynthesis.

In this work, to aid the search for appropriate substrates and the corresponding enzymes for the biosynthesis of type II GAs, we adopt a platform that is compatible with iterative screenings of heterologous enzymes and metabolic engineering. This platform uses a tunable expression strategy that has been shown to be effective in optimizing the biosynthesis of heterologous triterpenoids in *S. cerevisiae*^31,45^. Using this platform, we perform three rounds of screening of 158 CYPs from *Ganoderma*, employ different substrate-producing yeasts as screening host in each round, and identify several key enzymes, including two CYPs capable of converting type I GAs to type II GAs. Then, we engineer yeast for de novo biosynthesis of multiple bioactive GAs. Furthermore, we elucidate the biosynthetic pathway of a group of value-added GAs, which will facilitate the development of microbial cell factories to produce GAs at commercial scale.

## Results

### Identify CYP candidates from genomic and transcriptomic sequences of *G. lucidum* 5.616

To exhaustively search for CYPs involved in the biosynthesis of type II GAs, we performed genomic and transcriptomic sequencing of *Ganoderma* *lucidum* CGMCC 5.616, a *Ganoderma* strain capable of accumulating various type II GAs in shaking–static culture^8,46^. We annotated 215 CYP-coding genes and confirmed that 211 of them were transcribed during the shaking–static culture process (Supplementary Data 1 and 2). All of these CYPs were considered as CYP candidates for the biosynthesis of type II GAs (Supplementary Data 1).

### Screening key CYPs for GA biosynthesis

**Fig. 2:**
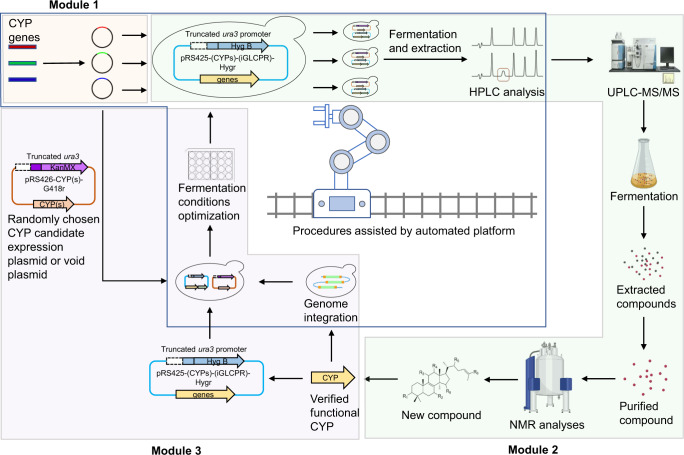
Schematic illustration of the iterative screening of functional CYPs facilitated by an automated system. The platform included three modules: the construction of CYP expression plasmids (module 1, highlighted in light pink), high-throughput in vivo screening of functional CYPs (module 2, highlighted in light green), and metabolic engineering of a yeast host compatible with the next round of screening (module 3, highlighted in light purple). In detail, the coding sequences of CYPs were amplified from cDNA of *G. lucidum* and cloned into the plasmid pRS426-HXT7p-FBA1t-G418r to form a CYP expression plasmid library. The corresponding plasmids were introduced into a yeast chassis. The resultant strains were then inoculated into 24-well plates for fermentation. The products were extracted from the cell pellet and then subjected to high-performance liquid chromatography (HPLC) and ultra-performance liquid chromatography-mass spectrometry (UPLC-MS) analyses. The target compounds were separated, purified, and subjected to nuclear magnetic resonance (NMR) analysis to confirm their structures. To generate a suitable chassis cell for the next round of screening, the identified CYP was either overexpressed via a plasmid or integrated into the chromosome. The fermentation conditions were then optimized to maximize substrate production for the next round of screening. The procedures in the blue frame were undertaken by the automated system.

To identify the key CYPs for GA biosynthesis from hundreds of candidates, we used an iterative screening platform assisted by an automated system. The platform included three modules: the construction of CYP expression plasmids (module 1), high-throughput in vivo screening of functional CYPs (module 2), and metabolic engineering of a yeast host compatible with the next round of screening (module 3). The automated system included an automated liquid handler and colony picker, and it facilitated polymerase chain reaction (PCR), DNA ligation, transformation, and the preparation of hundreds of fermentation samples simultaneously (Fig. 2).

To construct CYP expression plasmids, the coding regions of candidate CYPs were amplified (Supplementary Data 3) and individually cloned into the plasmid pRS426-HXT7p-FBA1t-G418r^31^, allowing tunable expression of CYP by changing the concentration of Geneticin (G418) during yeast fermentation^47^. To reduce the number of false positives during plasmid construction in *Escherichia coli*, the virulence gene *ccdB* was incorporated into the plasmid backbone^48^. The cDNA samples prepared from *G. lucidum* CGMCC 5.616 were used to amplify the 215 annotated CYP-coding genes. Facilitated by the system, several rounds of plasmid construction, with different PCR conditions, were performed in a high-throughput manner. As a result, 158 CYPs were successfully cloned, accounting for 73% of the CYP candidates. For in vivo screening of the functional CYPs, the CYP expression plasmids were individually introduced into an appropriate strain using the screening platform. The products were then extracted from the cell pellets in 24-well plates using the automated liquid handler. The cell extract was subjected to HPLC analysis. The samples of interest were then subjected to UPLC-MS analysis to determine whether the molecular weights of the compounds were consistent with those of GAs. To obtain sufficient quantities of the compounds for chemical structure determination, large-scale fermentation, extraction, and product purification were performed. The chemical structures of the purified compounds were determined by NMR analysis. After the functional identification of a CYP capable of producing GAs in the engineered yeast strain, the CYP was required to be either expressed via a plasmid other than pRS426-HXT7p-FBA1t-G418r or integrated into the yeast chromosome. In addition, a series of metabolic engineering steps was required to increase the production of the identified GAs, which then served as the substrates of CYPs in the next round of screening.

### Functional CYP screening in lanosterol-producing yeast

In our previous study, we screened 72 CYPs from *G. lucidum* 260125-1 and identified CYP5150L8 as a lanosterol oxidase involved in the biosynthesis of GA-HLDOA^28^. We first sought to determine whether there were other lanosterol oxidases among the 158 CYPs from *G. lucidum*. We initially generated yeast strain iGLCPR-r by introducing the plasmid pRS425-iGLCPR-Hygr into the lanosterol hyper-producing strain YL-T3 (Supplementary Data 4). YL-T3 was an engineered *S. cerevisiae* strain derived from BY4742 by the overexpression of hydroxy-3-methylglutaryl coenzyme A (*tHMG1p*), farnesyl diphosphate synthase (*Erg20p*), squalene synthase (*Erg9p*), and squalene epoxidase (*Erg1p*) through chromosomal incorporation, to achieve hyperproduction of lanosterol^28^, which is as a precursor of a diverse range of GAs. The plasmid pRS425-iGLCPR-Hygr contained an expression cassette of the CYP reductase iGLCPR, which is crucial for supporting the reactions mediated by *Ganoderma*-derived CYPs^31^. We previously demonstrated that the efficient production of GA-HLDOA in yeast might be achieved by optimizing the expression of CYP5150L8 and iGLCPR in the presence of 500 mg/L G418 and 300 mg/L hygromycin^31^. Herein, the void plasmid pRS426-HXT7p-FBA1t-G418r was first introduced into iGLCPR-r to generate a strict control strain, CK-r-iGLCPR-r, which produced 7.70 mg/L lanosterol after 120 h of fermentation under the aforementioned conditions, indicating that sufficient substrate was available to support the in vivo screening of a lanosterol oxidase. Therefore, yeast strain iGLCPR-r was adopted as the chassis strain.

**Fig. 3:**
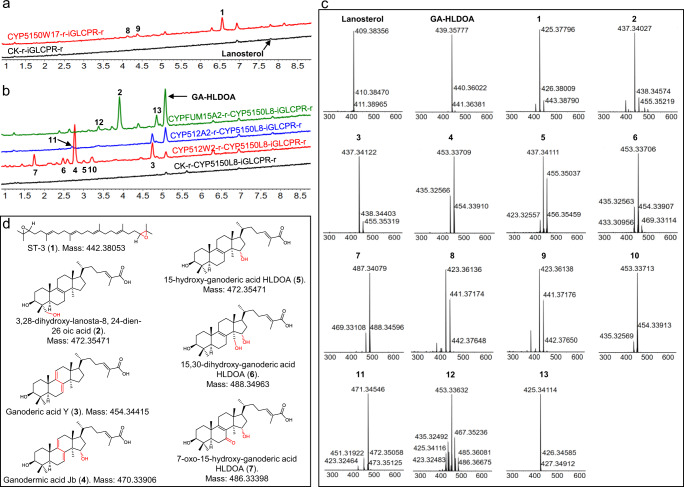
Functional CYP screening in strains iGLCPR-r and CYP5150L8-iGLCPR-r. **a** UPLC analysis of extracts of strain CYP5150W17-r-iGLCPR-r (red line) and the control strain CK-r-iGLCPR-r (black line); **b** UPLC analysis of extracts of strain CYPFUM15A2-r-CYP5150L8-iGLCPR-r (green line), strain CYP512A2-r-CYP5150L8-iGLCPR-r (blue line), strain CYP512W2-r-CYP5150L8-iGLCPR-r (red line), and the control strain CK-r-CYP5150L8-iGLCPR-r (black line); **c** MS spectra of compounds corresponding to peaks 1–13, as indicated in **a** and **b**. The y-axis represents the total ion current (TIC) intensity. **d** The chemical structures of **1–7**, as indicated in **a**–**c**.

A total of 158 CYP expression plasmids were individually introduced into strain iGLCPR-r using our system. Compared with the control strains CK-r-iGLCPR-r or CYP5148B6-r-iGLCPR-r, strain CYP5150W17-r-iGLCPR-r generated three new peaks in HPLC analysis after 120 h of fermentation (Fig. 3a), while strain CYP512A4-r-iGLCPR-r generated two new peaks (Supplementary Fig. 1a). The primary *m/z* values of these peaks were 423 (peaks 8 and 9), 425 (peaks 1 and 16), and 443 (peak 15; Fig. 3a, c, and Supplementary Fig. 1), which might correspond to the oxidation products of lanosterol. The compound corresponding to peak 1 was identified as 2,3;22,23-squalene dioxide (Fig. 3d and Supplementary Figs. 2–7), a linearized triterpenoid ST-3 identified in our previous study^49^. However, we did not obtain sufficient quantity of purified compounds corresponding to peaks 8, 9, 15, and 16, due to their low yields. For the other CYP-candidate-expressing strains, no new peaks that likely correspond to oxidized products of lanosterol were observed.

### Identification of key enzymes for type II GA biosynthesis

Of the CYPs identified in the lanosterol-producing yeast, only CYP5150L8 was found to catalyze lanosterol to form GA-HLDOA. To construct a GA-HLDOA-producing strain compatible with the next round of screening, an expression cassette of CYP5150L8 was incorporated into plasmid pRS425-iGLCPR-Hygr^31^ and introduced into *S. cerevisiae* YL-T3 to generate strain CYP5150L8-iGLCPR-r. As indicated in our previous study, the copy number of plasmids carrying *cyp5150l8* and *iglcpr* might be coordinately regulated by changing the concentration of hygromycin^31,47^. To maximize GA-HLDOA production for downstream screening, a CYP expression plasmid, pRS426-CYP5035C11-G418r, was randomly chosen and introduced into CYP5150L8-iGLCPR-r to generate strain CYP5035C11-r-CYP5150L8-iGLCPR-r. We optimized GA-HLDOA production by the engineered strain by changing the hygromycin concentration during fermentation (Supplementary Fig. 8). When the hygromycin concentration was 300 mg/L, GA-HLDOA production was 6.94 mg/L, which was significantly higher than the level of GA-HLDOA production in the presence of 100 mg/L hygromycin. When the hygromycin concentration increased from 300 to 1000 mg/L, no significant changes in GA-HLDOA production were observed. Thus, 500 mg/L G418 and 300 mg/L hygromycin were used in the fermentation culture for strain CYP5150L8-iGLCPR-r in subsequent screening experiments.

One hundred and fifty-eight CYP expression plasmids were individually introduced into strain CYP5150L8-iGLCPR-r to generate the corresponding CYP-expressing strains using the system. Plasmid pRS426-HXT7p-FBA1t-G418r was also introduced into strain CYP5150L8-iGLCPR-r to generate the control strain CK-r-CYP5150L8-iGLCPR-r. Among all the CYP-expressing candidates, strains CYPFUM15A2-r-CYP5150L8-iGLCPR-r, CYP512A2-r-CYP5150L8-iGLCPR-r, and CYP512W2-r-CYP5150L8-iGLCPR-r showed new peaks in HPLC analysis (Fig. 3b). For strain CYPFUM15A2-r-CYP5150L8-iGLCPR-r, three new peaks with primary *m/z* values of 437 (peak 2), 453 (peak 12), and 425 (peak 13) were detected (Fig. 3b, c). For strain CYP512A2-r-CYP5150L8-iGLCP-r, two new peaks with primary *m/z* values of 437 (peak 3) and 471 (peak 11) were detected (Fig. 3b, c). Meanwhile, for strain CYP512W2-r-CYP5150L8-iGLCPR-r, six new peaks with primary *m/z* values of 437 (peaks 3 and 5), 453 (peaks 4, 6 and 10), and 487 (peak 7) were detected (Fig. 3b, c). The peak with the *m/z* value of 425 (peak 13) likely corresponded to the oxidation product of squalene, 2,3-oxiosqualene, or lanosterol. The new peaks with *m/z* values of 437 (peaks 2, 3 and 5) likely corresponded to the oxidation products of GA-HLDOA, with one hydroxyl group added. The new peak with the *m/z* value of 487 (peak 7) likely corresponded to the oxidation product of GA-HLDOA, with three hydroxyl groups added. Further, the new peaks with *m/z* values of 453 and 471 (peaks 4, 6, 10, 11, and 12) likely corresponded to the oxidation products of GA-HLDOA, with two hydroxyl groups added.

To determine the structures of these compounds, we performed 9.6 L fermentations of strains CYPFUM15A2-r-CYP5150L8-iGLCPR-r, CYP512A2-r-CYP5150L8-iGLCPR-r, and CYP512W2-r-CYP5150L8-iGLCPR-r. Four milligrams of **2** (corresponding to peak 2) was finally purified from the fermentation extracts of strain CYPFUM15A2-r-CYP5150L8-iGLCPR-r; 3 mg of **3** (corresponding to peak 3) was purified from the fermentation extracts of strain CYP512A2-r-CYP5150L8-iGLCPR-r; and approximately 150 mg of **4** (corresponding to peak 4), 1 mg of **5** (corresponding to peak 5), 1.2 mg of **6** (corresponding to peak 6), and 2.1 mg of **7** (corresponding to peak 7) were purified from the fermentation extracts of strain CYP512W2-r-CYP5150L8-iGLCPR-r. However, we did not obtain a sufficient quantity of purified compounds corresponding to peaks 8-13 due to their low yield or instability.

Compound **2** was identified as 3,28-dihydroxy-lanosta-8,24-dien-26-oic acid by NMR analysis (Supplementary Fig. 9–14), which was consistent with the product of CYP5139G1 reported in our recent study^43^. The similarity between **3** and GA-HLDOA in the ^1^H (Supplementary Fig. 15) and ^13^C (Supplementary Fig. 16) NMR spectra indicates that the structure of **3** was derived from GA-HLDOA. There were six olefinic carbons (δ_C_ 145.95, 145.67, 142.57, 126.48, 120.34, and 116.20) in the ^13^C NMR spectrum of **3**, while GA-HLDOA had four, indicating that **3** had three double bonds. There were three olefinic CHs (δ_C_, δ_H_)—(145.67, 6.90), (120.34, 5.48), and (116.20, 5.32)—in the distortions enhancement by polarization transfer (DEPT)−135 (Supplementary Fig. 17) and heteronuclear single quantum coherence (HSQC, Supplementary Fig. 19) spectra. The presence of olefinic CH (δ_C_ 145.67, δ_H_ 6.90) was consistent with the C-24 of GA-HLDOA. Two olefinic Cs (δ_C_ 145.95 and 142.57) had heteronuclear multiple bond correlation (HMBC, Supplementary Fig. 20) with H-19 and H-30, while two olefinic protons (Hs) (δ_H_ 5.48 and 5.32) were split into doublet peaks in the ^1^H NMR spectrum. All NMR spectra (Supplementary Figs. 15–20) indicated that **3** contained conjugated double bonds on its tetracyclic rings. We concluded that the chemical structure of **3** is 3-hydroxy-lanosta-7(8),9(11),24-trien-26-oic acid, which was identical to the structure of GA-Y^50^.

The NMR spectra (Supplementary Figs. 21–26) of **4** were similar to those of **3**. The differences were the presence of H-15 (δ_H_ 4.23) in the ^1^H NMR spectrum (Supplementary Fig. 21) and C-15 (δ_C_ 75.27) in the ^13^C NMR spectrum (Supplementary Fig. 22), indicating an extra hydroxyl group at C-15. The nuclear Overhauser effect spectroscopy (Supplementary Fig. 27) correlation between H-15 (δ_H_ 4.23) and H-18 (δ_H_ 0.65) indicated that the hydroxyl group at C-15 was 15α-OH. Thus, the chemical structure of **4** was 15-hydroxyGA-Y, consistent with the structure of GA-Jb^2,51,52^.

The NMR spectra (Supplementary Figs. 28–33) of **5** were similar to those of GA-HLDOA. The differences were H-15 (δ_H_ 4.22) in the ^1^H NMR spectrum (Supplementary Fig. 28) and C-15 (δ_C_ 73.63) in the ^13^C NMR spectrum (Supplementary Fig. 29). In addition, the H-15 (δ_H_ 4.22) and C-15 (δ_C_ 73.63) signals were similar to those of **4**. These results indicated that **5** was 15-hydroxy-GA-HLDOA, which was not reported before.

The NMR spectra (Supplementary Figs. 34–39) of **6** were similar to those of **5**. In contrast, **6** had six methyl groups in the ^1^H NMR spectrum (Supplementary Fig. 34), one less than the number of methyl groups in **5**. The H-30 (3.66 d, *J* = 11.0 Hz, 4.02 d, *J* = 11.1 Hz, 2H) in the ^1^H NMR spectrum and the C-30 (δ_C_ 66.63) in the ^13^C NMR spectrum (Supplementary Fig. 35) indicated that a hydroxyl group existed at C-30. The chemical structure of **6** was 15,30-dihydroxy-GA-HLDOA, which was also not reported before.

The NMR spectra (Supplementary Figs. 40–45) of **7** were similar to those of **5**. The DEPT-135 (Supplementary Fig. 42) and ^1^H NMR spectra (Supplementary Fig. 40) of **7** indicated that it had nine methylene groups, one less than the number of methylene groups in GA-HLDOA. Meanwhile, a carbon with δ_C_ 145.04 appeared in the ^13^C NMR spectrum (Supplementary Fig. 41), suggesting that a methylene group of GA-HLDOA was transformed to a ketone group. The HMBC (Supplementary Fig. 45) between the carbon (δ_C_ 145.04) and H-6 (2.50 m, 2.56 m, 2H) indicated that the C-7 was a ketone group. The chemical structure of **7** was identified as 7-oxo-15-hydroxy-GA-HLDOA, which was consistent with 7-oxo-GA-Z_3_ as previously reported^12^. The ^13^C-NMR and ^1^H-NMR data for all of the identified compounds were shown in Supplementary Data 5.

Compounds **3** and **4** are type II GAs with conjugated double bonds on the tetracyclic rings, suggesting the important roles of CYP512A2 and CYP512W2 in the biosynthesis of type II GAs. When CYP512A2- and CYP512W2-containing yeast microsomes were adopted for P450 spectral analysis, significant CO-shifts from 420 nm to 450 nm were detected, suggesting that these mushroom-derived CYPs were properly folded in *S. cerevisiae* (Supplementary Fig. 46). Meanwhile, we did not detect **3** or **4** in the fermentation extracts of strains CYP512A2-r-iGLCPR-r or CYP512W2-r-iGLCPR-r, suggesting that lanosterol is not a substrate of these two CYPs. In addition to **3** produced by strain CYP512A2-r-CYP5150L8-iGLCPR-r, strain CYP512W2-r-CYP5150L8-iGLCPR-r produced more complex type II GA (**4**) and type I GAs (e.g., **5**, **6**, and **7**) (Fig. 3b–d and Supplementary Table 1), indicating the promiscuity of CYP512W2 in GA biosynthesis.

### The biosynthetic steps in CYP512W2-catalyzed conversion of GA-HLDOA to type II GAs

**Fig. 4:**
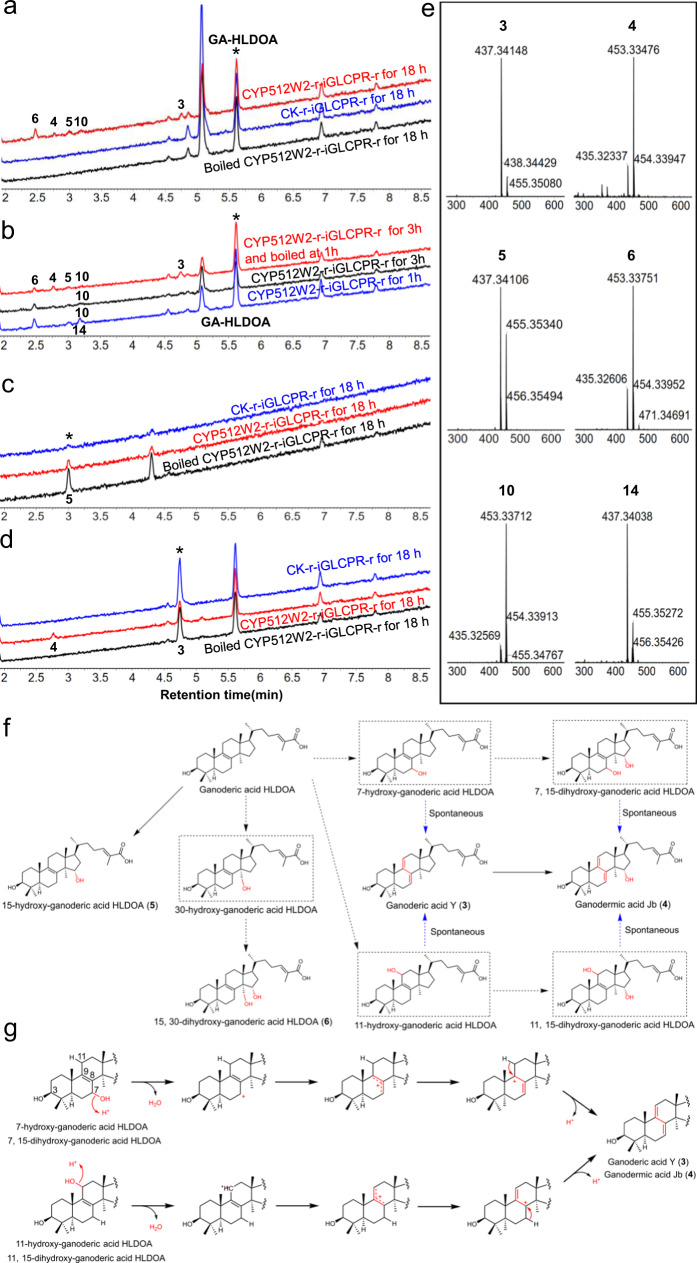
In vitro enzymatic reactions of CYP512W2. Microsomes were prepared from strain CYP512W2-r-iGLCPR-r or the control strain CK-r-iGLCPR-r. The RT was 18 h, unless specified otherwise. The reaction products were analyzed by UPLC-MS. **a**, **b** GA-HLDOA was used as the substrate; **c 3** was used as substrate; **d 5** was used as substrate; **e** MS spectra of peaks 3, 4, 5, 6, 10, and 14, as indicated in **a** to **d**. The y-axis represents the TIC intensity; **f** The proposed reaction sequence for the biosynthesis of type II GAs by CYP512W2. The solid lines indicate the catalytic reactions confirmed in in vitro enzymatic reactions. The dashed lines indicate the catalytic reactions that have been speculated from the results of the in vitro enzymatic reactions, with the blue lines indicating the spontaneous reactions. The dotted rectangles indicate the compounds that have not been confirmed due to the instability of their chemical structure or their rapid conversion in CYP512W2-catalyzed enzymatic reactions. **g** The proposed reaction mechanism for the spontaneous formation of conjugated double bonds of type II GAs.

To understand the biosynthetic steps in the CYP512W2-catalyzed conversion of GA-HLDOA to type II GAs, we prepared CYP512W2-containing yeast microsomes and performed in vitro reactions using the purified compounds as substrates. When microsomes from CYP512W2-r-iGLCPR-r were incubated with GA-HLDOA for 18 h, peaks 3 (4.75 min; *m/z*, 437), 4 (2.77 min; *m/z*, 453), 5 (3.00 min; *m/z*, 437), 6 (2.48 min; *m/z*, 453), and 10 (3.21 min; *m/z*, 453) were newly generated (Fig. 4a, e). The retention times (RTs) and mass spectra of peaks 3, 4, 5, and 6 were consistent with **3**, **4**, **5**, and **6** identified in the in vivo screening experiments (Figs. 3b, c and 4a, b, e). Peak 10 was observed in both the in vivo screening experiments and the in vitro reactions (Figs. 3b, c and 4b, e). However, we did not obtain a sufficient quantity of purified product corresponding to peak 10 from the large-scale fermentation broth for NMR analysis. Meanwhile, peak 7 (1.74 min;*m/z*, 487), which was detected in the fermentation extracts, was not observed in the in vitro enzymatic assays, implying that **7** was not likely to be the reaction product (Figs. 3b, 4a, b). Based on the structural differences between **7** and **5**, **7** was probably derived from **5**, with a ketone group added at C-7, or derived from an intermediate (7,15-dihydroxy-GA-HLDOA) with a hydroxyl group oxidized to a ketone group at C-7 by an endogenous yeast enzyme. None of these peaks appeared in the reactions using microsomes from the control strain CK-r-iGLCPR-r or the heat-inactivated CYP512W2-containing microsomes (Fig. 4a). Taken together, these results suggested that **3**, **4**, **5**, and **6** and the compound corresponding to peak 10 were the reaction products of CYP512W2 when GA-HLDOA was used as the substrate (Fig. 4f).

To determine the order of CYP512W2-catalyzed reactions, we explored the time course of the reaction (Fig. 4b). After 1 h, peaks 5, 6, 10, and 14 (3.18 min; *m/z*, 437) rapidly appeared and exhibited slightly higher concentrations than those after 3 h, while peaks 3 and 4 increased slightly after 3 h (Fig. 4b). Unexpectedly, when the microsome was heat-inactivated, cooled at RT for 1 h and was then incubated with the substrate for another 2 h, considerable increases in peaks 3 and 4 and decreases in peaks 14 and 6 were observed compared with those after 1 h and 3 h of RTs (Fig. 4b). The newly generated peak 14 was only detected under these conditions (Fig. 4b). These results suggested that peaks 14, 5, 6, and 10 were likely to be the first reaction products, and that the compounds corresponding to peaks 14 and 6 may be unstable. The primary *m/z* value of GA-HLDOA was 439, suggesting that the compound corresponding to peak 14 (3.18 min; *m/z*, 437) was probably derived from GA-HLDOA by introducing a hydroxyl group. We also observed a significant increase in peak 3, accompanied by a decrease in peak 14, when CYP512W2-containing microsomes were incubated with GA-HLDOA for more than 1 h (Fig. 4b). **3** might be spontaneously transformed from either 7-hydroxy-GA-HLDOA or 11-hydroxy-GA-HLDOA, which had *m/z* values identical to peak 14 (Fig. 4b, e). However, **6** isolated from the fermentation extracts was quite stable, suggesting that two compounds with the same RT and primary *m/z* value of 453 might be present in peak 6 in the in vitro enzymatic assays (Fig. 4a, b). One of these compounds was **6**, while the other one, with an unstable chemical structure, might be either 7,15-dihydroxy-GA-HLDOA or 11,15-dihydroxy-GA-HLDOA, the oxidation products derived from peak 14. Similarly, **4**, corresponding to peak 4, might be spontaneously transformed from 7,15-dihydroxy-GA-HLDOA or 11,15-dihydroxy-GA-HLDOA. Interestingly, the changes in peaks 3 and 4 also indicated that the microsome heat-inactivation process might significantly promote spontaneous transformation to generate the compounds corresponding to peaks 3 and 4 (Fig. 4b).

In addition to spontaneous transformation, we sought to determine whether **4** might also be generated by the CYP512W2-mediated enzymatic reaction, and which step, whether C-15 hydroxylation or conjugated double bonds formation, came first. To answer these questions, **5** was incubated with CYP512W2-containing microsomes. We did not detect any new peaks from these extracts (Fig. 4c), which indicated that neither **4** nor **6** was converted from **5** by the CYP512W2-mediated enzymatic reaction. One possible route for **6** formation was that CYP512W2 first hydroxylated GA-HLDOA at C-30 to form an intermediate (30-hydroxy-GA-HLDOA) and then further oxidized this intermediate into **6** (Fig. 4f). As the intermediate was not detected in in vivo or in vitro experiments, we speculated that the reaction from the intermediate to **6** was too fast, with tight binding between the CYP and the intermediate. When the CYP512W2-containing microsome was incubated with **3**, peak 4 (2.77 min; *m/z*, 453; corresponding to **4**) was generated (Fig. 4d, e). In contrast, we did not detect peak 4 when CK-r-iGLCPR-r-containing microsomes or heat-inactivated CYP512W2-containing microsomes were used. These results indicate that **4** might be converted from **3** via a CYP512W2-mediated enzymatic reaction. As we did not obtain an unstable product corresponding to peak 14 to use in an in vitro enzymatic assay, and we could not prevent the product corresponding to peak 14 from being transformed to **3**, it remained unknown whether **3** might also be directly converted from the CYP512W2-mediated enzymatic reaction.

In summary, CYP512W2 probably catalyzed the formation of the unstable product 7-hydroxy-GA-HLDOA or 11-hydroxy-GA-HLDOA from GA-HLDOA, and this product was further spontaneously transformed to generate **3**. CYP512W2 might further catalyze such unstable intermediate to form a second unstable intermediate, 7,15-dihydroxy-GA-HLDOA or 11,15-dihydroxy-GA-HLDOA, which was spontaneously transformed to generate **4**. Meanwhile, CYP512W2 was also able to catalyze the formation of **4** from **3** and the formation of **5** and **6** from GA-HLDOA. However, CYP512W2 could not catalyze the formation of **4** or **6** from **5** (Fig. 4f).

The processes that CYP512W2 used to catalyze the formation of **5**, **6**, and the four unstable intermediates from GA-HLDOA are typical fungal P450-mediated reactions that occurred via the canonical oxygen rebound mechanism^53^. However, it remained unclear how the double bonds in type I GAs (the four aforementioned unstable intermediates) were transformed to generate the conjugated double bonds present in type II GAs (**3** and **4**). We speculated that the formation of the conjugated double bonds (C7=C8, C9=C11) might go through a cascade reaction of carbon cation generation, migration and elimination. The hydroxyl groups OH-7 or OH-11 were assumed to be protonated and converted into carbocation intermediates. They further migrated to C-8 or C-9 to give rise to the corresponding carbocation intermediates and formed the conjugated double bonds via elimination (Fig. 4g).

### Engineering GA-Y- and GA-Jb-producing yeast strains for subsequent screening

**Fig. 5:**
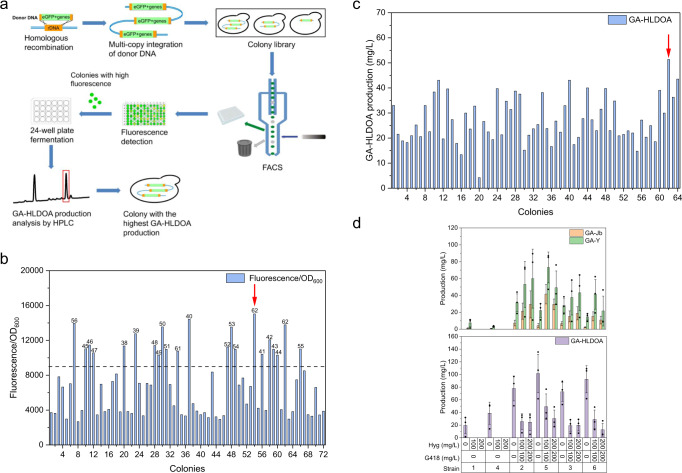
Integration of the CYP5150L8 and iGLCPR expression cassettes into yeast rDNA loci promotes the production of **3** and **4**. **a** Schematic illustration of screening of the GA-HLDOA-producing strain. Expression cassettes of eGFP, TRP1, CYP5150L8, and iGLCPR flanked by 0.55–0.70 kb homologous recombination regions were used as donors for integration at the rDNA loci. Recombinant clones with high green fluorescence signal intensity were isolated and selected for fermentation tests. **b** The fluorescence signal of partial clones. The fluorescence signal is shown as specific units per OD_600_. The dotted line indicates that the fluorescence signal is 9000. Clones with fluorescence signals greater than 9,000 were selected for fermentation tests. The number above the dotted line indicates the name of the selected clone. **c** Production of GA-HLDOA after 120 h of fermentation using the selected clones. **d** Production of **4**, **3**, and GA-HLDOA after 120 h of fermentation using the engineered strains. Strain 1, SC62-CYP512W2-r; strain 2, SC62-CK-r-CYP512W2-r; strain 3, SC62-CYP5037B21-r-CYP512W2-r; strain 4, SC62-CYP512W2-iGLCPR-r; strain 5, SC62-CK-r-CYP512W2-iGLCPR-r; and strain 6, SC62-CYP5037B21-r-CYP512W2-iGLCPR-r. All data represent the mean of three biologically independent samples (solid circles in **d**), and the error bars show the standard deviation. Source data are provided as a Source Data file.

As **3** and **4** were proposed to be the precursors of multiple type II GAs with more sophisticated post-modifications (Fig. 1) and superior biological activities^20,21,24,50,54–56^, we sought to engineer a yeast strain capable of producing sufficient quantities of **3** and **4** for subsequent screening. However, after a series of efforts, the production levels of **3** and **4** were still not sufficient to facilitate HPLC-based screening (Supplementary Note 1 and Supplementary Fig. 47). Finally, we integrated CYP5150L8 and iGLCPR expression cassettes at rDNA loci using simple homologous recombination (Fig. 5a). An enhanced green fluorescent protein (eGFP) expression cassette was included in the donor, allowing for the use of fluorescence-activated cell sorting (FACS) to screen transformants integrated with the CYP5150L8 and iGLCPR expression cassettes. A recombinant clone with high green florescence signal intensity might indicate the successful integration of multiple CYP5150L8 and iGLCPR cassettes. More than 5000 transformants were obtained and subjected to FACS analysis (Fig. 5a), and 960 cells with high fluorescence signal were individually collected and cultured. Of these cells, 64 colonies with fluorescence/optical density at 600 nm (OD_600_) ratios higher than 9000 were picked to perform fermentation and HPLC analysis (Fig. 5b). Finally, we obtained a colony named SC62, which showed the highest fluorescence/OD_600_ ratio and GA-HLDOA production of 51.36 mg/L (Fig. 5b, c).

We then sought to express CYP512W2 in strain SC62 to generate stable production of **3** and **4**. As iGLCPR, which had been integrated into the chromosome, functioned well to support the enzymatic activity of CYPs in SC62 (Supplementary Note 2, Fig. 5d and Supplementary Fig. 48), we introduced plasmid pRS425-HXT7p-CYP512W2-FBA1t-Hygr into SC62 to generate strain SC62-CYP512W2-r. Furthermore, we investigated whether overexpressing CYP512W2 or CYP512A2 in SC62-CYP512W2-r would further improve the production of **3** and **4**. However, the production of **3** and **4** was not significantly enhanced under these conditions (Supplementary Fig. 49). Strain SC62-CYP512W2-r was considered as a suitable chassis to conduct the next round of functional CYP screening. Meanwhile, the fermentation media were supplemented with 200 mg/L of G418 and hygromycin to ensure the efficient biosynthesis of **3** and **4** (Supplementary Note 2, Fig. 5d and Supplementary Fig. 48). However, after the individual introduction of 158 CYP expression plasmids into strain SC62-CYP512W2-r, we did not detect any new HPLC peaks from the fermentation extracts, compared with those from the control strain SC62-CK-r-CYP512W2-r.

### Type II GA production in shake-flask fermentation

**Fig. 6:**
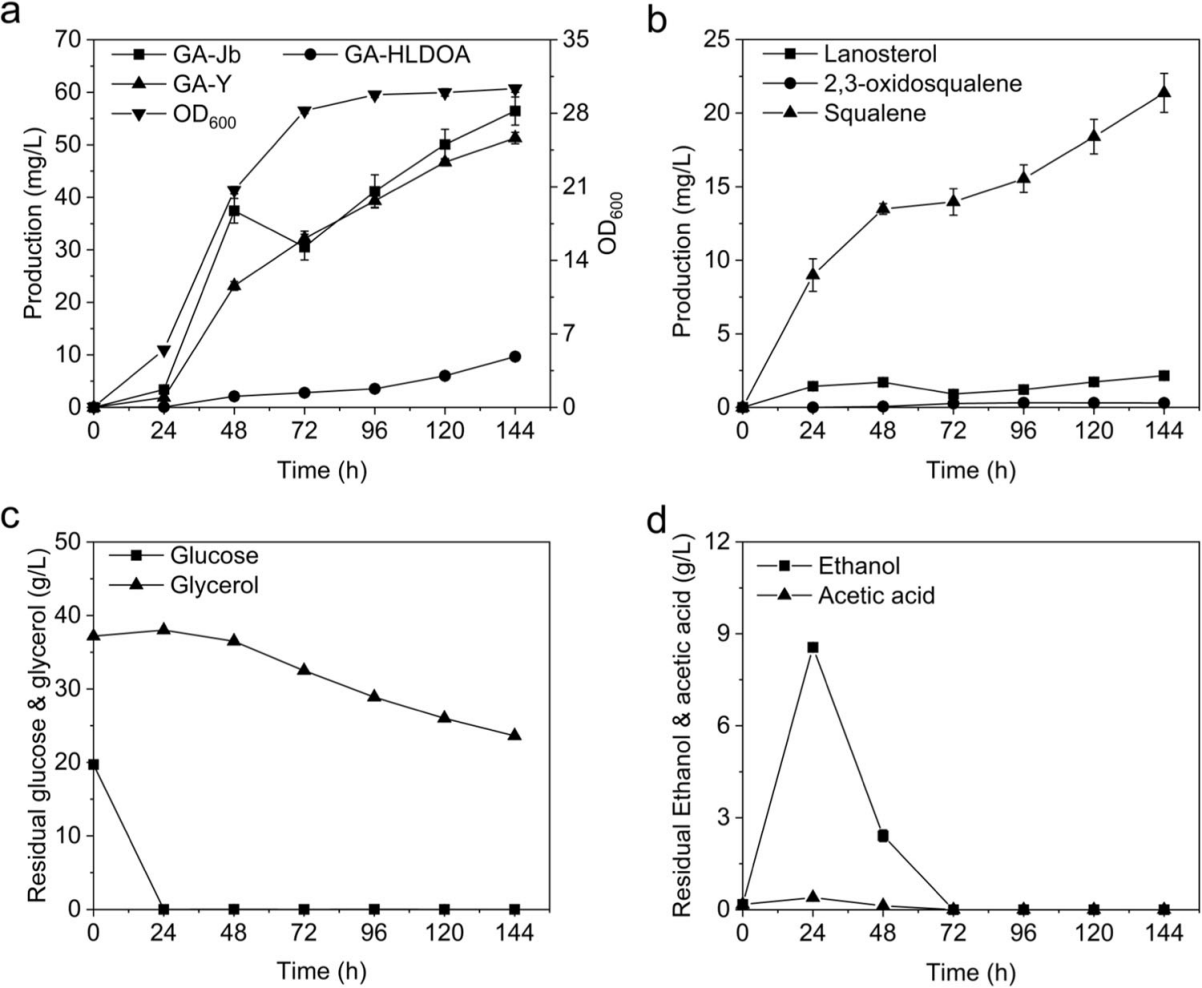
Fermentation behavior of strain CYP512W2-r-CYP5150L8-iGLCPR-r in a shake-flask system. Time profiles of **a** cell growth and **4**, **3**, and GA-HLDOA production; **b** the accumulation of lanosterol, 2,3-oxidosqualene, and squalene; **c** residual glucose and glycerol; and **d** ethanol and acetic acid concentrations are shown. All data represent the mean of four biologically independent samples, and the error bars show the standard deviation. Source data are provided as a Source Data file.

Subsequently, we investigated the fermentation behavior of the **3-** and **4-**producing strain CYP512W2-r-CYP5150L8-iGLCPR-r in a shake-flask system. Cell growth and the production of **3** and **4** increased slowly during the first 24 h (Fig. 6a), when squalene and ethanol accumulated rapidly (Fig. 6b, d), and this was accompanied by rapid glucose exhaustion (Fig. 6c). From 24 to 48 h, the carbon sources ethanol and acetic acid were rapidly consumed (Fig. 6d). The strain grew very fast during this period (Fig. 6a), while the production of **3** and **4** increased significantly (Fig. 6a), indicating that the consumption of carbon sources transitioned from glucose to ethanol and acetic acid, which promoted cell growth and the production of **3** and **4**. From 48 to 72 h, ethanol and acetic acid were gradually exhausted (Fig. 6d), which was accompanied by an increase in the rate of glycerol consumption (Fig. 6c), but a slight reduction in the production of **4** (Fig. 6a). During this period, the carbon sources gradually transitioned from ethanol and acetic acid to glycerol. From 72 to 144 h, slow consumption of glycerol was observed (Fig. 6c) and the strain entered a plateau phase, during which the production of **3** and **4** gradually increased, but at a relatively lower rate (Fig. 6a). After 144 h of fermentation, strain CYP512W2-r-CYP5150L8-iGLCPR-r was able to produce 9.66 mg/L of GA-HLDOA, 51.30 mg/L of **3**, and 56.44 mg/L of **4** (Fig. 6a). However, 21.37 mg/L of squalene and more than 20 g/L of glycerol remained in the culture media (Fig. 6b, c), indicating that re-engineering squalene transformation to 2,3-oxidosqualene and glycerol metabolism might be considered as future metabolic engineering strategies to enhance the bioproduction of type II GAs (**3** and **4**) in *S. cerevisiae*.

## Discussion

The mushroom *Ganoderma* has been used as a key medicinal ingredient in China and other Asian countries for thousands of years, and it has even become a cultural totem for health and fortune. However, its supply still relies on mushroom farming, as it did thousands of years ago, and the yields of most key GAs are at mg/kg of dry weight (DW) levels (Supplementary Table 2). Synthetic biology is an alternative strategy to synthesize and supply scarce bioactive compounds from plants and fungi. Nevertheless, most key enzymes in GA biosynthesis pathways remain unknown, rendering their heterologous production impossible. Here, we reported the discovery of key CYPs capable of converting type I GAs to type II GAs, which is a critical node in the biosynthesis of hundreds of GAs. With minimum metabolic engineering, we achieved the heterologous production of key type II GAs, such as **3** and **4**, at 5.45 g/kg-DW and 6.00 g/kg-DW, respectively. These yields were one to four orders of magnitude higher than the yields from farmed mushrooms (Supplementary Table 2). Meanwhile, their production efficiency was two to five orders of magnitude higher than their production efficiency from farmed mushrooms (Supplementary Table 2). **3** has multiple bioactivities, including inhibition of 3-hydroxy-3-methylglutaryl-coenzyme A reductase^20^ and acetylcholinesterase^55^ in pig liver microsomes, inhibition of the proliferation of HeLa human cervical carcinoma cells^21,57^, and cytotoxicity against human K562 cells^20^, and **4** can activate phospholipases C and A_2_ and promote human platelet aggregation^56^. In addition, we found another CYP that efficiently produced **1**, which effectively inhibits the proliferation of non-small cell lung cancer cells^45^. Therefore, our findings pave the way for advanced strain engineering and the production of GAs in sufficient quantities to allow further clinical research or potential industrial production.

In addition to the heterologous production of previously reported key GAs, we also found two GAs **5** and **6** that, to the best of our knowledge, were not reported before. This finding demonstrates another fascinating advantage of the synthetic biology approach. Analyzing each potential enzyme and reconstructing biosynthetic pathways may allow the identification of new intermediates that have been missed using traditional natural product discovery paradigms because of their low quantity. The sufficient amount of compounds produced in this study may offer opportunity to conduct assays that could not be performed before, which may reveal previously unknown bioactivities.

Our systematic screening identified multiple pairs of CYPs capable of producing the same compound. Some of these isoenzymes belonged to the same CYP family, while others did not. Specifically, CYP5139G1 and CYPFUM15A2, which convert GA-HLDOA to **2**, belong to the FUM15-like cytochrome P450 subfamily^30,58,59^. CYP512W2 and CYP512A2, which convert GA-HLDOA to **3**, belong to the CYP512 family, and CYP505D13 and CYP5150W17, which are capable of biosynthesizing **1**, belong to the CYP505 and CYP5150 family, respectively^30^. Of particular note, CYP512W2, but not CYP512A2, was able to generate a series of other GAs in addition to **3**. These results imply the great catalytic versatility and irregularity of fungal-derived CYPs.

After screening *G. lucidum*-derived CYPs in lanosterol-producing yeast, we discovered that two CYPs (CYP505D13^49^ and CYP5150W17) oxidized the precursors of lanosterol (squalene and 2,3-oxidosqualene) into **1**, and only one CYP (CYP5150L8) was a lanosterol oxidase^28^. Therefore, we speculated that **1** is the direct substrate of the cyclase from *G. lucidum*. The circulation of **1** in *G. lucidum* may produce a skeleton harboring an epoxy group at C-24 and C-25. This skeleton may serve as a precursor for the biosynthesis of a series of GAs (e.g., inonotsuoxide B^60^, lucidumol B^10,61^, ganodercochlearin A^60^, ganodercochlearin B^60^, and epoxyganoderiol C^62^). Screening cyclases and CYPs in yeast strains capable of producing **1**, using the iterative screening strategy demonstrated in this study, may allow us to confirm this speculation.

Although we explored the processes of the CYP512W2-catalyzed conversion of GA-HLDOA to type II GAs, some open-ended questions remain. First, we speculate that **4** and **3** are spontaneously formed from unstable intermediates with a hydroxyl group at either C-7 or C-11. However, it is not known whether C7 or C11 or both are the preferred modification sites. Second, in addition to spontaneous formation, it remains unknown whether such processes are also mediated by the enzymatic reaction catalyzed by CYP512W2. Third, **4** can be generated from **3** via a CYP512W2-catalyzed reaction and probably from the second unstable intermediates 7,15-dihydroxy-GA-HLDOA or 11,15-dihydroxy-GA-HLDOA through spontaneous conversion. We speculate that competition exists between these two routes, but it remains unknown which route is preferred. If the first unstable intermediate tends to form **3**, it may not be available to serve as the precursor of the second unstable intermediate, which allows the formation of **4**. It is difficult to pinpoint which route exists or if both routes exist, due to the inability to isolate such unstable intermediates.

To the best of our knowledge, the simultaneous introduction of conjugated double bonds and hydroxyl groups by one CYP has rarely been reported. An *Aspergillus terreus*-derived CYP, LovA, catalyzes the formation of monacolin J acid from 4a,5-dihydromonacolin L acid by sequentially forming conjugated double bonds at C-4 and C-5 and then introducing a hydroxyl group at C-8^63^. However, we did not observe a catalytic priority of CYP512W2 at C-15 and C-7 (or C-11), implying a broader substrate scope of CYP512W2. In another aspect, only one CYP512 family protein involved in GA synthesis, CYP512U6 from *G. lucidum* strain T29, has been functionally characterized^64^. Both CYP512U6 and CYP512W2 exhibit a relatively broad substrate scope^64^ (Figs. 3b, d, and 4f). Nonetheless, CYP512W2 has greater catalytic diversity than CYP512U6. CYP512U6 prefers to hydroxylate GA-DM and GA-TR at C-23, while CYP512W2 is likely to modify GAs at C-15, C-7 (or C-11), and C-30 (Fig. 4f).

Unexpectedly, the expression of CYPs in the **3**- and **4**-producing strain SC62-CYP512W2-r did not generate oxidized **3** or **4**, suggesting that they were not likely to be suitable substrates to form downstream GAs. It should be noted that GAs from the cultured mycelia of *G. lucidum*, including GA-T and GA-Me, usually harbor the α configuration at position C-3^65^. Unlike those GAs, GA-HLDOA, **3**, and **4** have a β configuration at position C-3. Our recent study demonstrated that GA-HLDOA was converted to 3-oxo-lanosta-8,24-dien-26-oic acid (OLDOA) by a crude enzyme extract from *G. lucidum*^66^. Compared with the chemical structure of GA-HLDOA, the chirality disappears at position C-3 of OLDOA. Therefore, we speculated that the β-hydroxyl group at C-3 underwent a chirality conversion before transforming into downstream GAs. Two enzymes might be involved in this chirality conversion^65,67^. One is an oxidase capable of oxidizing the β-hydroxyl group into a ketone group at C-3, the activity of which was observed in our previous study^66^. Another possible enzyme is a reductase capable of reducing the ketone group into an α-hydroxyl group at the same position. Using enzymatic-activity-guided protein purification, we are currently isolating the C-3 oxidase from *G. lucidum*. Using the power of the screening platform employed in this study, more post-modifications of type II GAs are expected to be elucidated in future studies.

## Methods

### Chemicals and enzymes

GA-HLDOA, 15-hydroxy-GA-HLDOA, **3**, and **4** were obtained by extraction and purification of yeast fermentation cultures, while the other chemicals were purchased from Sinopharm Chemical Reagent (Shanghai, China; glucose, glycerol, ethanol, and acetic acid) or Avanti Polar Lipids (Birmingham, AL, USA; lansterol). Q5 High-Fidelity DNA Polymerase (New England BioLabs, Ipswich, MA, USA), TransStart FastPfu Fly DNA Polymerase (TransGen Biotech, Beijing, China), TransStart FastPfu DNA Polymerase (TransGen Biotech), KOD-Plus-Neo (TOYOBO, Osaka, Japan), PrimeSTAR Max DNA polymerase (TaKaRa, Shiga, Japan), and 2 × Es Taq MasterMix (Dye) (CoWin Biosciences, Taizhou, China) were used for gene cloning. All endonucleases used in this study were purchased from Thermo Fisher Scientific (Waltham, MA, USA).

### Strains and cultivation conditions

Chemically competent *E. coli* DB3.1 and DH5α cells were used as the cloning hosts. DB3.1 cells were used for the construction of plasmids containing the *ccdB* expression cassette, while DH5α cells were used for the construction of the other plasmids (Supplementary Data 4). Chemically competent DB3.1 and DH5α cells were prepared according to the manufacturers’ instructions (Competent Cell Preparation Kit, TaKaRa or Z-Competent *E. coli* Transformation Kit, Zymo Research, Irvine, CA, USA). *E. coli* strains were grown in Luria-Bertani (LB) media containing 50 mg/L kanamycin or 100 mg/L ampicillin at 37 °C and 220 or 850 rpm.

*S. cerevisiae* YL-T3 and its derivatives were grown at 30 °C in either SC-H, SC-HL, SC-HLU, SC-HT, SC-HTL, or SC-HTLU as appropriate^68^ unless otherwise noted, or in yeast extract–peptone–dextrose (YPD) medium containing 40 g/L of glycerol with appropriate concentrations of G418 and hygromycin^31^ when conducting fermentation experiments. Yeast strains were grown in either 96-well plates (3960; Corning Inc., Corning, NY, USA) or 24-well plates (BIO-YD, Chengdu, China) at 850 rpm with 90% humidity, or in shake flasks at 220 rpm.

*G. lucidum* strain CGMCC 5.616 from the China General Microbiological Culture Collection Center (Beijing, China) was maintained on potato dextrose agar. Shake-static fermentation was performed at 30 °C^46,69^.

### Genome sequencing

The mycelia of *G. lucidum* 5.616 were collected after 2, 6, 8, and 10 days of shake-static fermentation, washed three times with distilled water, snap frozen in liquid nitrogen, and kept on dry ice while in transit to Tianjin Novogene Bioinformatic Technology Co., Ltd (Tianjin, China), where genomic DNA extraction and sequencing were performed. Genomic DNA was extracted^70^ and sequenced using the NovaSeq PE150 platform (Illumina, San Diego, CA, USA). The resulting 4.32 Gb of raw sequence data were filtered to obtain valid data (3.56 Gb of clean data). Another round of sequencing was performed using the PacBio Sequel platform (Pacific Biosciences, Menlo Park, CA, USA). Genome assembly was performed using SMRT Link v5.0.1 (Pacific Biosciences, Menlo Park, CA, USA) with optimized parameters. Illumina reads were then used to reduce the degree of heterozygosity using Purge Haplotigs software (https://bitbucket.org/mroachawri/purge_haplotigs/src/master/). RagTag software (https://github.com/malonge/RagTag) was then used for reference-guided scaffolding. Finally, Illumina reads were used again for error correction using Pilon software (https://github.com/broadinstitute/pilon). RNA sequencing and protein data from *G. lucidum* were then subjected to accurate gene structure annotation by Braker2 (https://anaconda.org/bioconda/braker2). All genes were then annotated based on 26 different databases and five tools covering information on the family, domain, classification, structure, gene ontology, and pathways using in-house scripts.

### cDNA synthesis and RNA sequencing

The mycelia of *G. lucidum* were collected at 2, 8, and 12 days after fermentation and frozen in liquid nitrogen for cDNA synthesis and RNA sequencing. For cDNA synthesis, the frozen mycelia samples were ground into a fine powder with a mortar and pestle, under liquid nitrogen. One mL of TRIzol (Invitrogen, Carlsbad, CA, USA) was used to extract total RNA from 100 mg fresh samples. The isolated RNA was treated with RNase-free DNase I (Thermo Fisher Scientific) to remove residual genomic DNA. RNA concentration and quality were determined using a NanoDrop2000^TM^ spectrophotometer (Thermo Fisher Scientific) and agarose gel electrophoresis. cDNA was synthesized from the isolated RNA using the RevertAid^TM^ First Strand cDNA Synthesis Kit (Thermo Fisher Scientific) according to the manufacturer’s instructions. For RNA sequencing, the frozen mycelia samples were sent to Tianjin Novogene Bioinformatic Technology Co., Ltd for RNA isolation, library preparation, and transcriptome sequencing^71^. The raw data (raw reads), in FASTQ format, were first processed using in-house Perl scripts. Clean data (clean reads) were obtained by removing reads containing adapter sequences and N bases and low-quality reads from the raw data. All downstream analyses were performed on clean high-quality data. The clean reads were then aligned to the genome assembly using Hisat2 (https://daehwankimlab.github.io/hisat2/download/), and Stringtie2 (http://ccb.jhu.edu/software/stringtie/index.shtml) was used for fragments per kilobase of sequence per million mapped reads (FPKM) calculations. The FPKM value of each transcript was calculated based on the length of the transcript and number of reads mapped to the transcript.

### High-throughput DNA manipulation

Primers were synthesized by Tsingke Biotechnology (Beijing, China) and the Tianjin Institute of Industrial Biotechnology, Chinese Academy of Sciences (Tianjin, China). A TIANgel Midi Purification Kit (TIANGEN, Beijing, China) was used for DNA purification. The plasmids were constructed using a ClonExpress MultiS One Step Cloning Kit or ClonExpress II One Step Cloning Kit (Vazyme, Nanjing, China). A TIANprep Mini Plasmid Kit (TIANGEN) was used for plasmid isolation. The strains, plasmids, and primers used in this study were listed in Supplementary Data 3 and 4.

To facilitate CYP expression plasmid construction, we first constructed a *ccdB*-harboring plasmid, pRS426-ccdB-G418r. To construct CYP expression plasmids, we attempted to amplify all 215 CYP candidates from *G. lucidum* cDNA and ligated them with the *Sap*I-linearized vector pRS426-ccdB-G418r in 96-well plates (PCR-96-FS-C; AXYGEN, Union City, CA, USA), resulting in the plasmid pRS426-CYP(s)-G418r. In detail, the primers used to amplify the CYP genes were arranged in 96-well plates (PCR-96-FS-C). The PCR premix, containing all of the components required for amplification, except the primers, was prepared according to the manufacturer’s instructions, and 10 μL was added to each well of the 96-well plates using the Biomek i7 Automated Workstation (Beckman Coulter, Inc., Brea, CA, USA). The primers were added according to the manufacturer’s instructions. After mixing, PCR was performed using a thermocycler (PowerCycler; Biometra, Göttingen, Germany). Then, 1 μL of unpurified PCR product was added to 4 μL of the ClonExpress II One Step Cloning Kit ligation premix containing the *Sap*I-linearized plasmid pRS426-ccdB-G418r in 96-well plates, to ligate the two fragments. Ligation was performed in a thermocycler at 37 °C for 30 min, and the ligated samples were then stored at 4 °C. Five microliters of the ligation products were added to 50 μL of chemically competent DH5α cells prepared using the Z-Competent *E. coli* Transformation Kit, according to the manufacturer’s instructions. The samples were incubated in a thermocycler at 4 °C for 5 min, and then 50 μL of the transformation products were spread on 48-well Q Tray Vented plates (XGE05099; JARDEN, Christchurch, UK) containing solid LB medium (with 100 mg/L ampicillin). After incubation at 37 °C overnight, two parallel colonies of each transformation were picked and inoculated into 96-well plates (3960, Corning Inc.), in which each well contained 1 mL of LB medium supplemented with 100 mg/mL ampicillin. After culturing overnight, the plasmids were extracted using an EZgene™ Plasmid Miniprep Kit (Beads) (BIOMIGA, Hangzhou, China) according to the manufacturer’s instructions. In the above procedures, transformed cell spreading on 48-well Q Tray Vented plates, colony picking, and inoculation in 96-well plates were performed using a ClonePix system (Genetix QP Expression, New Milton, UK). Other operations involving 96-well plates, including plasmid extraction procedures, were performed using a Biomek i7 Automated Workstation. The extracted plasmids were sent to Tsingke Biotechnology for sequencing analysis to verify the CYP sequence, using the primers P450-CX-F and P450-CX-R. The successfully cloned plasmids and strains were rearranged in new 96-well microplates (AXYGEN, PCR-96-FS-C) and 96-well deep-well plates (Corning Inc., 3960), respectively. For CYPs that were not obtained in the first round, the corresponding primers were rearranged to perform a second round of cloning procedures using different conditions, such as different annealing temperatures and DNA polymerases.

### High-throughput yeast transformation and fermentation

To obtain yeast strains expressing CYPs, chemically competent yeast cells were prepared and transformed in 96-well plates (651101; Greiner Bio-One, Frickenhausen, Germany) using the Frozen-EZ Yeast Transformation II Kit, according to the manufacturer’s instructions (Zymo Research), with the aid of the Biomek i7 Automated Workstation. Forty microliters of each transformation product were then spread on 48-well Q Tray Vented plates with an appropriate solid medium (SC medium without different amino acids). After incubation at 30 °C for 2–3 days, two parallel colonies (unless noted otherwise) of each transformant were picked and inoculated into 24-well plates, in which each well contained 3 mL of the appropriate medium. After incubating for 2 days, the cultures were inoculated into 3 mL of the same medium in 24-well plates at 3% and then incubated for 14 h. Three percent of the resulting seed cultures was inoculated into 3 mL of YPD medium containing 40 g/L glycerol and the appropriate concentrations of G418 and hygromycin in 24-well plates to conduct fermentation for 5 days. The yeast transformation, fermentation culture preparation, and inoculation in 24-well plates were performed using the Biomek i7 Automated Workstation. Transformant spreading and colony picking were performed using the ClonePix system.

### High-throughput product extraction in plates

Product extraction was performed partially using the Biomek i7 Automated Workstation. In detail, after centrifugation at 8000 × *g* for 10 min, the plates containing the cultures were moved to the Biomek i7 Automated Workstation to perform the following product extraction procedures using a 96-channel pipette coupled with an automated arm. The supernatant was discarded, 2 mL of methanol was added to each well, and samples were immediately mixed. The 24-well plates were sealed and then incubated at 30 °C and 850 rpm for 1 h in a shaker. After centrifugation at 8000 × *g* for 10 min, the supernatant was manually filtered through a 0.22-μm nylon syringe filter (JIN TENG, Tianjin, China) and then subjected to HPLC analysis. Finally, UPLC-MS analysis was performed on the samples of interest.

### Large-scale fermentation, extraction, and purification of compounds

To obtain **1**, a single clone of strain CYP5150W17-r-iGLCPR-r was picked from the SC-HLU solid medium plate, inoculated into 20 mL of SC-HLU liquid medium and cultivated to an OD_600_ of 2–2.5. The preculture was then inoculated into 300 mL of SC-HLU and grown to an OD_600_ of 2–2.5. The resultant seed culture was inoculated into 9.6 L of fermentation medium. The fermentation was performed in 2-L shake flasks, with each flask containing 400 mL of fermentation medium. After 5 days of fermentation, 400 mL of ethyl acetate was added to each shake flask, and the cultures were incubated at 30 °C and 220 rpm for 1 h to extract the intracellular products. After centrifugation, the ethyl acetate layer was collected. The ethyl acetate extraction procedure was performed twice, and the collected samples were combined and concentrated in vacuo. The concentrated extracts were then re-dissolved in less than 20 mL of methanol and used for the subsequent purification of **1**. Strain CYPFUM15A2-r-CYP5150L8-iGLCPR-r was precultured in SC-HLU and fermented in YPD medium containing 40 g/L glycerol, 500 mg/L G418, and 300 mg/L hygromycin for the purification of **2**. Strain CYP512W2-r-CYP5150L8-iGLCPR-r was precultured in SC-HLU and fermented in YPD medium containing 40 g/L glycerol, 500 mg/L G418, and 300 mg/L hygromycin for the purification of **3**, **4**, **5**, **6**, and **7**.

To obtain pure **1**, **2**, **3**, **5**, **6**, and **7**, the crude extracts were purified on a preparative Agilent 1200 LC system (Agilent, Waldbronn, Germany). Solvent A was ultrapure water and solvent B was acetonitrile. In the first round of purification, a Kromasil100-10-C18 column (20 × 250 mm) was used to purify **1**, **2**, and **3**, while an Elite 100-c-18 (20 mm × 250 mm) was used to purify **5**, **6**, and **7**. The samples were eluted from the columns with a linear gradient of 80–100% B for 30 min and 100% B for 10 min at a flow rate of 10 mL/min. In the second round of purification, **1** and **2** were purified using an Elite Hypersil ODS2 column (10 × 250 mm) and were eluted with a linear gradient of 80-100% B for 30 min and 100% B for 10 min at a flow rate of 2 mL/min. **3** was purified using an Elite Hypersil ODS2 column (10 × 250 mm) and was eluted with a linear gradient of 80–100% B for 40 min and 100% B for 10 min at a flow rate of 1 mL/min. **5** was purified using a Kromasil C18 column (21 mm × 250 mm) and was eluted with a linear gradient of 80–100% B for 30 min and 100% B for 10 min at a flow rate of 10 mL/min. **6** and **7** were purified using an Elite ODS-C18 column (10 mm × 250 mm) and were eluted with a linear gradient of 50–70% B for 60 min at a flow rate of 2 mL/min. In the third round of purification, **5** and **6** were purified using a Kromasil C18 column (21 mm × 250 mm) and were eluted with a linear gradient of 60-100% B for 60 min and 100% B for 10 min at a flow rate of 10 mL/min.

To purify **4**, crude extracts were delivered to a silica chromatography column (40 [34] × 500 mm) and sequentially eluted with a petroleum ether–ethyl acetic system. Fractions containing **4** were further purified using an Agilent 1260 semipreparative HPLC system equipped with a multiple wavelength detector at 214 nm and an ODS-A column (20 × 250 mm, s-5 µm, 12 nm; Waters, Milford, USA). Mobile phase B contained methanol/formic acid (1000:1), and mobile phase A was 100% water. The column was eluted at a flow rate of 10 mL/min sequentially with a linear gradient of 80–100% B for 50 min, 100% B for 10 min, 100–80% B for 0.5 min, and 80% B for 9.5 min. Finally, the eluate with pure target compound was combined and concentrated in vacuo to a dry powder.

### In vitro enzymatic reactions

Yeast strain CYP512W2-r-iGLCPR-r and the control strain CK-r-iGLCPR-r were used for microsome isolation. Yeast microsome preparation and enzymatic assays were performed with minor modifications to the RT and substrate concentration^28^. GA-HLDOA, **3**, or **5** (400 μM) were used as the substrates in the required reactions. For enzyme inactivation, reaction mixtures underwent heat inactivation at 80 °C for 10 min prior to substrate addition. The CYP512W2-containing microsomes were incubated with GA-HLDOA for 1, 3, or 18 h. In another experiment, the CYP512W2-containing microsomes were incubated with GA-HLDOA for 1 h, heat inactivated, and then incubated with GA-HLDOA for another 2 h. For the other in vitro enzymatic assays, the RT was 18 h. After the reaction was completed, the products were extracted with ethyl acetate and subjected to UPLC-MS analysis.

### Yeast chromosome integration

To integrate the CYP5150L8 and iGLCPR expression cassettes at rDNA loci by directly using a homologous recombination donor, 0.1 pmol of the donor rDNA1-eGFP-TRP1-CYP5150L8-iGLCPR-rDNA2, which was obtained from plasmid pET28a-rDNA1-eGFP-TRP1-CYP5150L8-iGLCPR-rDNA2 by *Sap*I digestion, was introduced into YL-T3. SC-HT solid medium plates were used for transformant selection. After incubation for 2 days, several colonies were randomly picked for analysis and genotype confirmation.

### Fluorescence-activated cell sorting

The direct transfer of the donor rDNA1-eGFP-TRP1-CYP5150L8-iGLCPR-rDNA2 into YL-T3 resulted in more than 5000 transformants. These transformants were mixed, re-suspended in 3 mL of sterile water, and inoculated at a volume:volume ratio of 1% into 4 mL of SC-HT liquid medium in a 16-mL glass tube. The transformants were cultivated at 30 °C and 220 rpm to an OD_600_ of 1.5–2.5. The cultures were inoculated into 10 mL of SC-HT liquid medium in a 250-mL shake flask at an initial OD_600_ of 0.2 and cultivated for several hours to an OD_600_ of 1.5-2.5. The cells were harvested by centrifugation (10,000 × *g*, 30 s), washed once with phosphate-buffered saline (PBS, pH 7.4), and re-suspended in PBS (pH 7.4) to an OD_600_ of 0.1–0.2. The cells were further dispersed using an ultrasonic cleaner (SB120D; SCIENTZ, Ningbo, China) for 2 min. Ten milliliters of the resulting cell suspension was then used for FACS analysis (MoFlo XDP High-Speed Sorter, Beckman Coulter) to isolate GFP-positive cells^72^. Yeast cell gating strategy followed: FSC (voltage 180 V), SSC (voltage 400 V), and the threshold was set as 3% (triggered on FSC channel). All captured events were used for fluorescence analysis. GFP fluorescence was analyzed on FL1 channel (voltage 400 V, excitation at 488 nm, emission fluorescence at 529 ± 14 nm). Cells with the top 0.01% fluorescence signal were collected (Supplementary Fig. 50). Finally, 960 cells with high fluorescence signal intensity were isolated and individually inoculated in 1 mL of SC-HT liquid medium in 96-well plates. After cultivating for 3 days, 3% of the culture sample was inoculated in 3 mL of SC-HT medium in 24-well plates and cultivated for 1.5 days. Three percent of the culture sample was then inoculated in 3 mL of SC-HT medium in 24-well plates and cultivated for 12 h. A 0.2 mL sample of each culture was transferred to a 96-well microplate with a transparent bottom (Costar 3603, Corning Inc.) to determine the OD_600_ and fluorescence intensity. Strains with a fluorescence signal intensity higher than 9000 were picked for the following fermentation experiments.

### Fermentation of strain CYP512W2-r-CYP5150L8-iGLCPR-r in a shake flask

Four colonies of strain CYP512W2-r-CYP5150L8-iGLCPR-r on an SC-HLU solid medium plate were individually inoculated into 250-mL shake flasks containing 10 mL of SC-HLU and cultivated to an OD_600_ of 1.5–2.5. The cells were then sub-cultured into 500-mL shake flasks containing 20 mL of SC-HLU with an initial OD_600_ of 0.05 and grew to an OD_600_ of 2–2.5. The resultant cultures were inoculated into 500-mL shake flasks containing 50 mL of YPD medium supplemented with 40 g/L of glycerol, 500 mg/L of G418, and 300 mg/L of hygromycin with an initial OD_600_ of 0.05. Samples were taken every day during the 6 days of fermentation. The results were presented as the means ± standard deviation of four independent samples.

### Analytical methods

Cell growth was determined by measuring the OD_600_ using a V-1000 spectrophotometer (AOE, Shanghai, China) or a microplate reader (Neo2; BioTek, Winooski, VT, USA) when 96-well microplates with transparent bottoms were used. The fluorescence signal was detected at an excitation wavelength of 470 ± 4.5 nm and an emission wavelength of 515 ± 4.5 nm. Glucose, glycerol, ethanol, and acetate acid concentrations were determined by an Agilent 1200 HPLC system (Agilent, Waldbronn, Germany) equipped with a refractive index detector and an Aminex HPX-87H column (300 mm × 7.8 mm, BIO-RAD, California)^31^. Metabolites, including squalene, 2,3-oxidosqualene, lanosterol, GA-HLDOA, **1**, and the derivatives of GA-HLDOA were analyzed by HPLC with minor modifications to the elution method^31^. To analyze the fermentation extracts during the in vivo screening of CYPs in the lanosterol-producing strain, samples were eluted from the column with a linear gradient of 85–100% B for 30 min, 100% B for 5 min, 100–85% B for 1 min, and 85% B for 9 min. To analyze fermentation extracts during the in vivo screening of CYPs in the GA-HLDOA-producing strain, the fermentation of strains obtained after FACS, and the fermentation of strain CYP512W2-r-CYP5150L8-iGLCPR-r in a shake flask, the samples were eluted from the column with a linear gradient of 80–100% B for 30 min, 100% B for 5 min, 100–80% B for 1 min, and 80% B for 9 min. To analyze the fermentation extracts during the in vivo screening of CYPs in the **3**- and **4**-producing strain, the samples were eluted from the column with a linear gradient of 66%-100% B for 30 min, 100% B for 5 min, 100–66% B for 1 min, and 66% B for 9 min. UPLC-MS was performed by using an UPLC (Waters, Wilmslow, UK), connected to a Q-TOF MS (Waters, Wilmslow, UK) in atmospheric pressure chemical ionization mode, and equipped with a Waters BEH C18 column (1.7 µm, 2.1 × 100 mm, Waters, UK)^66^, unless otherwise noted. High-resolution mass spectrometry was performed, and the corresponding delta ppm values of the detected *m/z* are shown in Supplementary Table 1.

The NMR spectra of the purified compounds were measured on an Avance III 600 MHz Nuclear Magnetic Resonance instrument (Bruker, Karlsruhe, Germany) in CDCl_3_ solvent, except for GA-Jb, which was analyzed in methanol-d4 solvent. Chemical shifts (*δ*) were expressed in ppm and coupling constants (*J*) were expressed in hertz (Hz). The NMR data were analyzed by using MestReNova (v 14.0.0).

### Statistics

All *P* values were generated from two-tailed *t*-tests using the OriginPro (v 9.8.0.200, OriginLab Corporation) software. The significance threshold was *P* < 0.05 for all experiments.

### Reporting summary

Further information on research design is available in the Nature Portfolio Reporting Summary linked to this article.

## Supplementary information

Supplementary Information

Description of Additional Supplementary Files

Supplementary Data 1

Supplementary Data 2

Supplementary Data 3

Supplementary Data 4

Supplementary Data 5

Reporting Summary

## Data Availability

The data supporting the findings of this work are available within the paper and the Supplementary Information files. A reporting summary for this article is available as a Supplementary Information file. The genome and transcriptome sequencing data are available at NCBI under accession PRJNA796760. The annotation data are available at GitHub [https://github.com/TIBseqlab/Galuci_5_616]. Source data are provided with this paper.

## Source data

Source Data
