# Supplementary material for: Biosynthesis of mushroom-derived type II ganoderic acids by engineered yeast
Source: Nat Commun. 2022 Dec 14;13:7740. doi: 10.1038/s41467-022-35500-1 (PMC9748899; doi:10.1038/s41467-022-35500-1)
Supplement: Supplementary file 1 — Supplementary Information [file 41467_2022_35500_MOESM1_ESM.pdf]

Biosynthesis of mushroom-derived type II ganoderic acids by engineered yeast

Yuan *et al.*

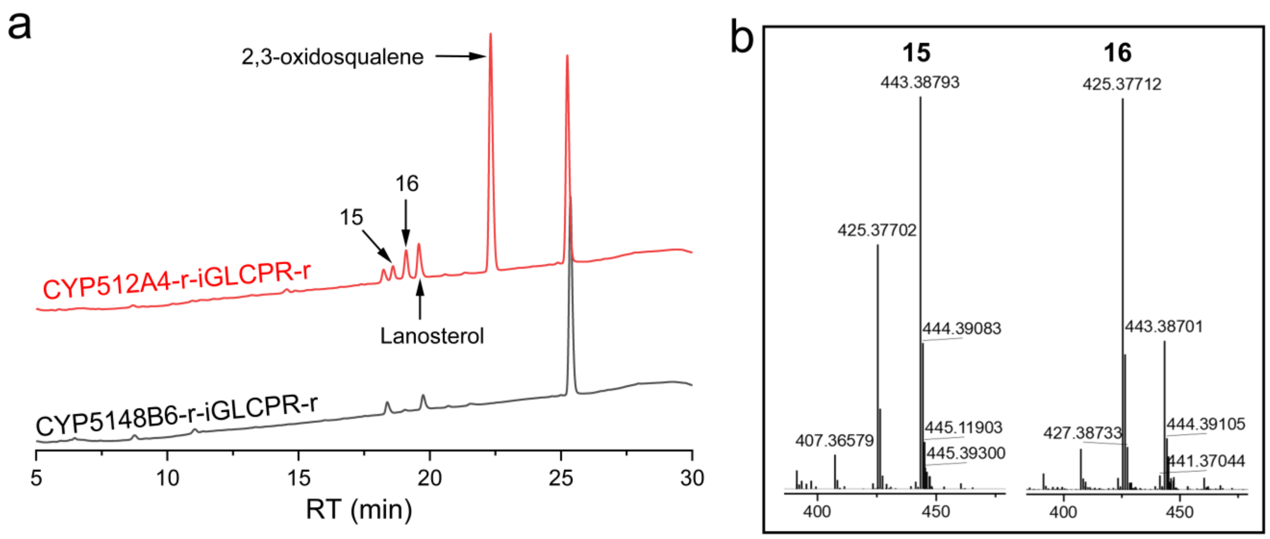

Supplementary Figure 1. Functional CYP screening in strains iGLCPR-r. a UPLC analysis of extracts of strain CYP512A4-r-iGLCPR-r (red line) and the control strain CYP5148B6-r-iGLCPR-r (black line); **b** MS spectra of **15** and **16** as indicated in a. Y-axis represents the total ion current (TIC) intensity. UPLC-MS analyses were conducted using an ultra-performance liquid chromatography (UPLC, LC-30A, Shimadzu) connected to a TripleTOF MS spectrometer (TripleTOF 6600, Sciex) in atmospheric pressure chemical ionization (APCI) mode. The mobile phase and the elution condition were the same as the method for HPLC analysis of the fermentation extracts during the *in vivo* screening of CYPs in the lanosterol-producing strain.

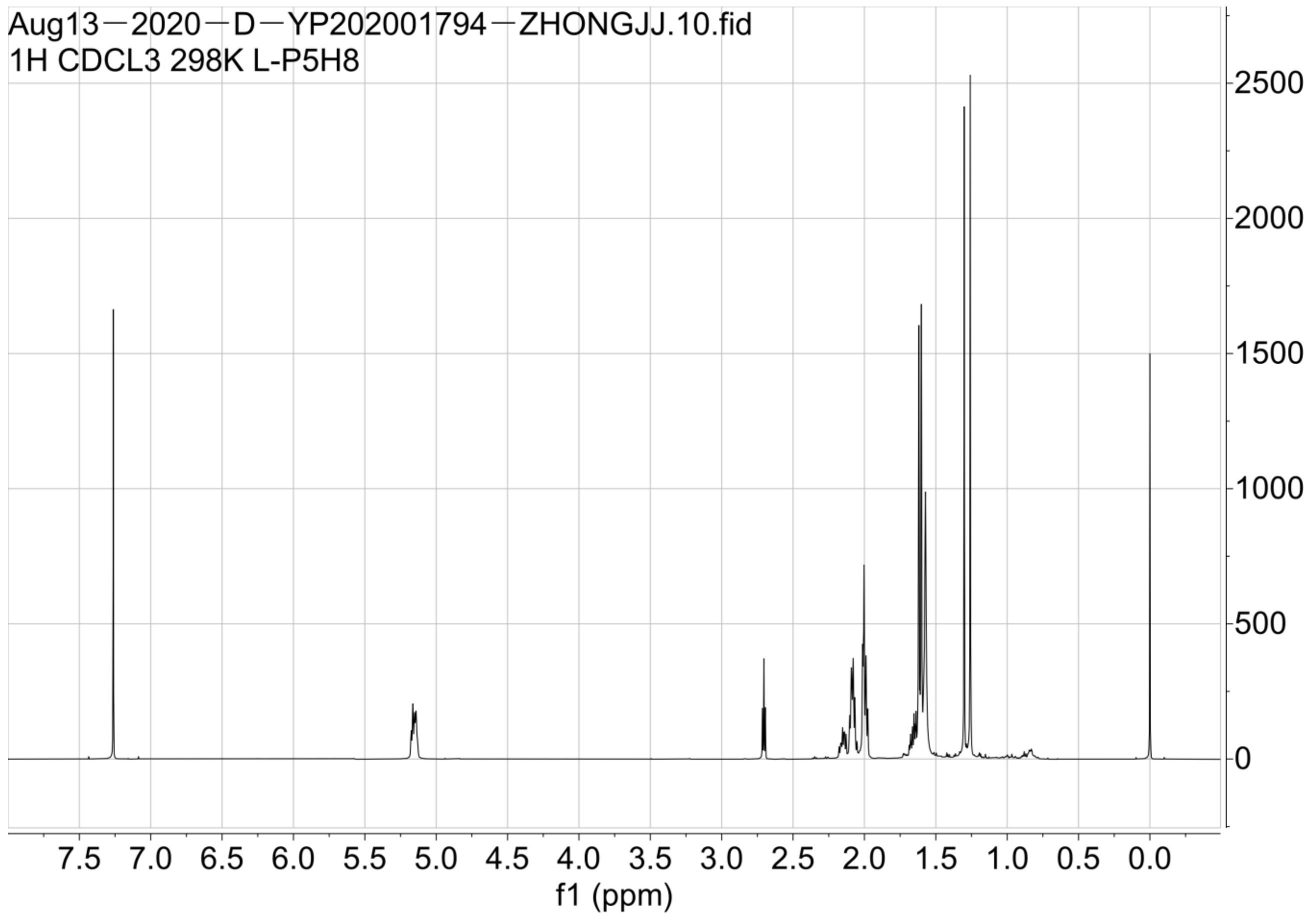

Supplementary Figure 2. ^{1}H NMR spectrum of 1.

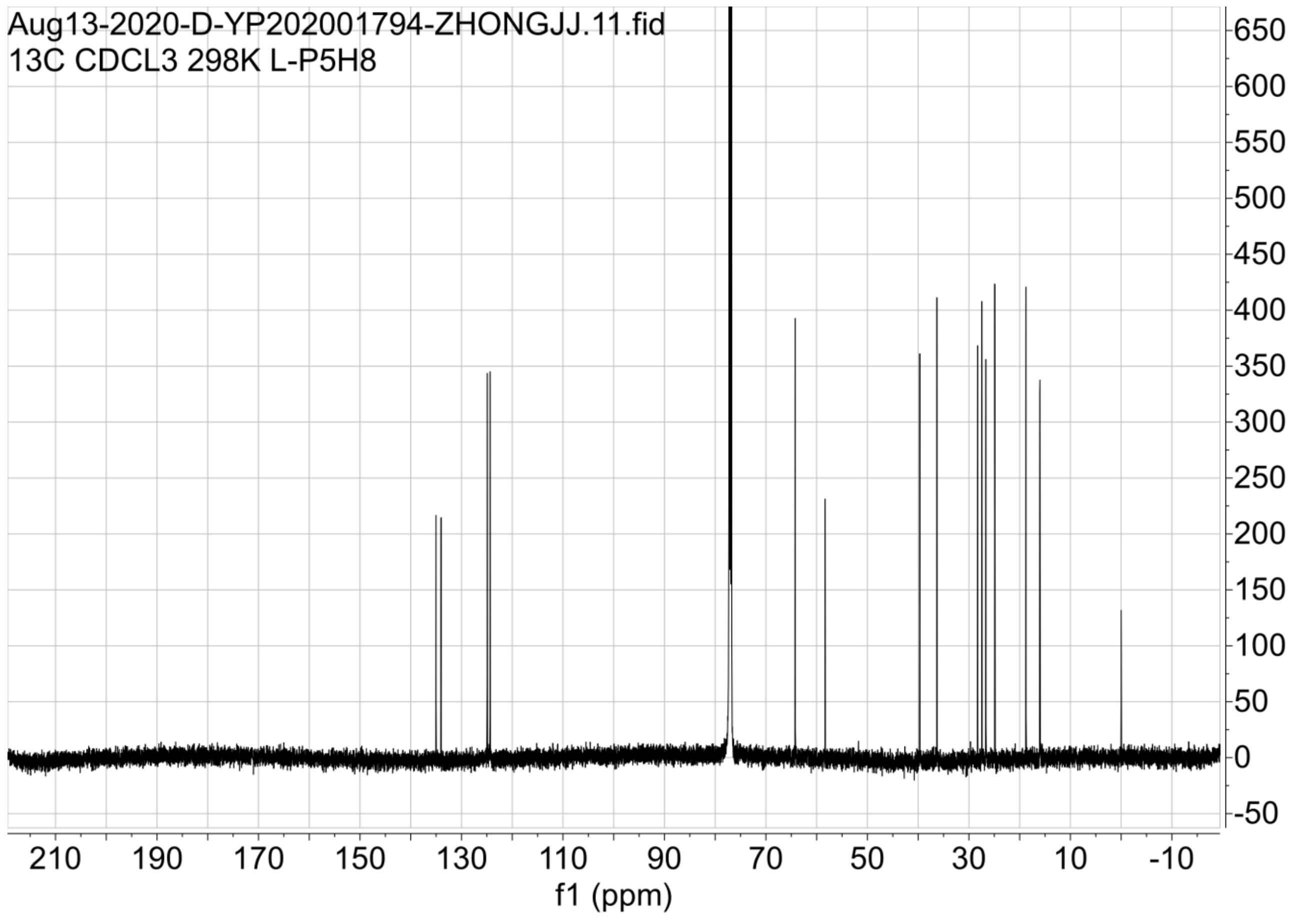

Supplementary Figure 3. ^{13}C NMR spectrum of 1.

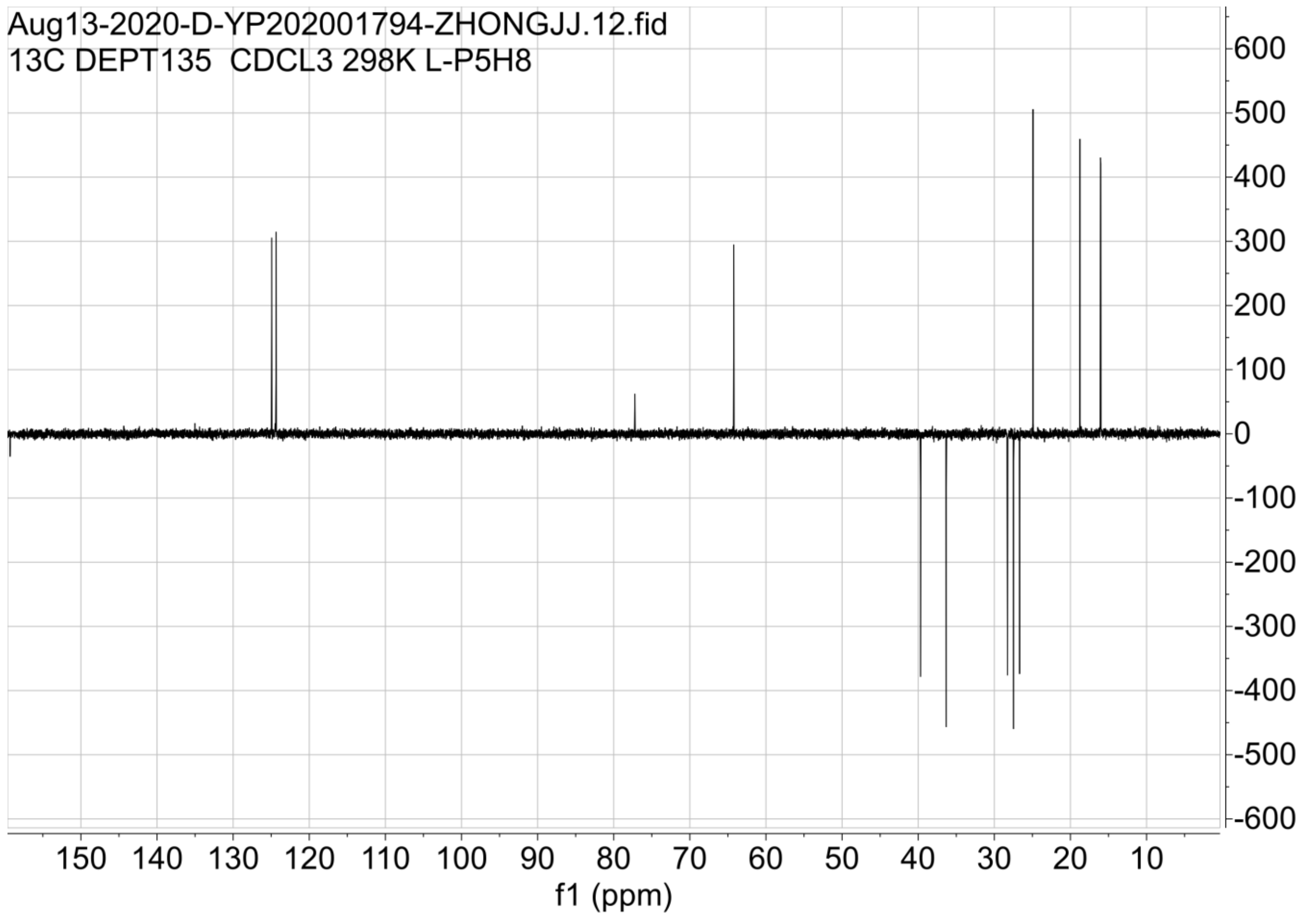

Supplementary Figure 4. DEPT-135 spectrum of 1.

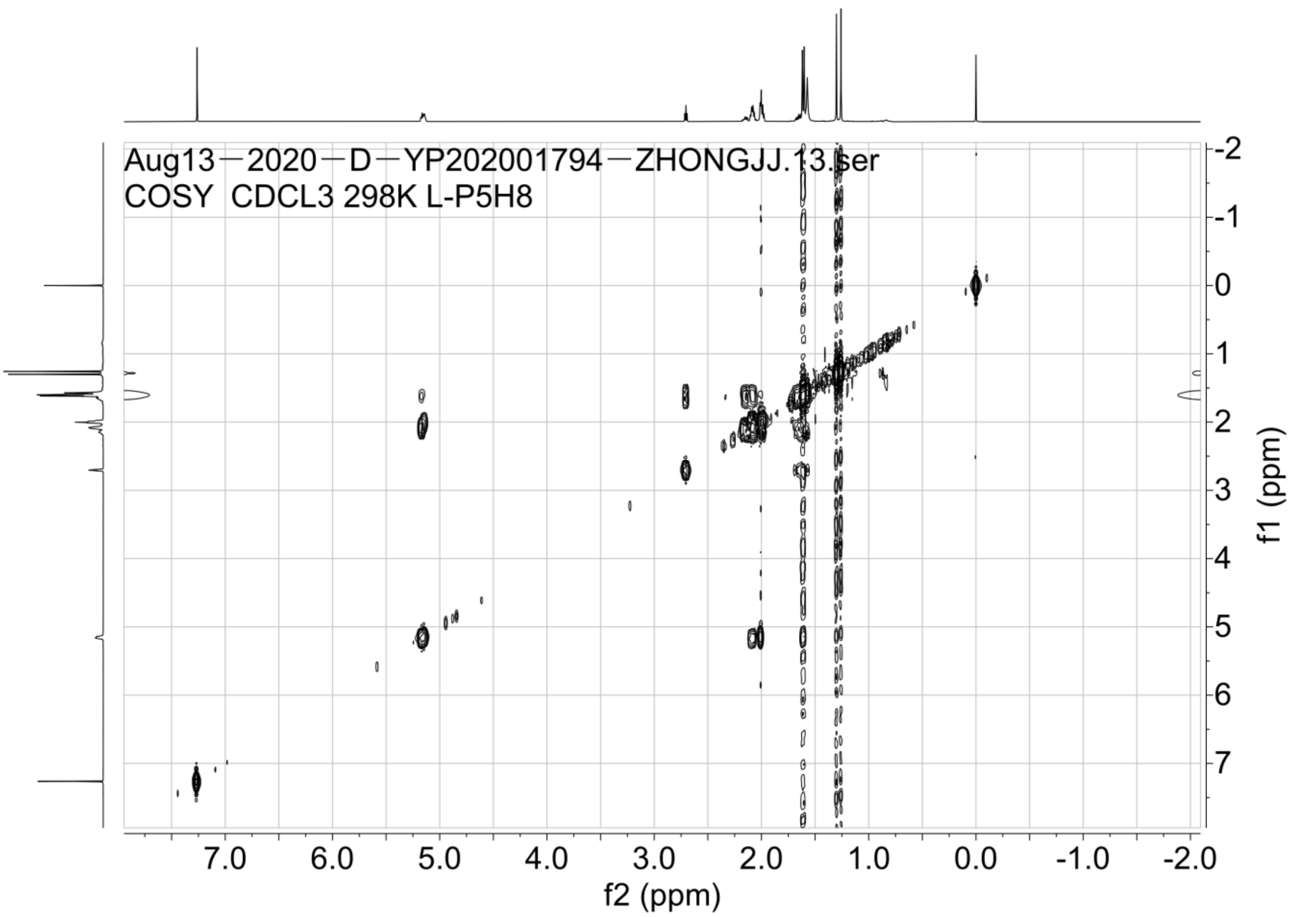

Supplementary Figure 5. COSY spectrum of 1.

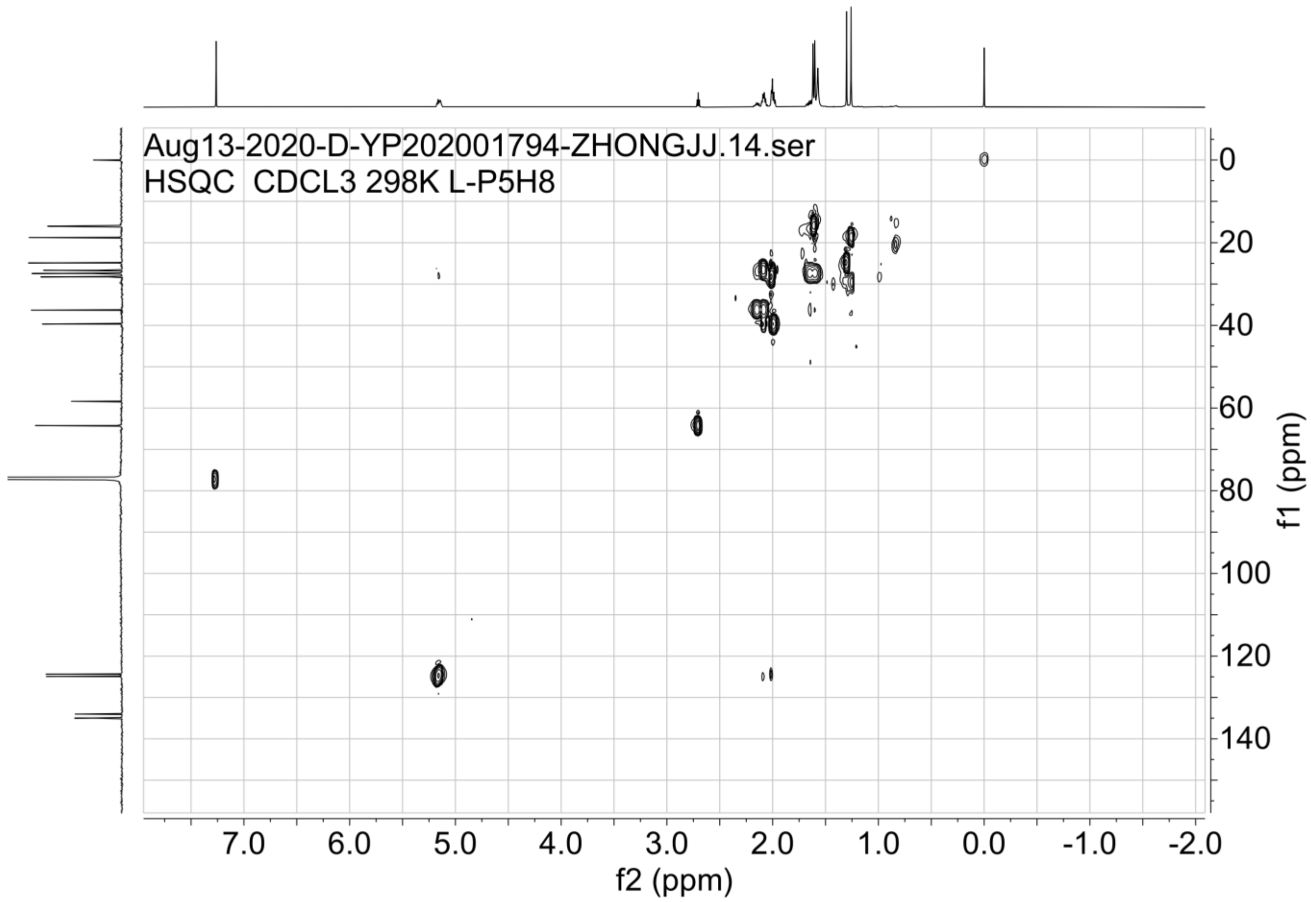

Supplementary Figure 6. HSQC spectrum of 1.

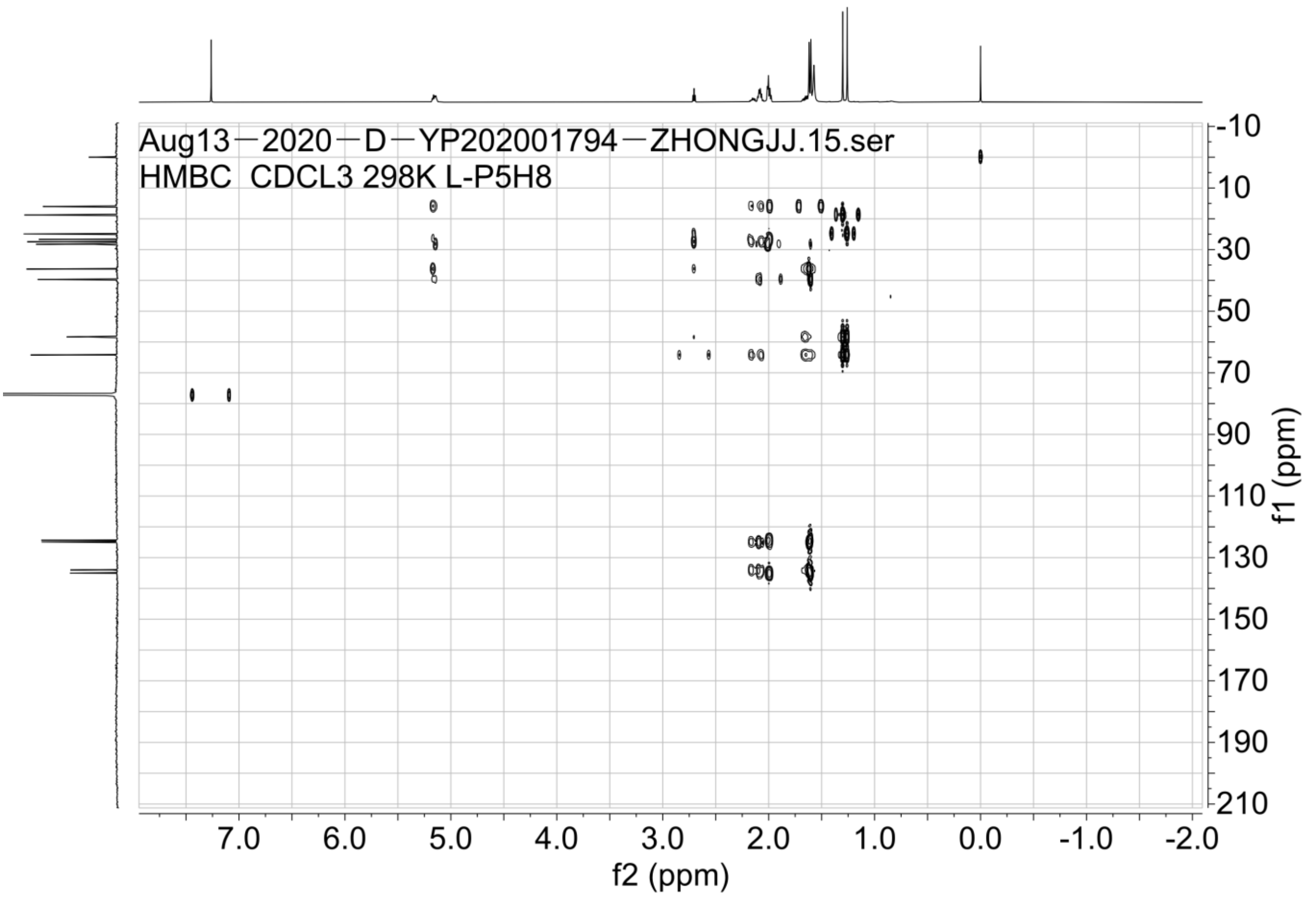

Supplementary Figure 7. HMBC spectrum of 1.

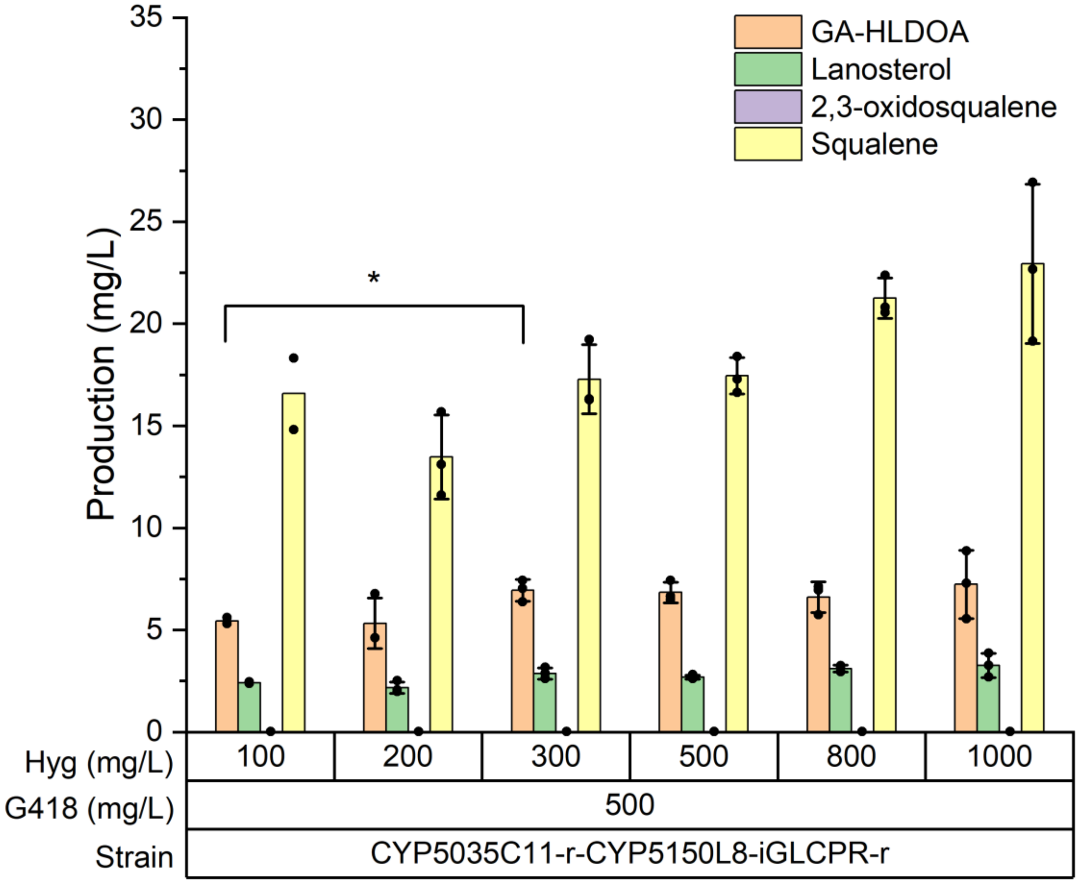

Supplementary Figure 8. Production of GA-HLDOA, lanosterol, 2,3-oxidosqualene, and squalene after 120 h fermentation of strain CYP5035C11-r-CYP5150L8-iGLCPR-r, under different concentrations of G418 and hygromycin. All data represent the mean of two (columns 1-4) or three (all the other columns) biologically independent samples (solid circles) and error bars show standard deviation. Student's two-tailed t-test: *P = 0.03579. Source data are provided as a Source Data file.

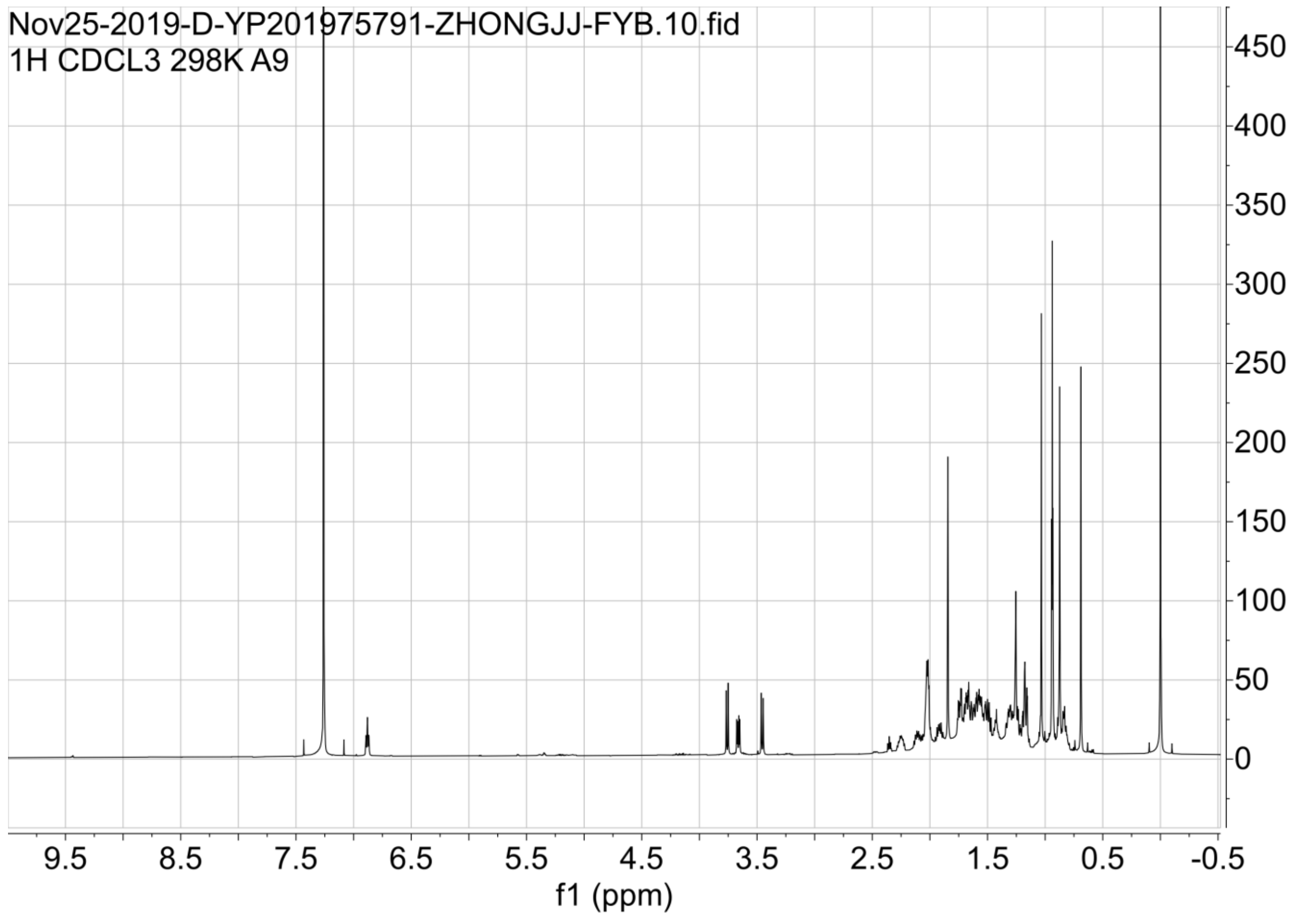

Supplementary Figure 9. ^{1}H NMR spectrum of 2.

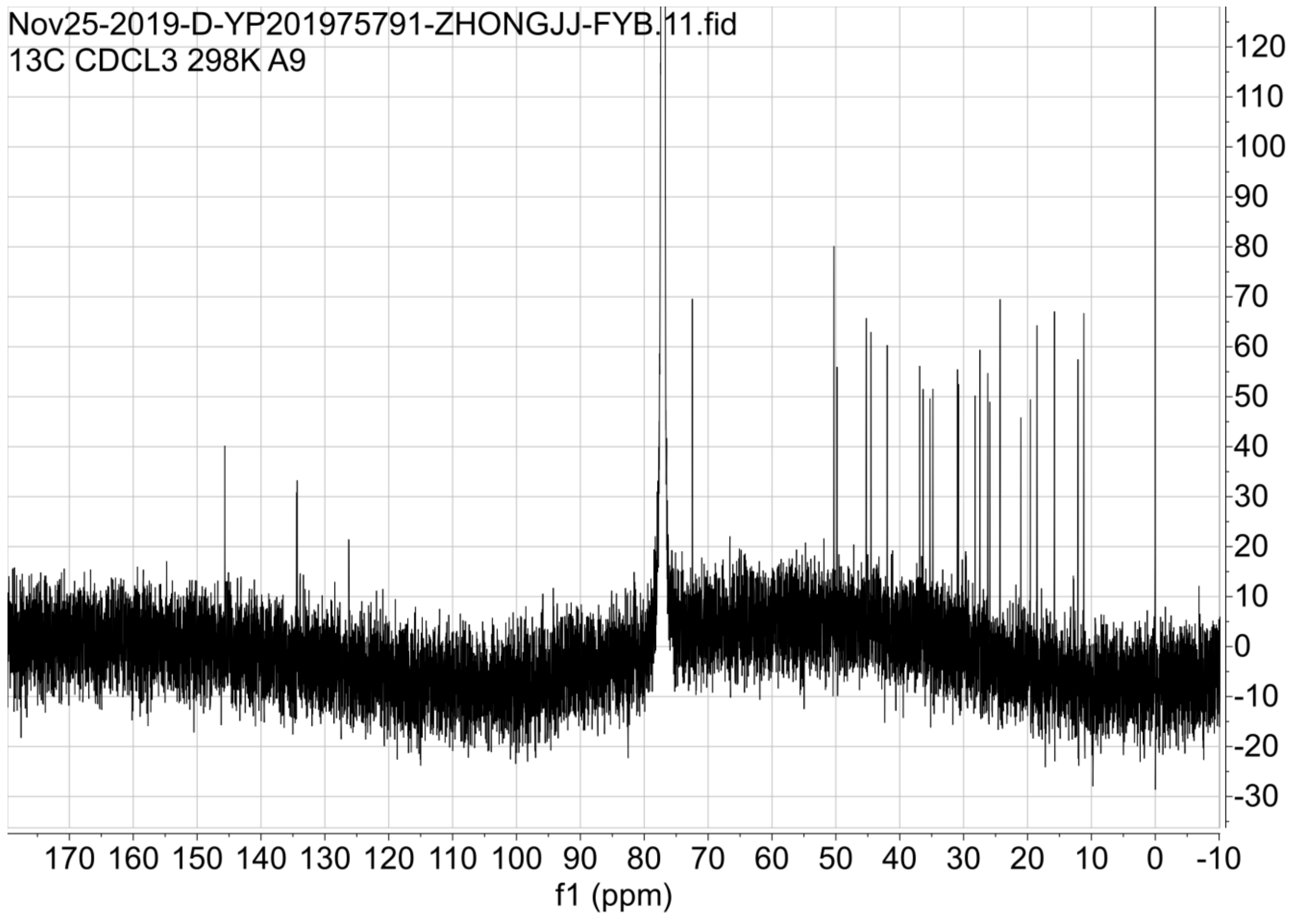

Supplementary Figure 10. ^{13}C NMR spectrum of 2.

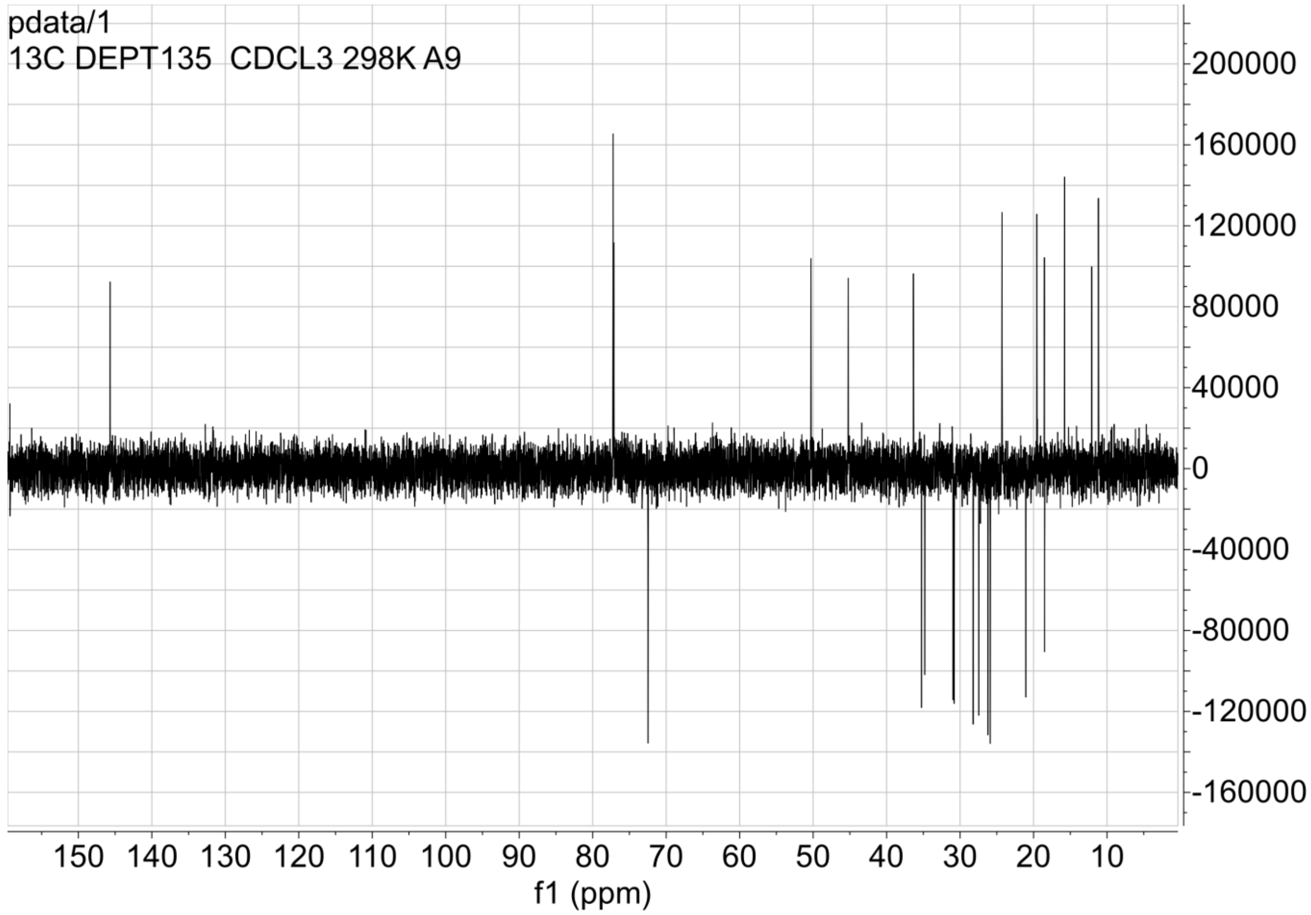

Supplementary Figure 11. DEPT-135 spectrum of 2.

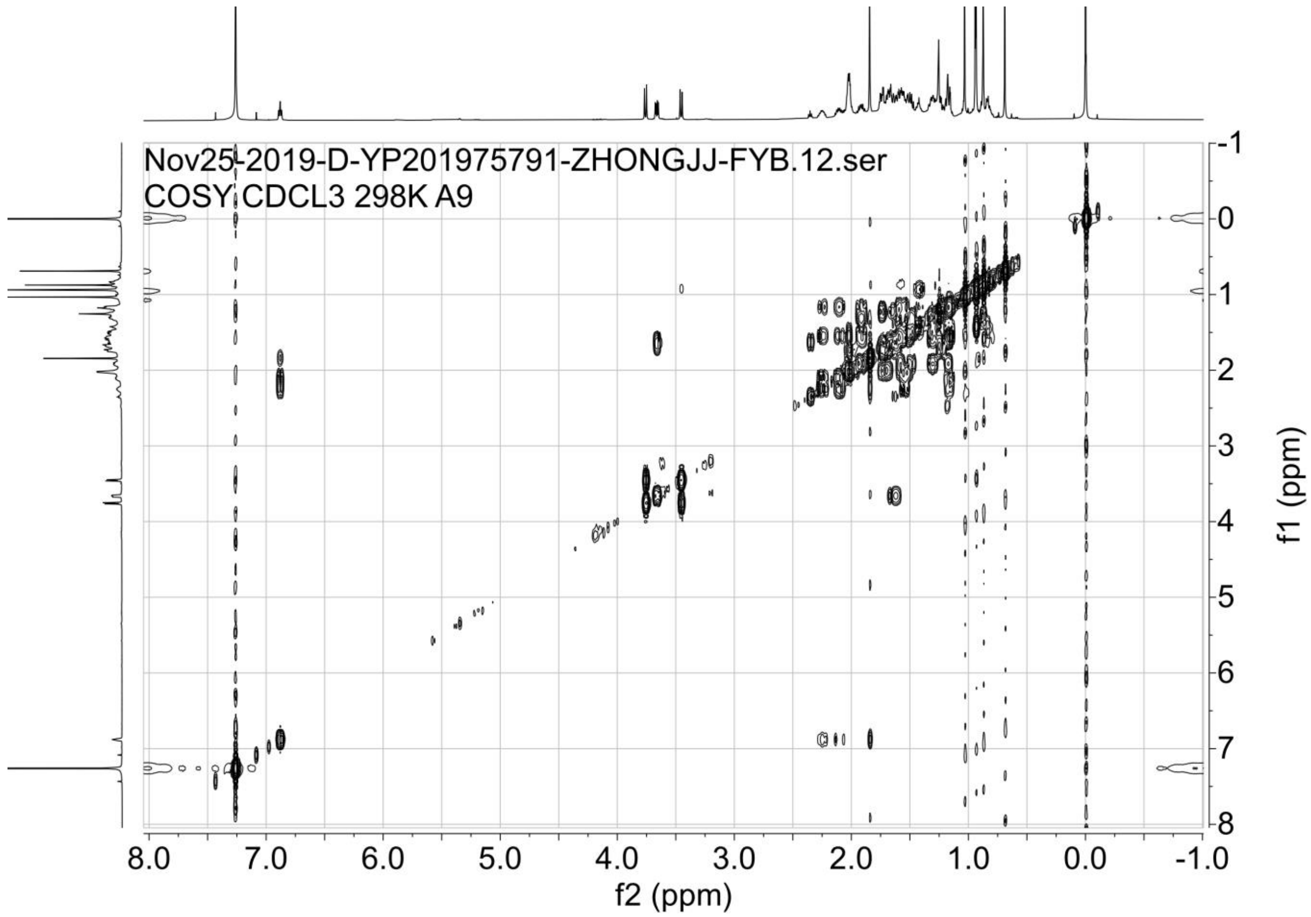

Supplementary Figure 12. COSY spectrum of 2.

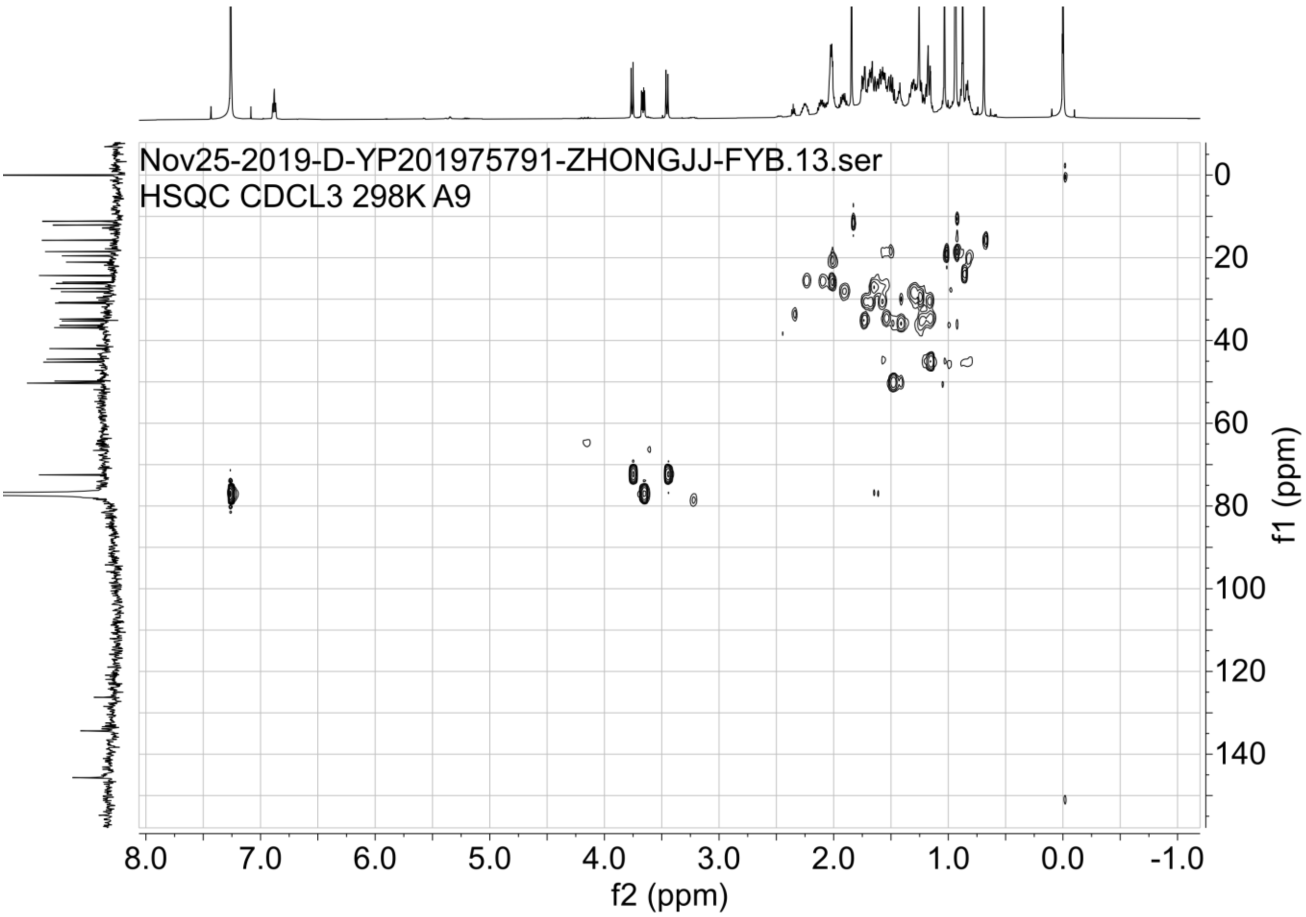

Supplementary Figure 13. HSQC spectrum of 2.

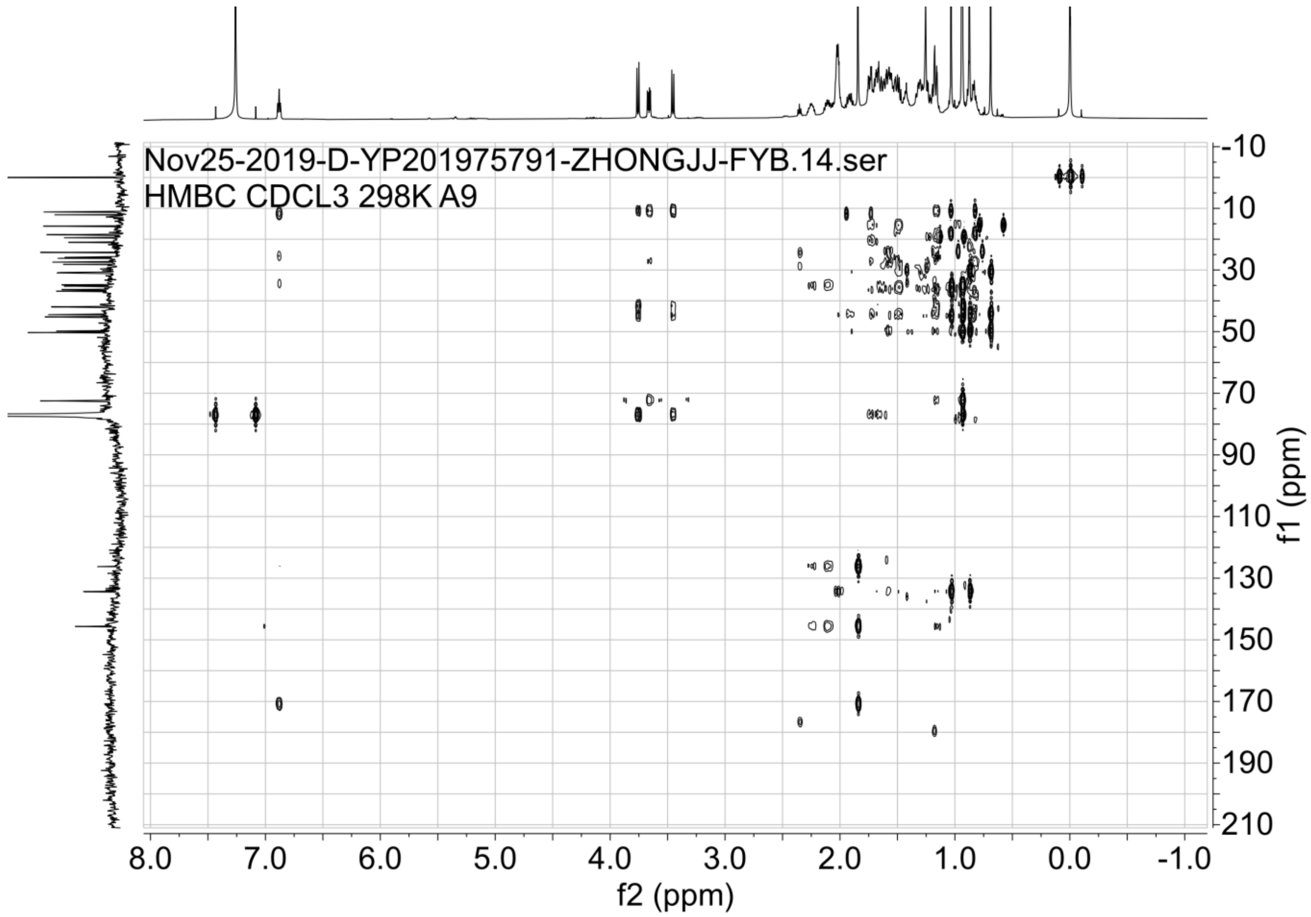

Supplementary Figure 14. HMBC spectrum of 2.

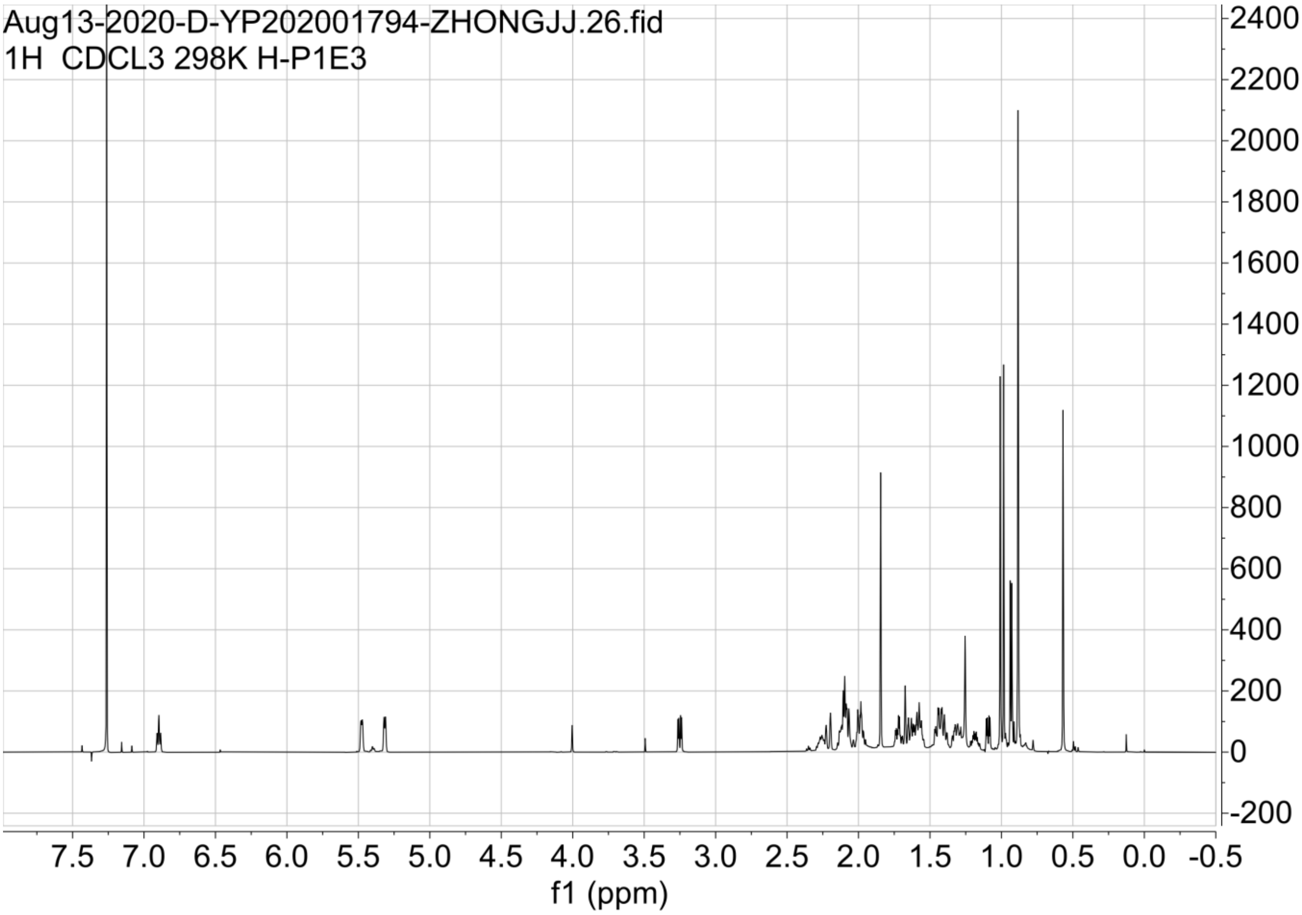

Supplementary Figure 15. ^{1}H NMR spectrum of 3.

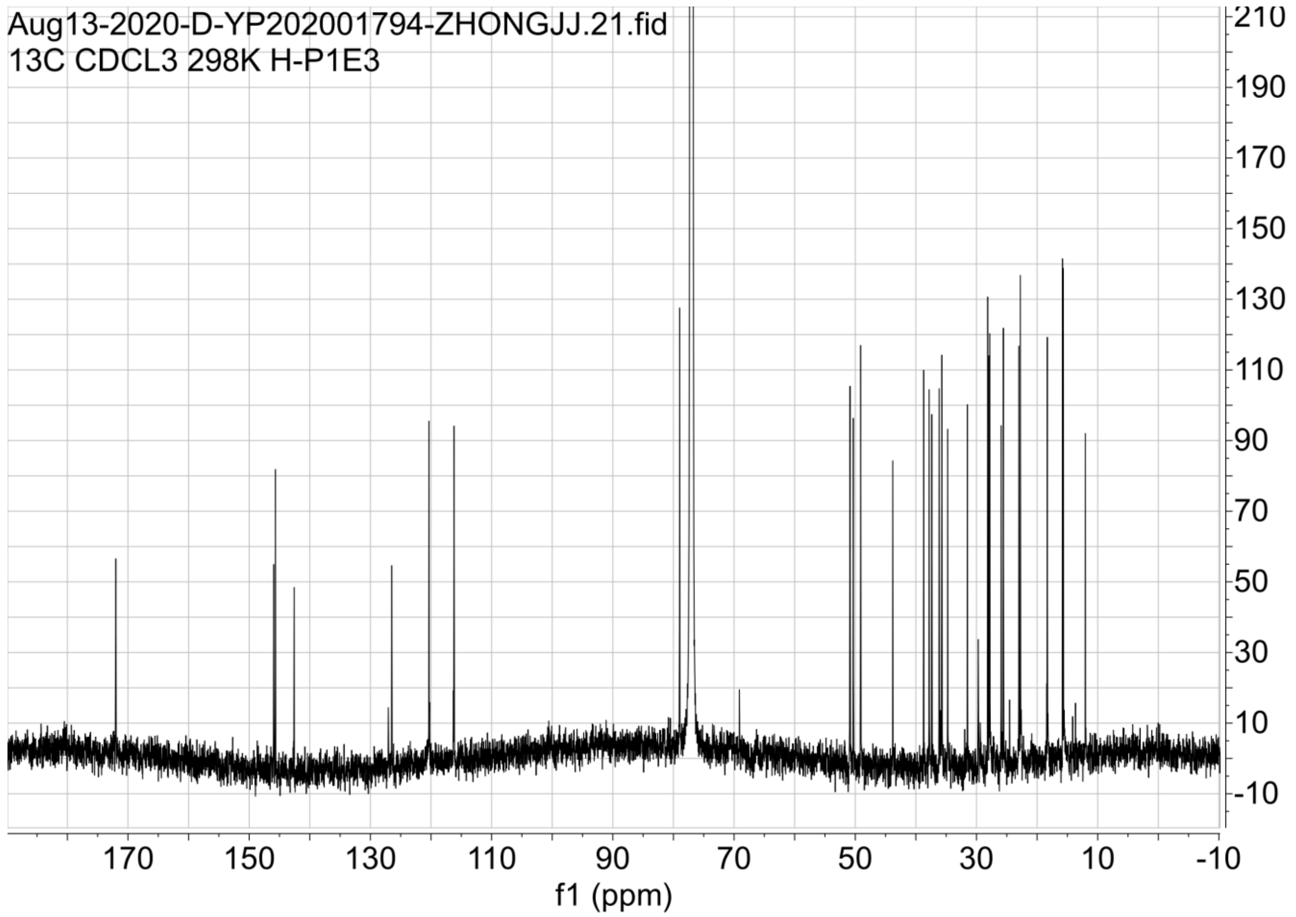

Supplementary Figure 16. ^{13}C NMR spectrum of 3.

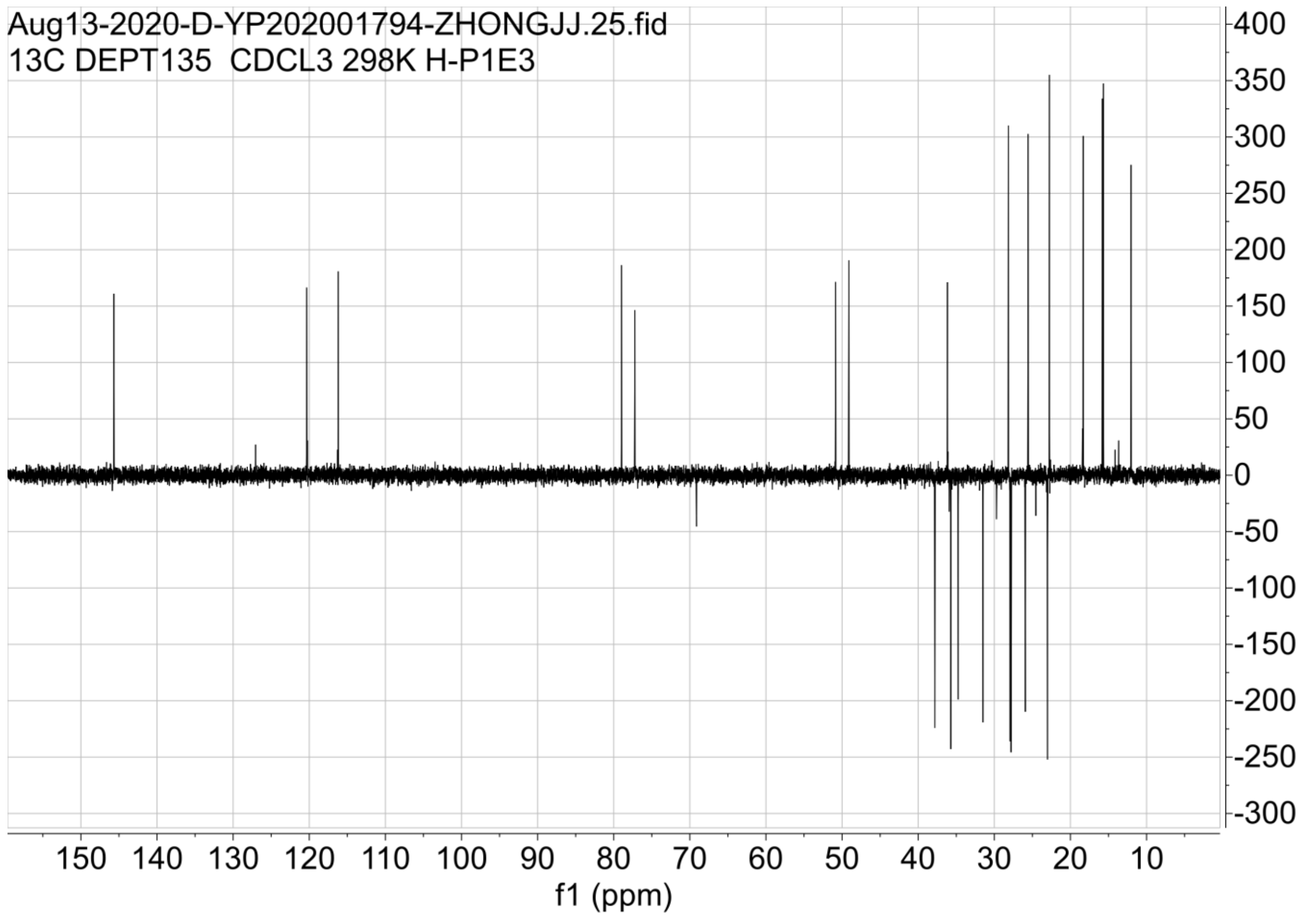

Supplementary Figure 17. DEPT-135 spectrum of 3.

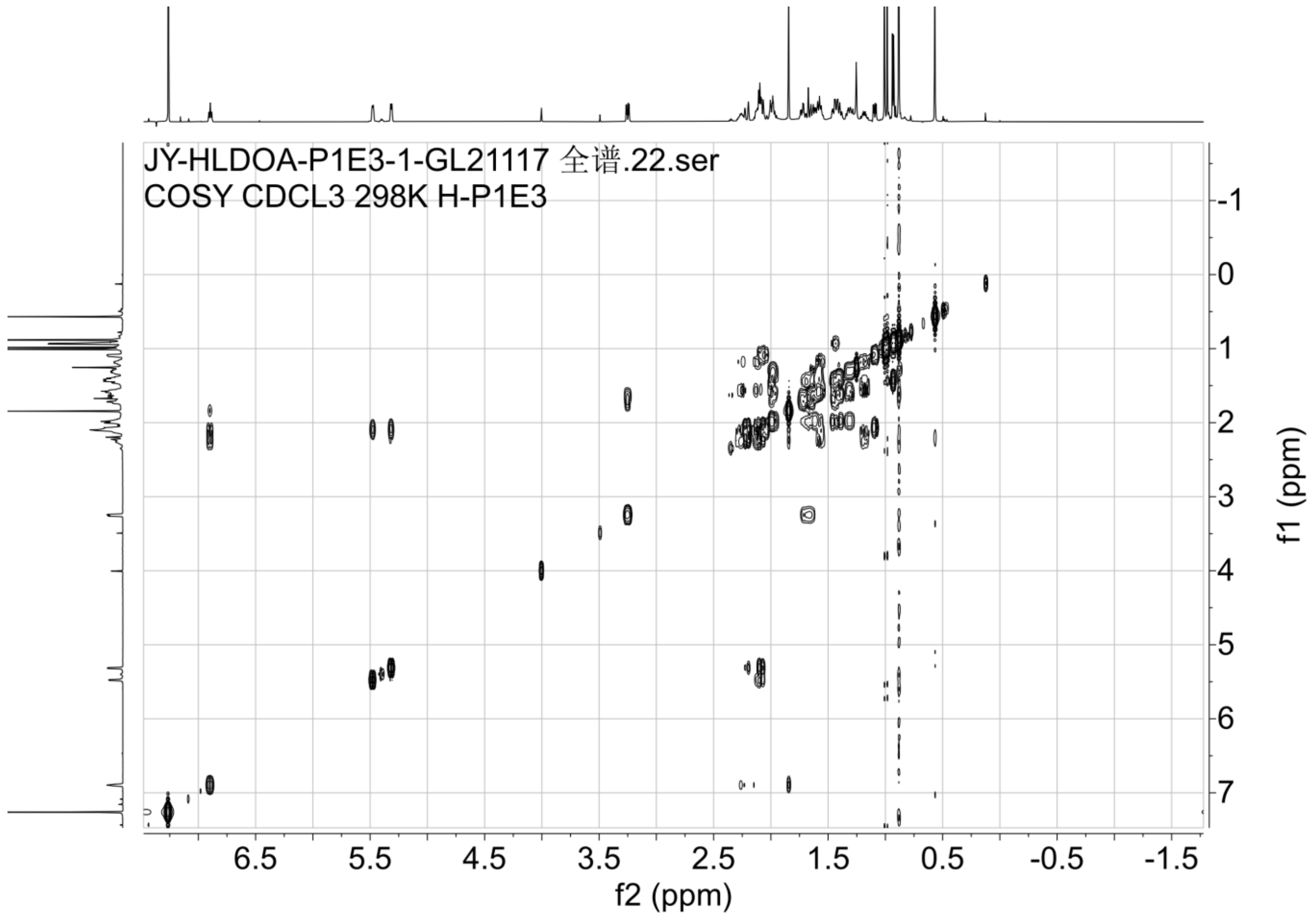

Supplementary Figure 18. COSY spectrum of 3.

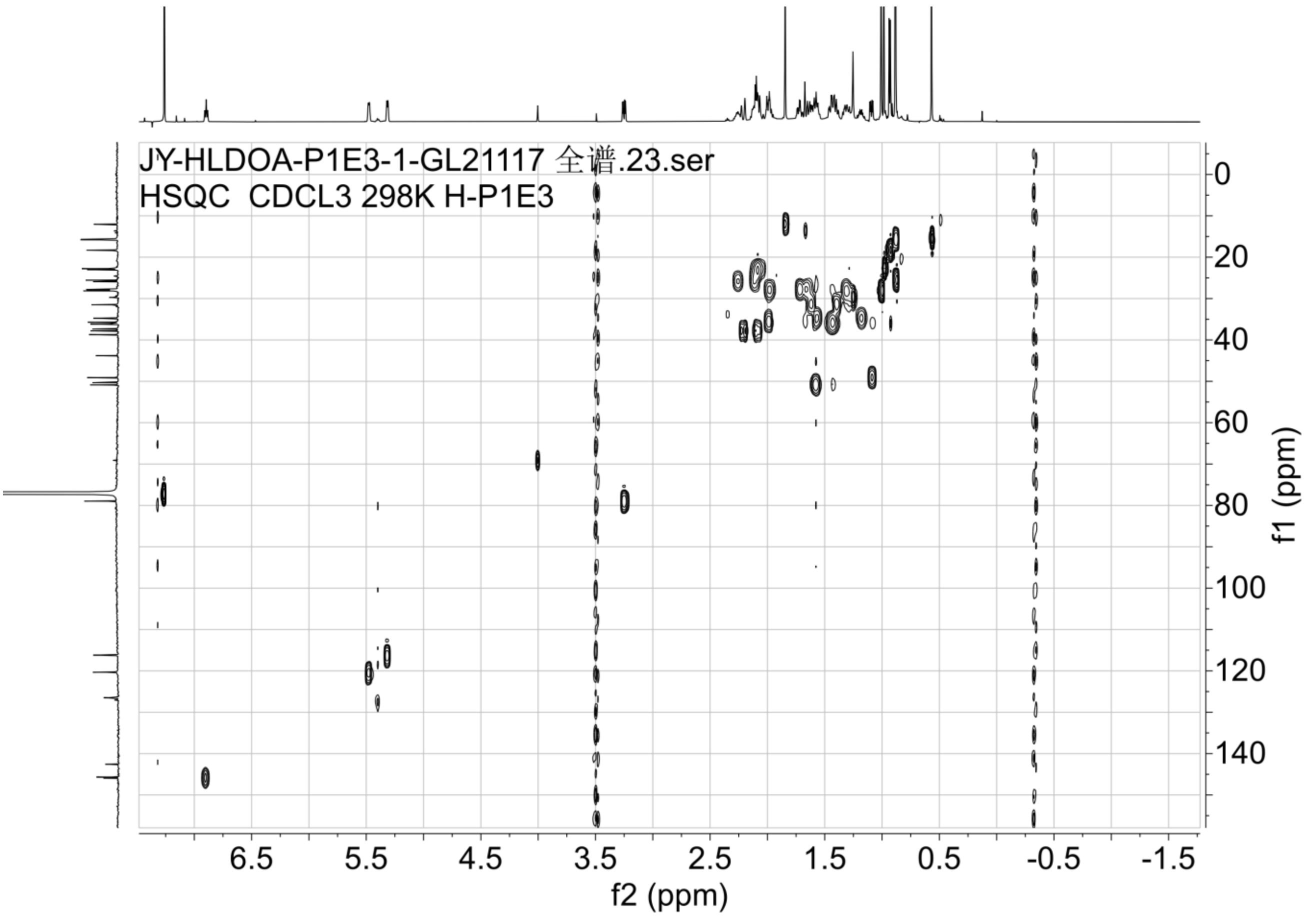

Supplementary Figure 19. HSQC spectrum of 3.

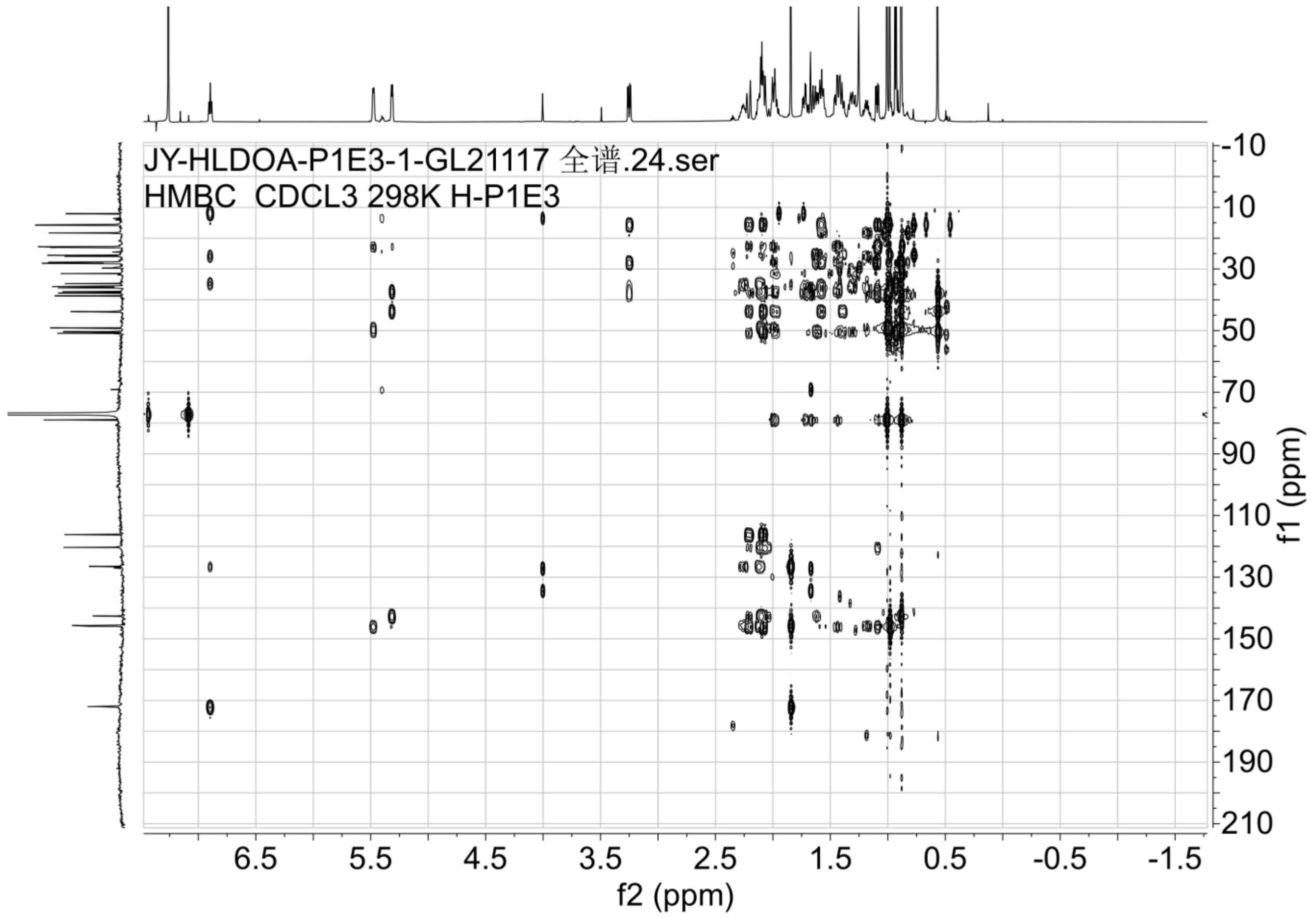

Supplementary Figure 20. HMBC spectrum of 3.

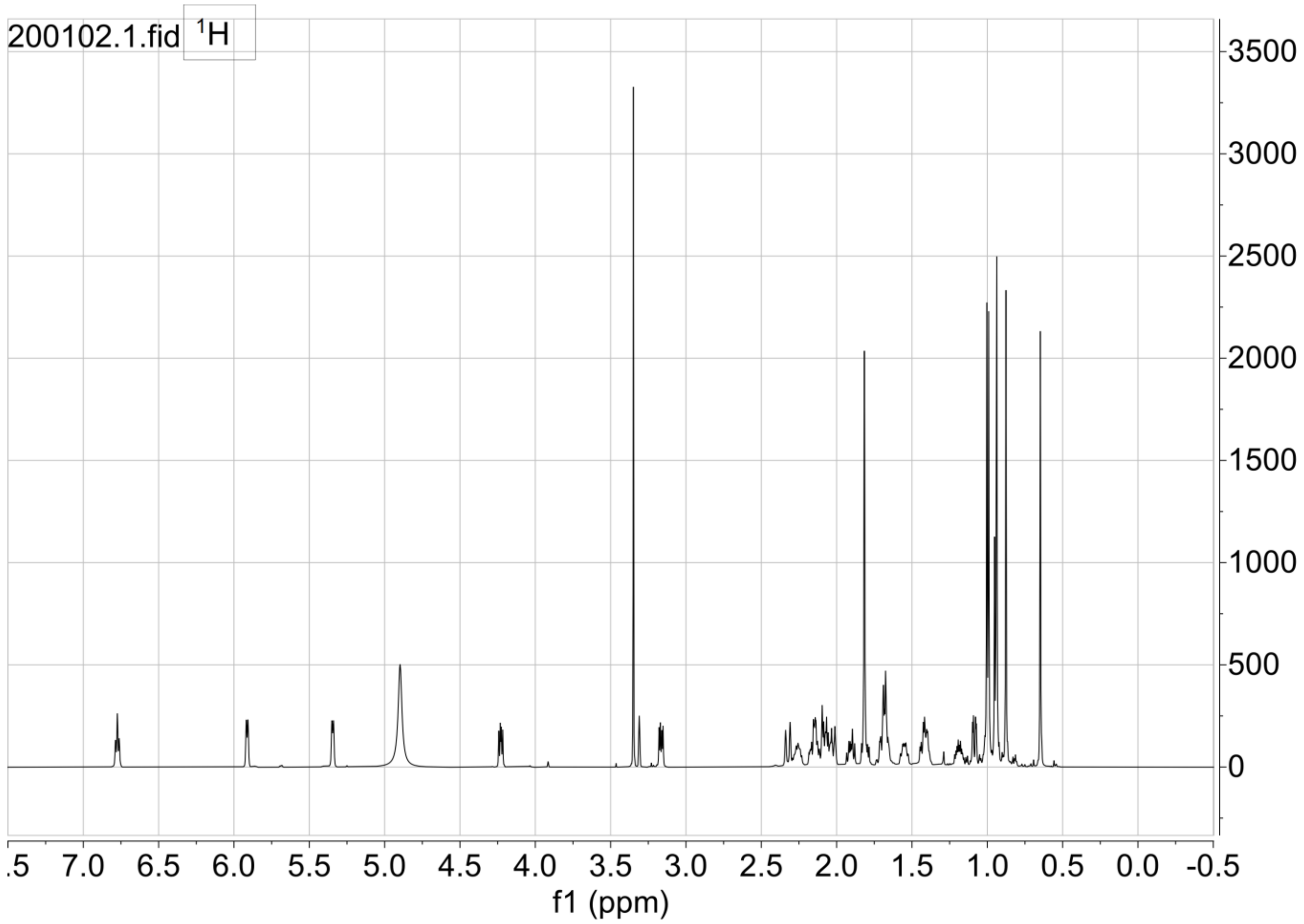

Supplementary Figure 21. ^{1}H NMR spectrum of 4.

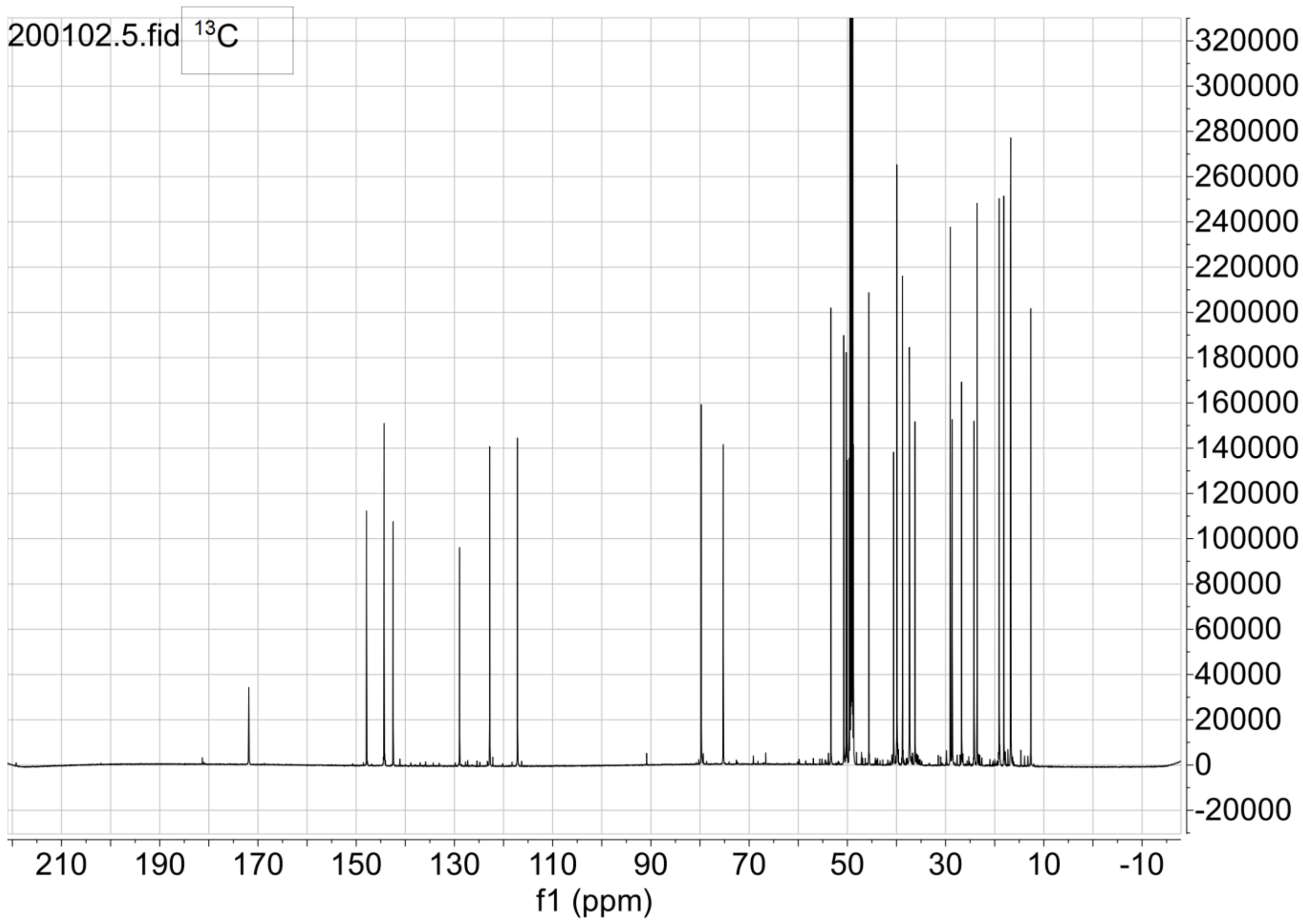

Supplementary Figure 22. ^{13}C NMR spectrum of 4.

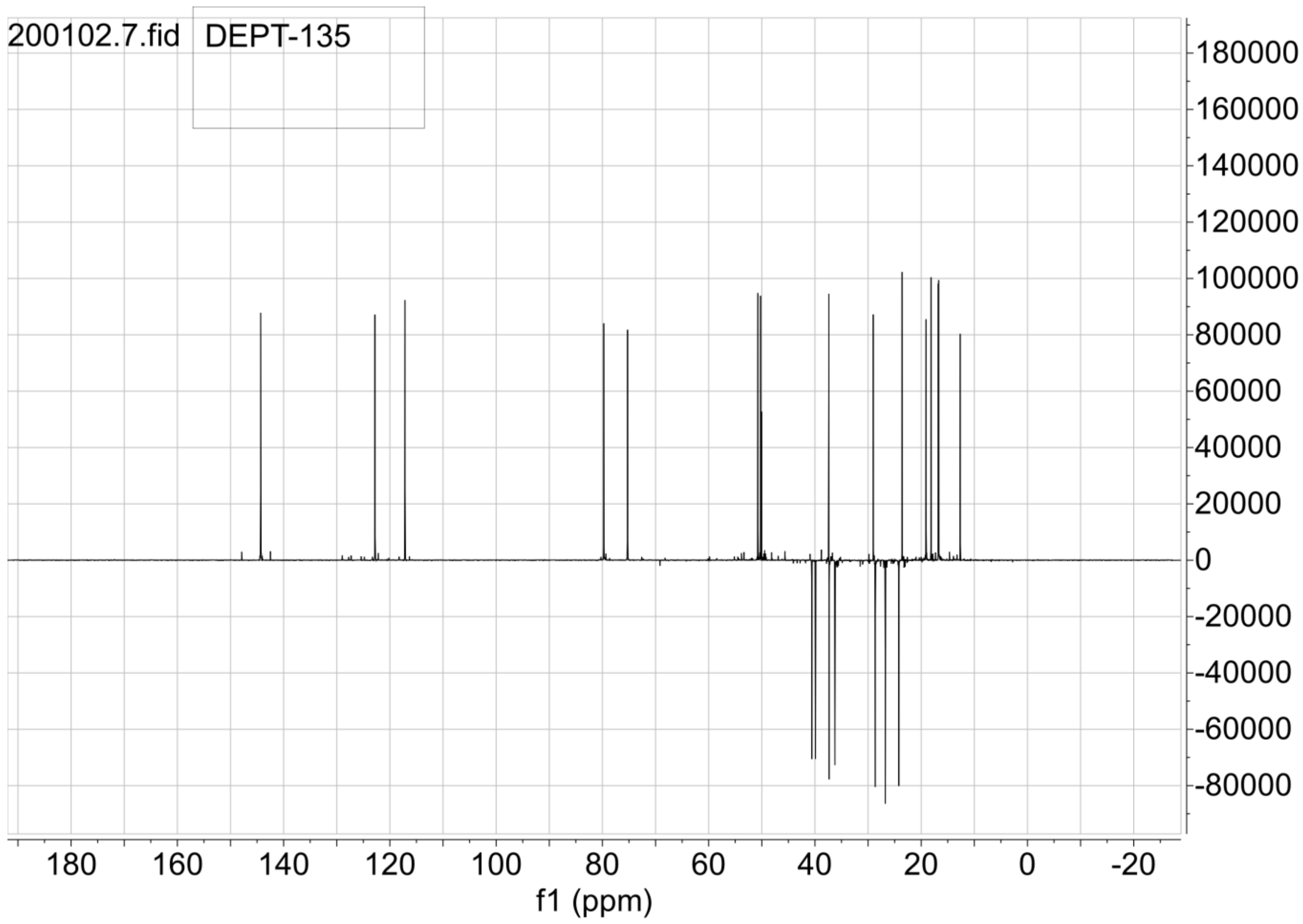

Supplementary Figure 23. DEPT-135 spectrum of 4.

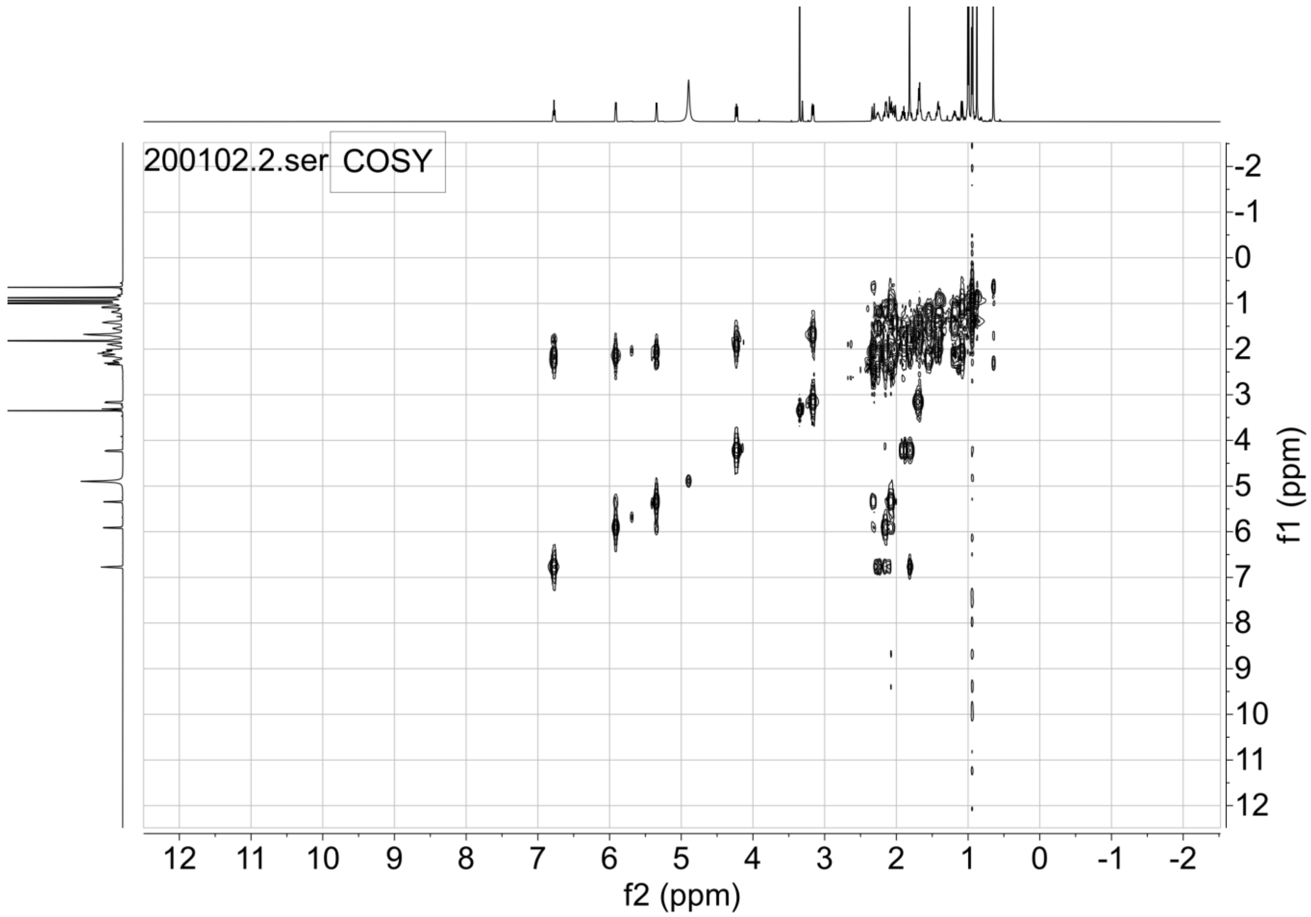

Supplementary Figure 24. COSY spectrum of 4.

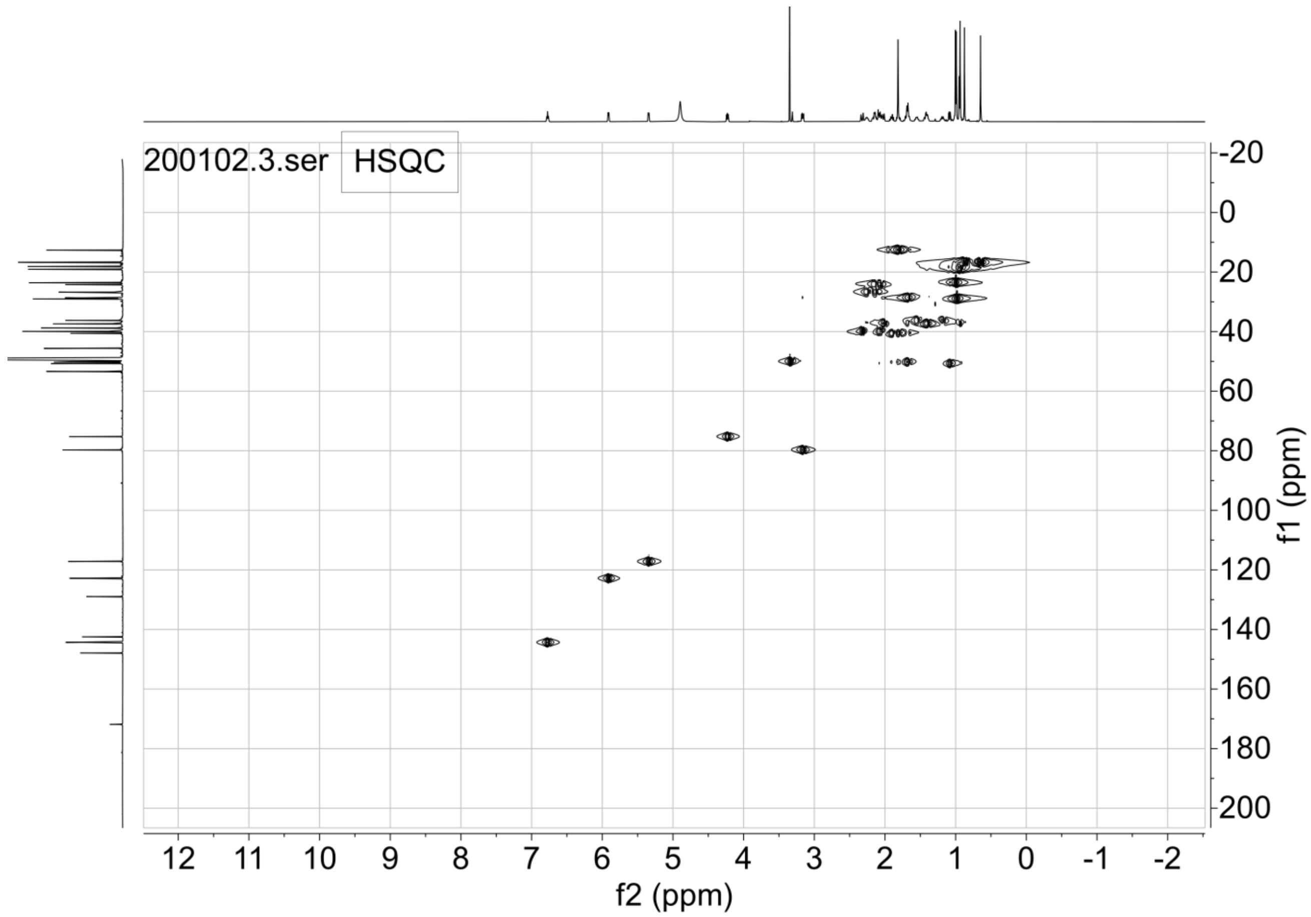

Supplementary Figure 25. HSQC spectrum of 4.

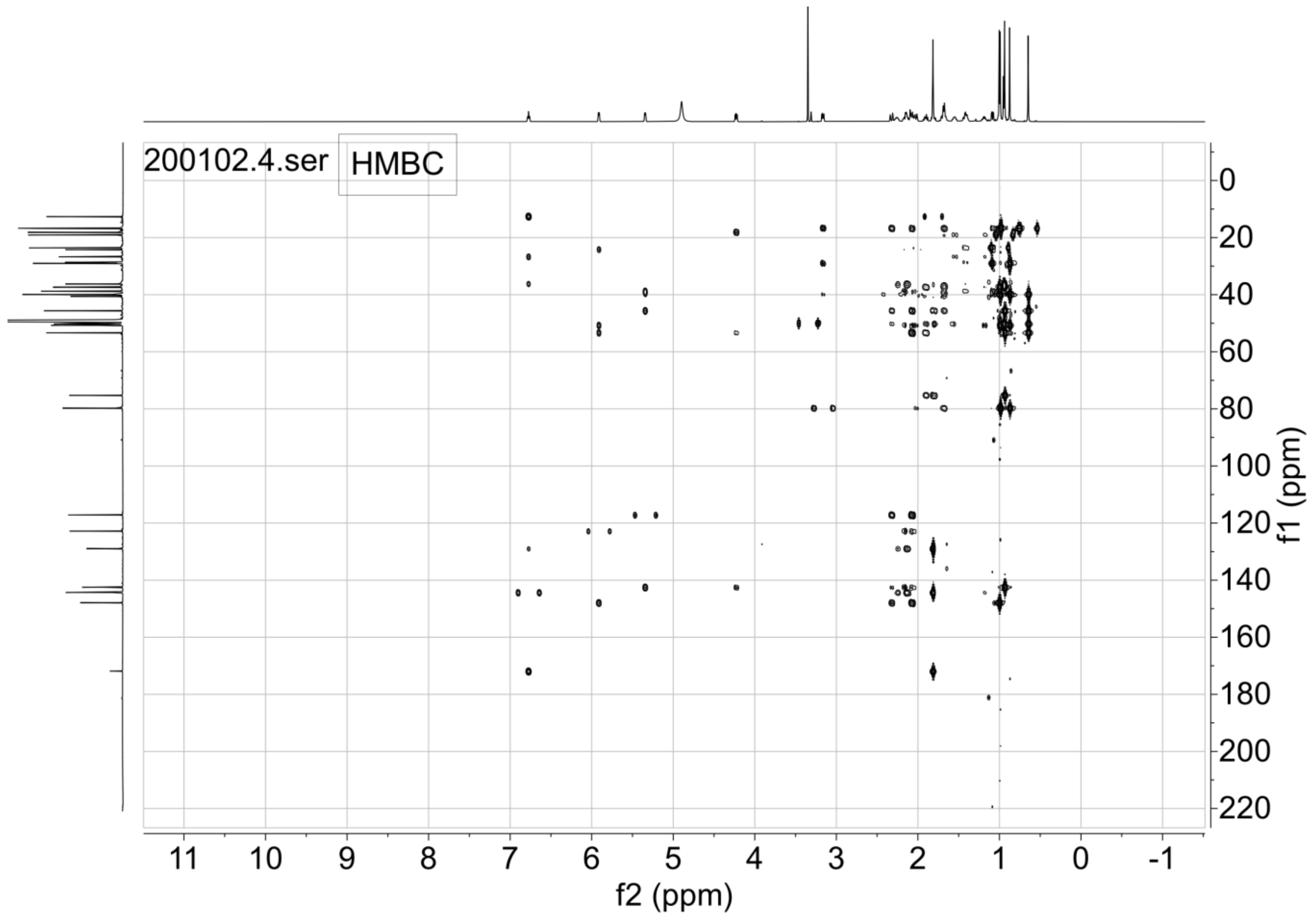

Supplementary Figure 26. HMBC spectrum of 4.

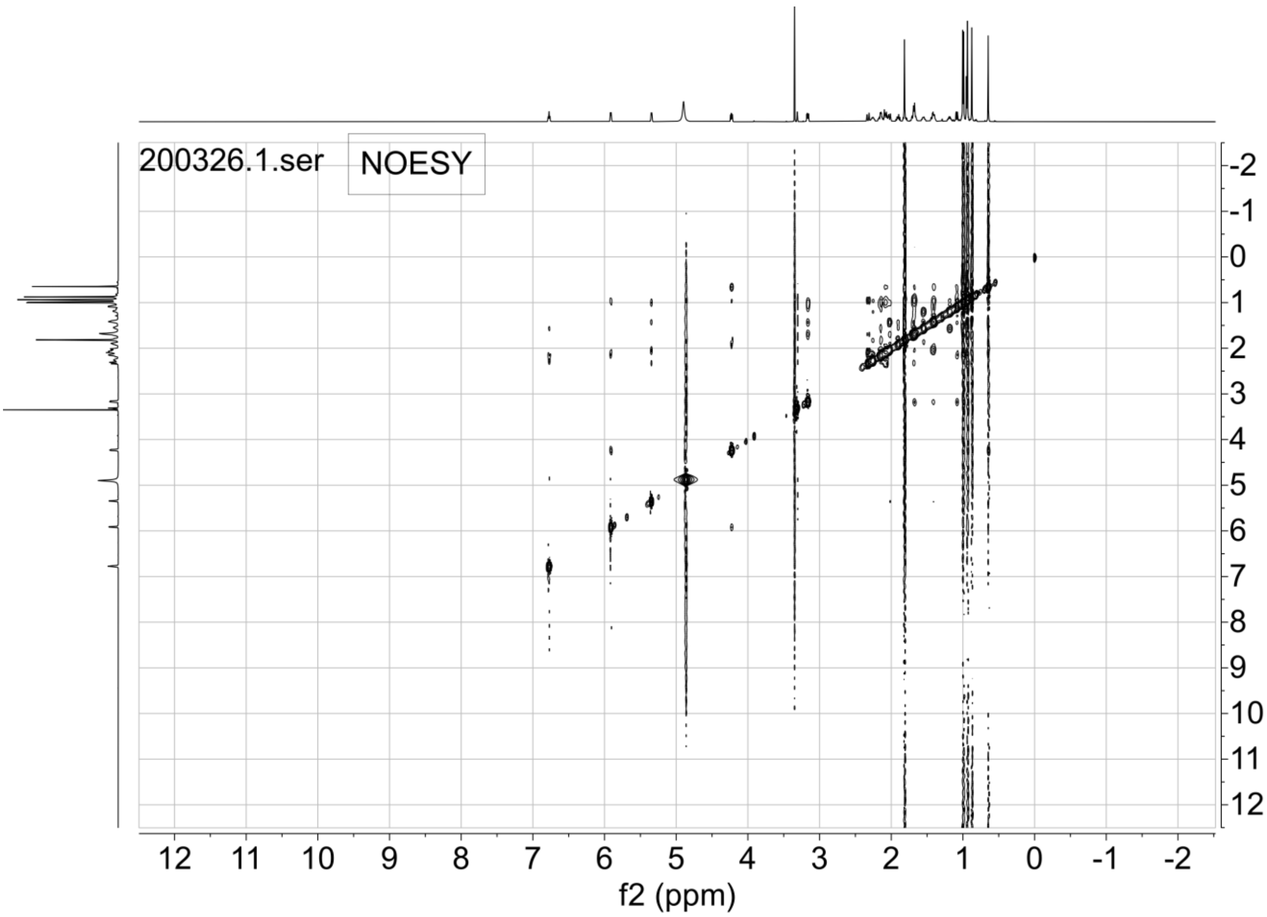

Supplementary Figure 27. NOESY spectrum of 4.

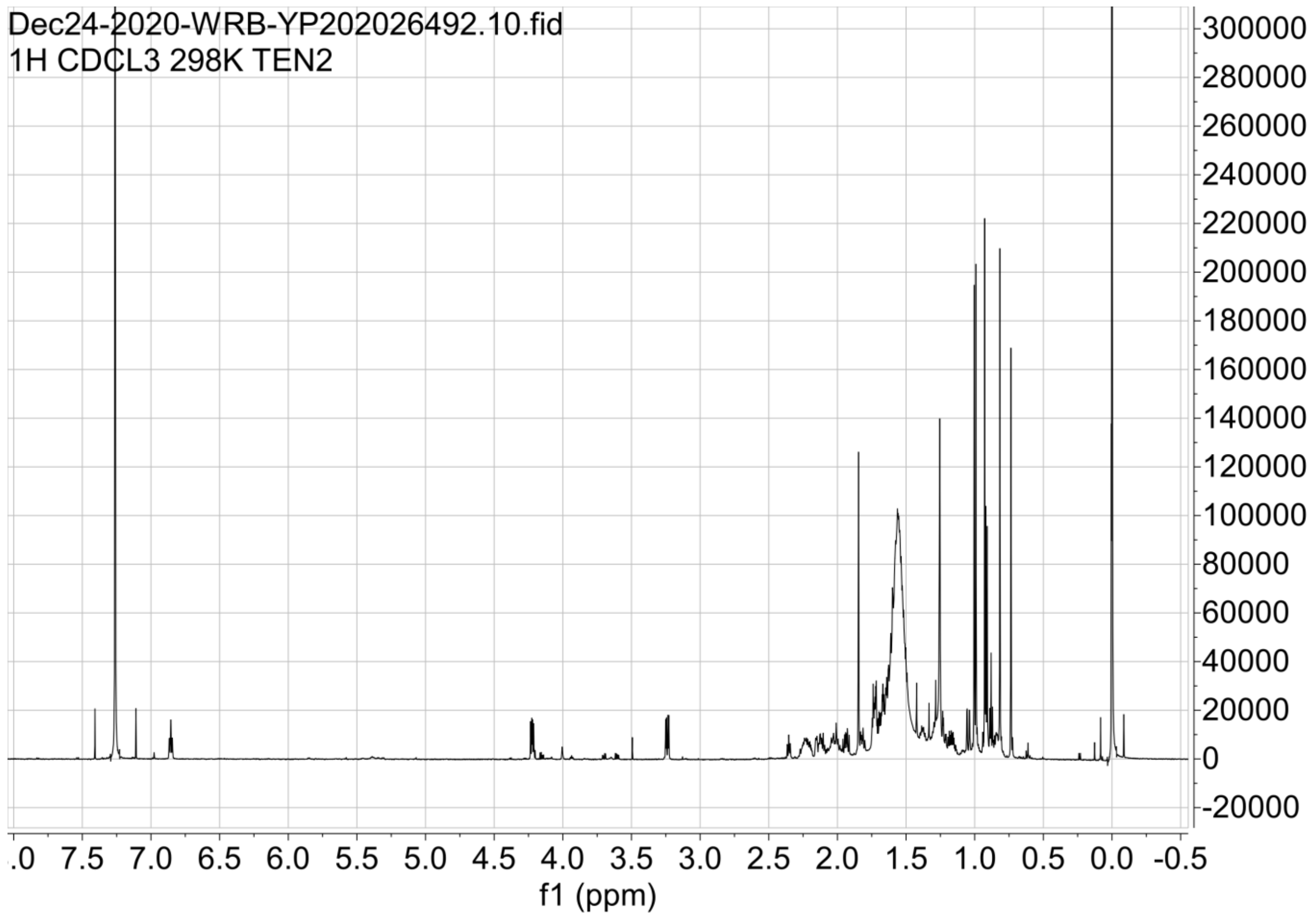

Supplementary Figure 28. ^{1}H NMR spectrum of 5.

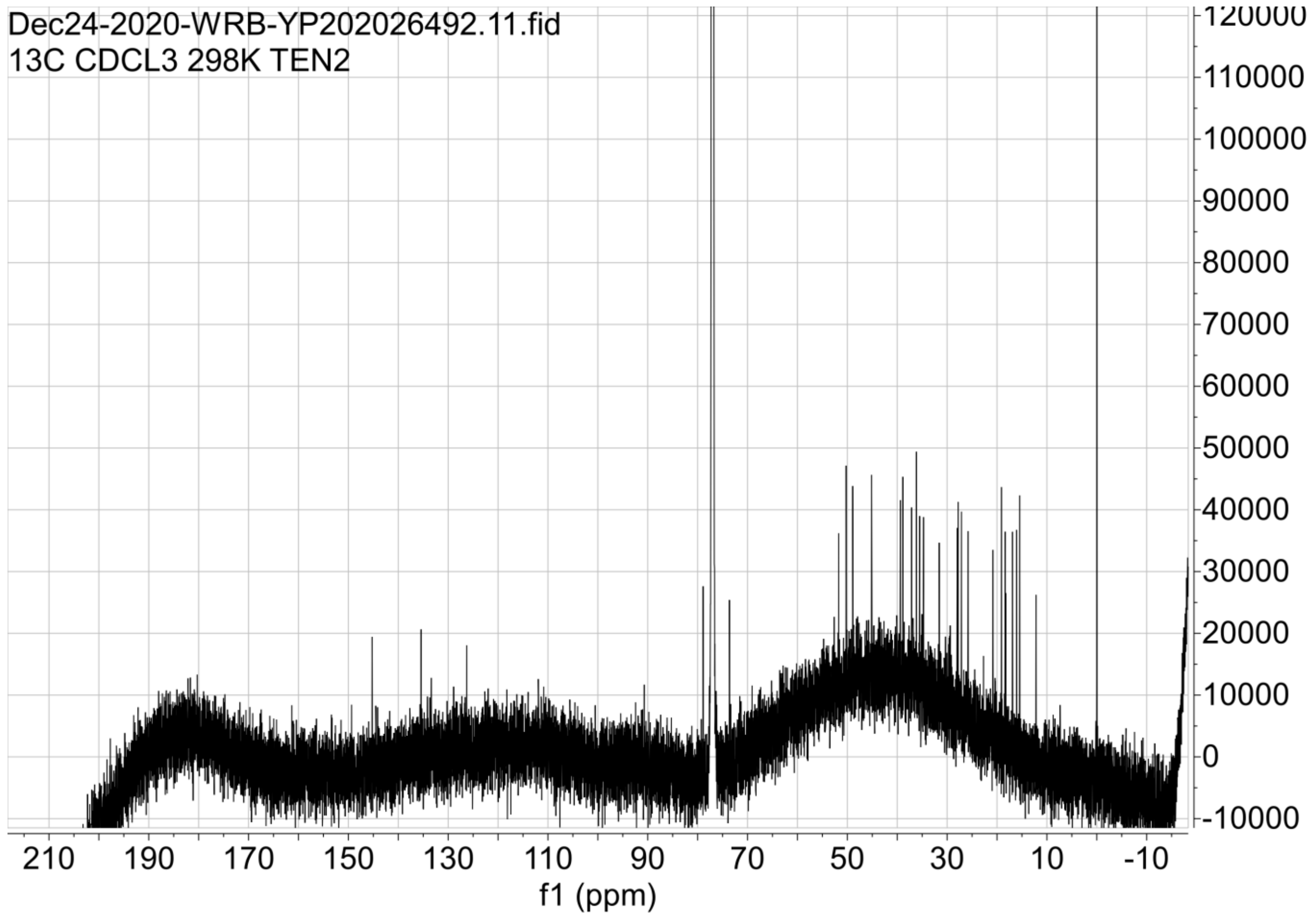

Supplementary Figure 29. ^{13}C NMR spectrum of 5.

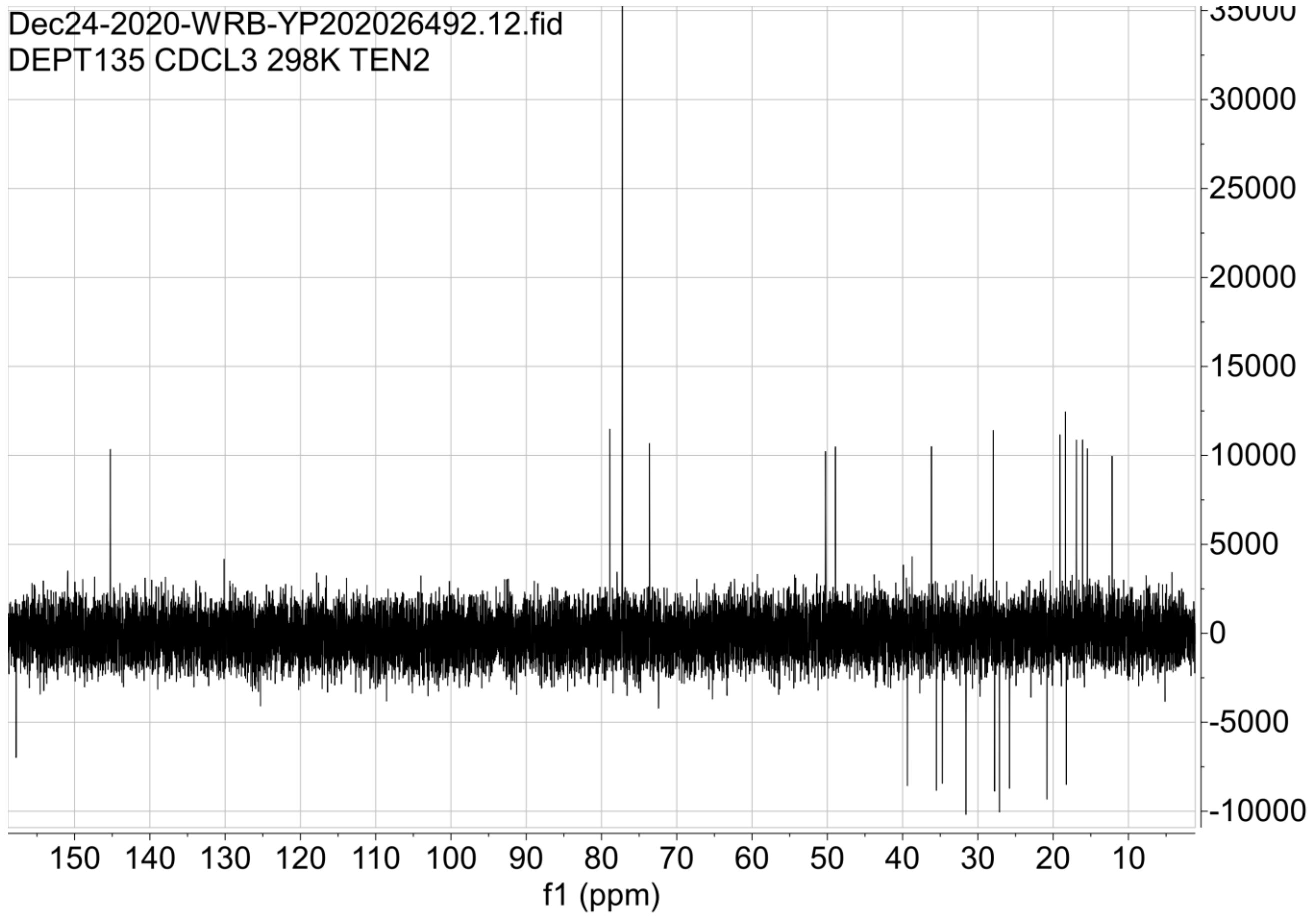

Supplementary Figure 30. DEPT-135 spectrum of 5.

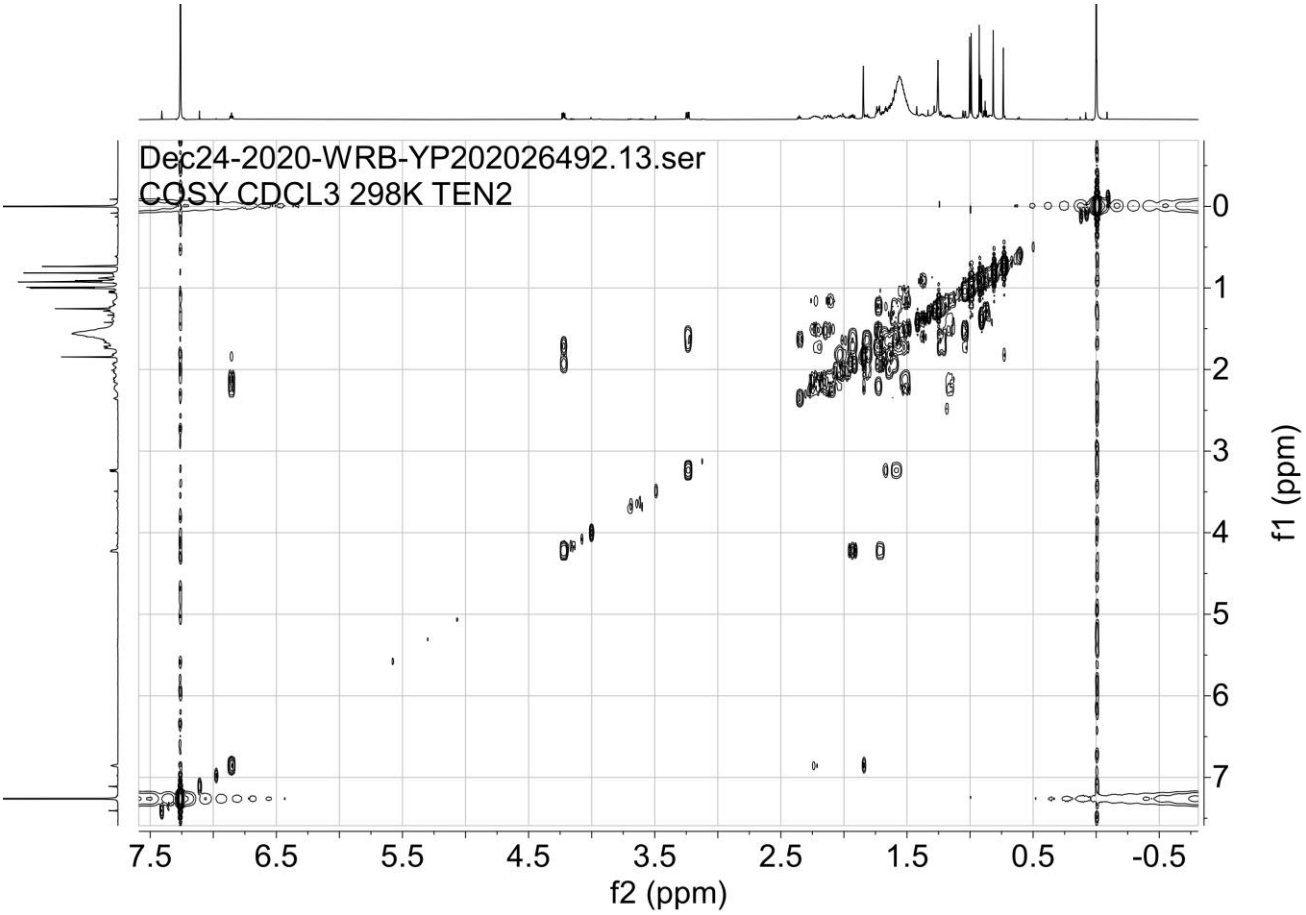

Supplementary Figure 31. COSY spectrum of 5.

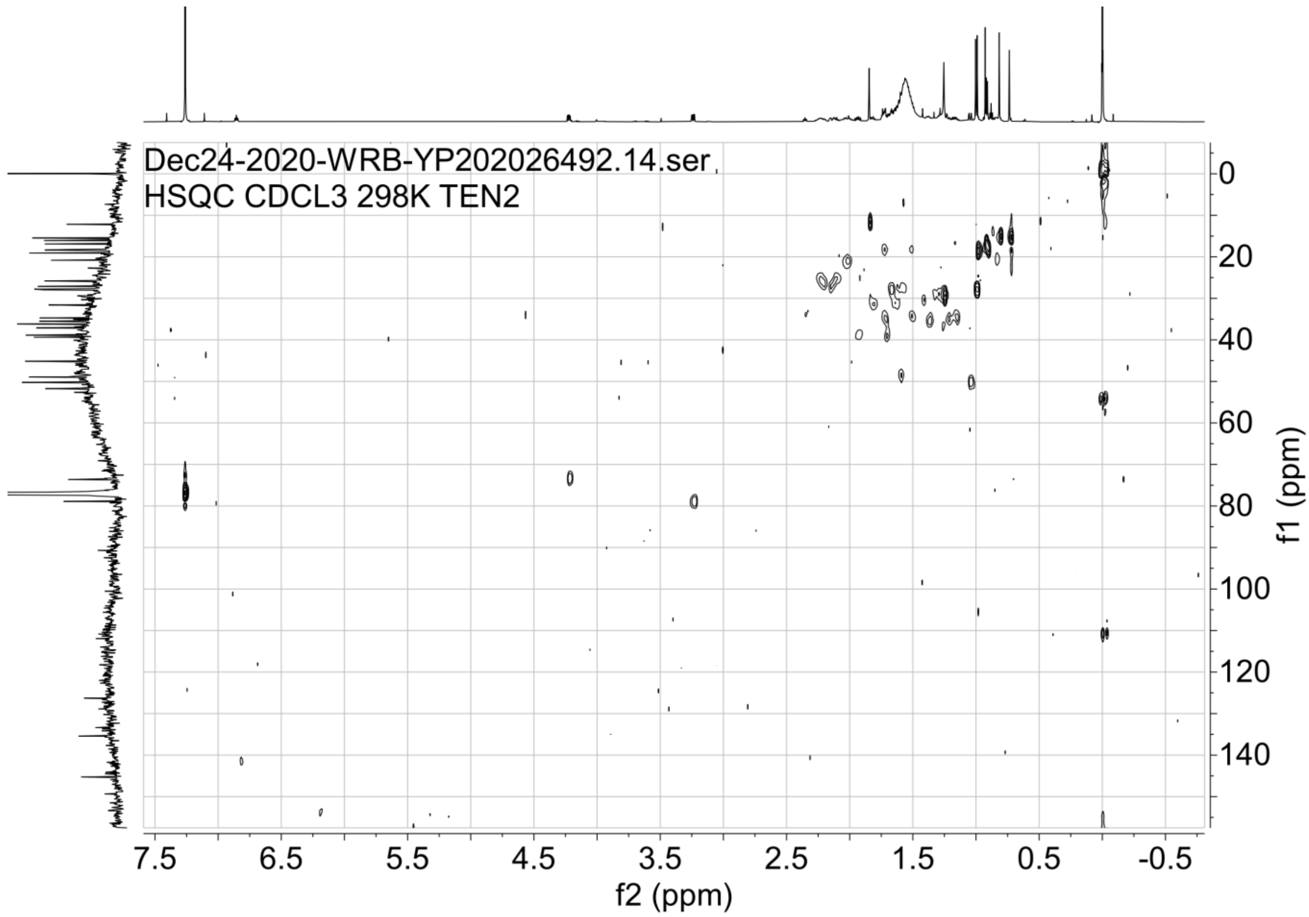

Supplementary Figure 32. HSQC spectrum of 5.

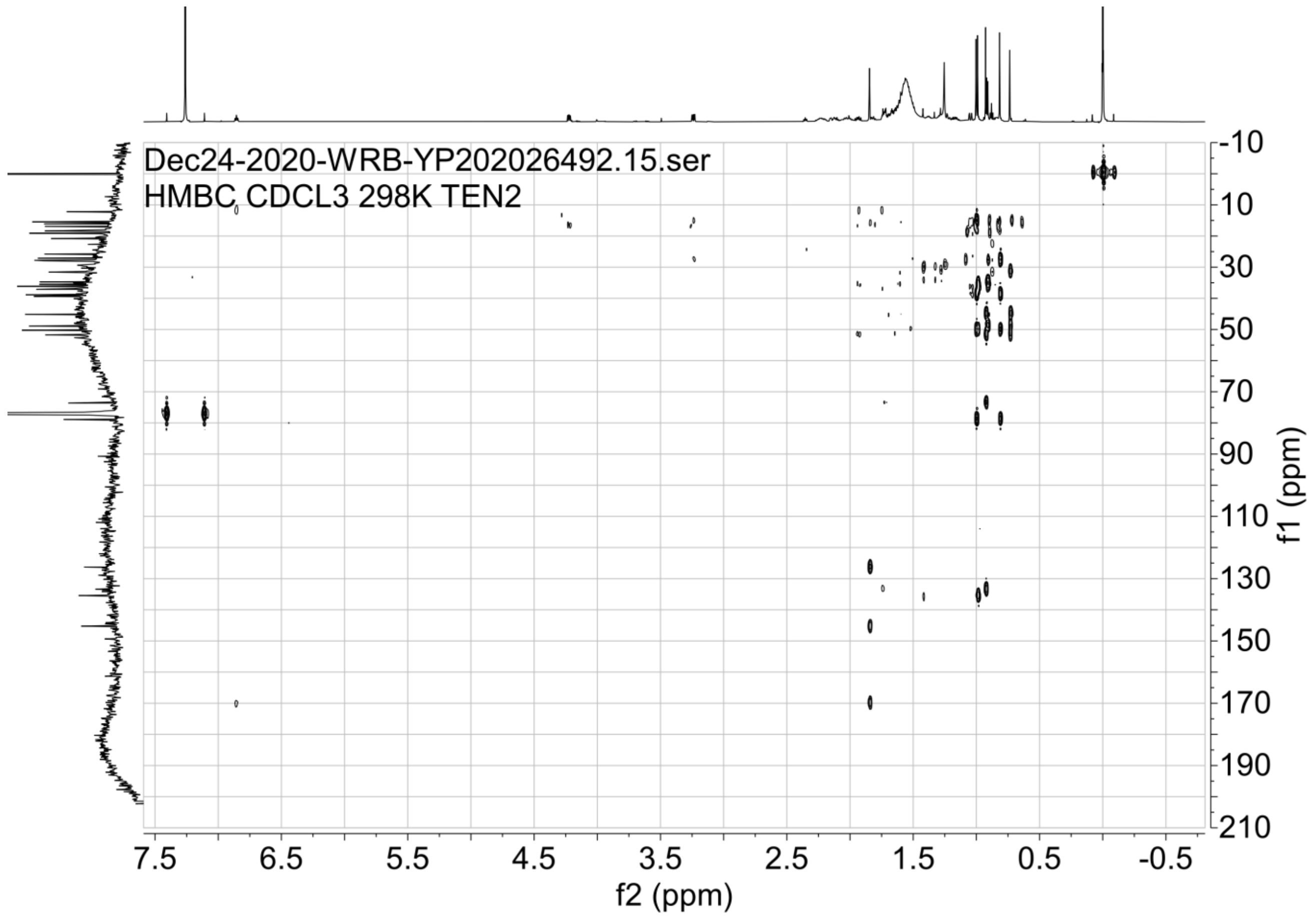

Supplementary Figure 33. HMBC spectrum of 5.

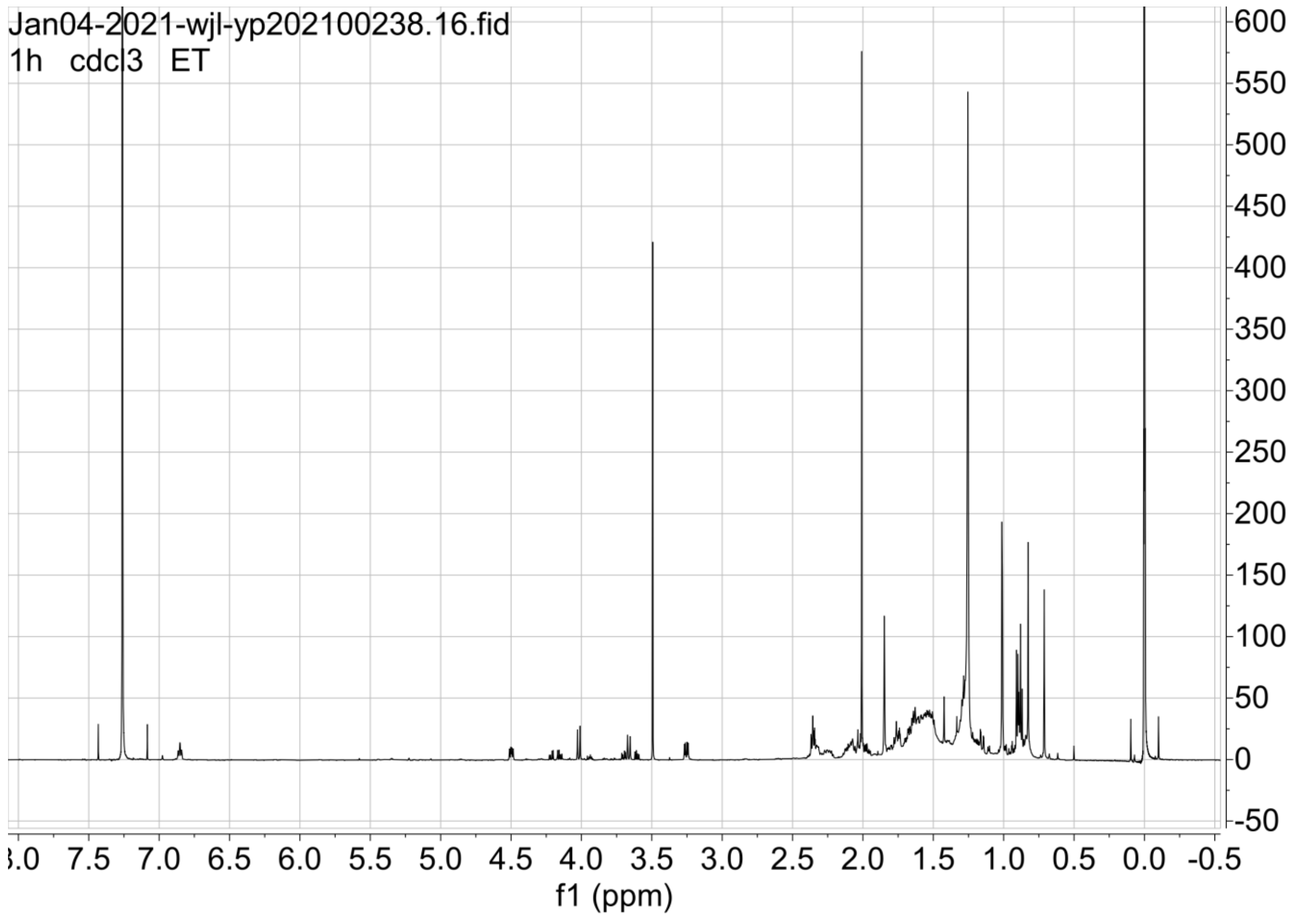

Supplementary Figure 34. ^{1}H NMR spectrum of compound 6.

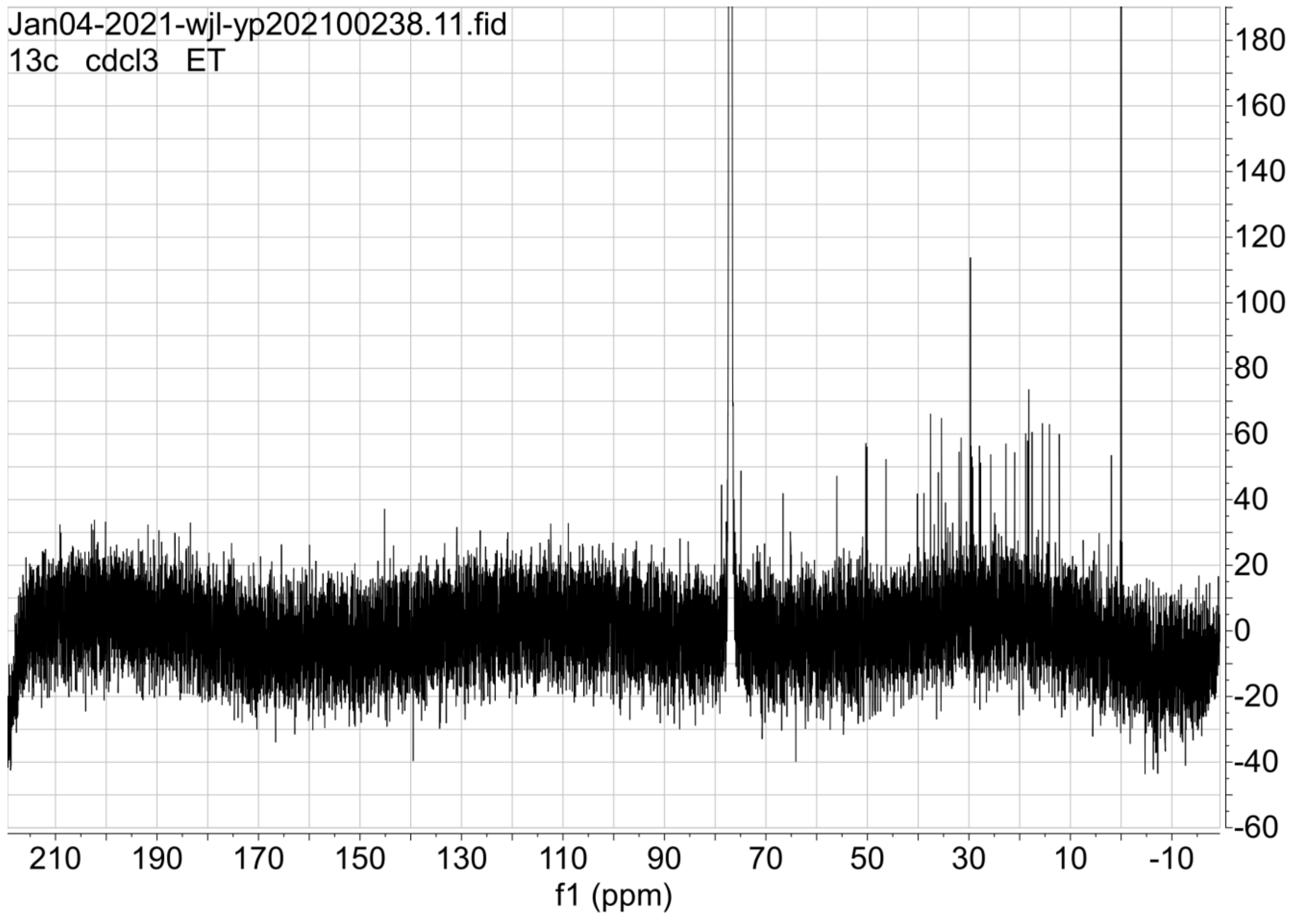

Supplementary Figure 35. ^{13}C NMR spectrum of 6.

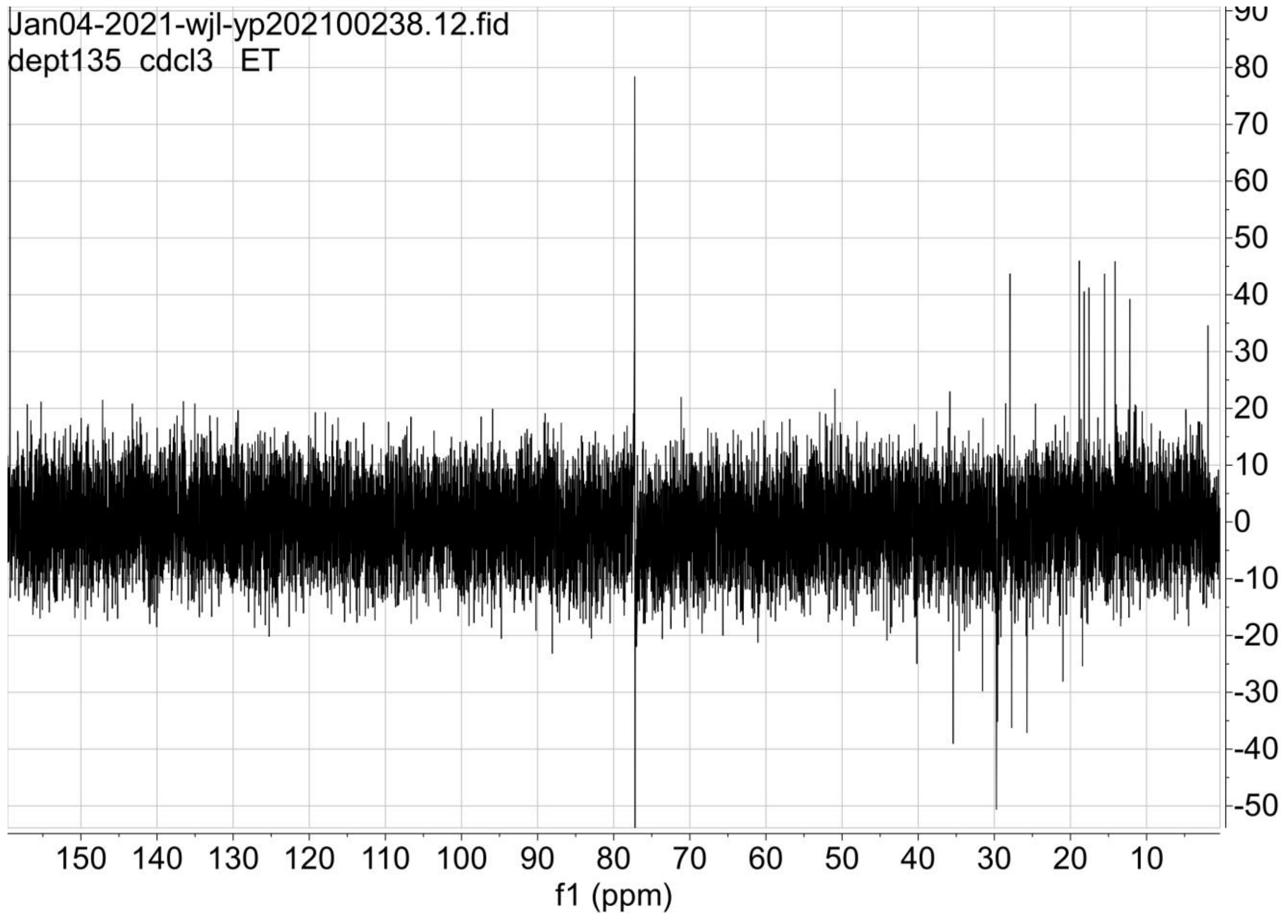

Supplementary Figure 36. DEPT-135 spectrum of 6.

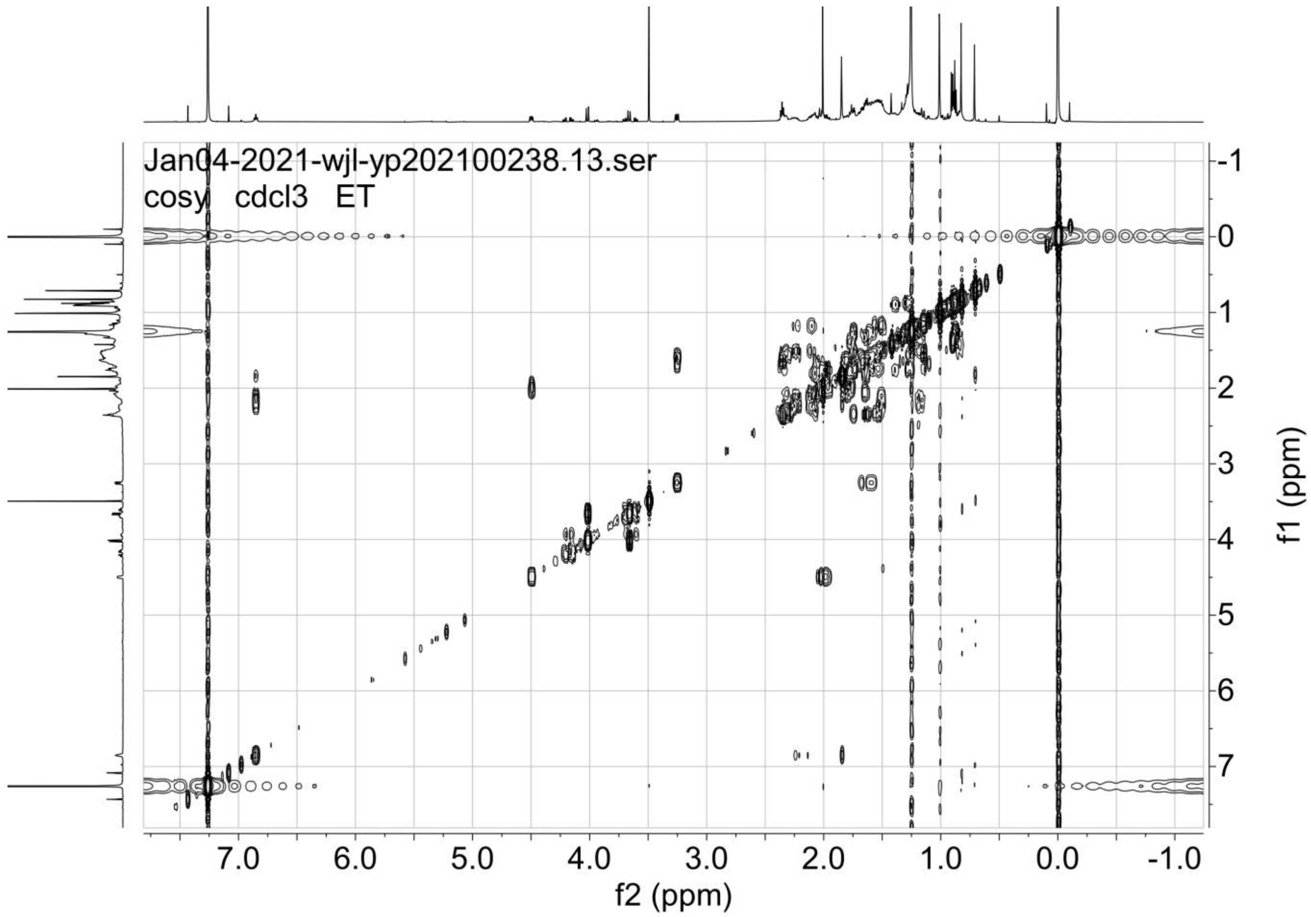

Supplementary Figure 37. COSY spectrum of 6.

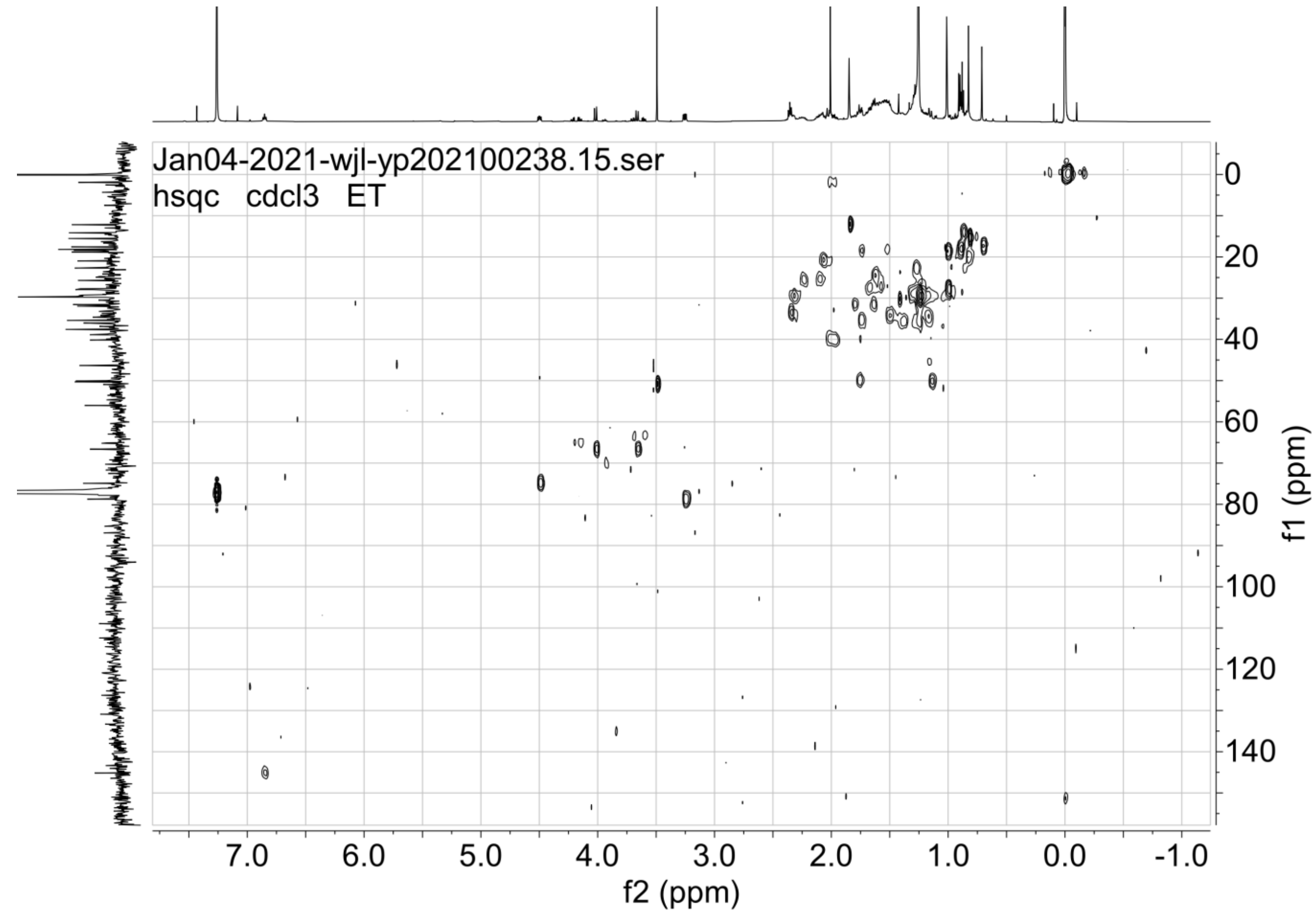

Supplementary Figure 38. HSQC spectrum of 6.

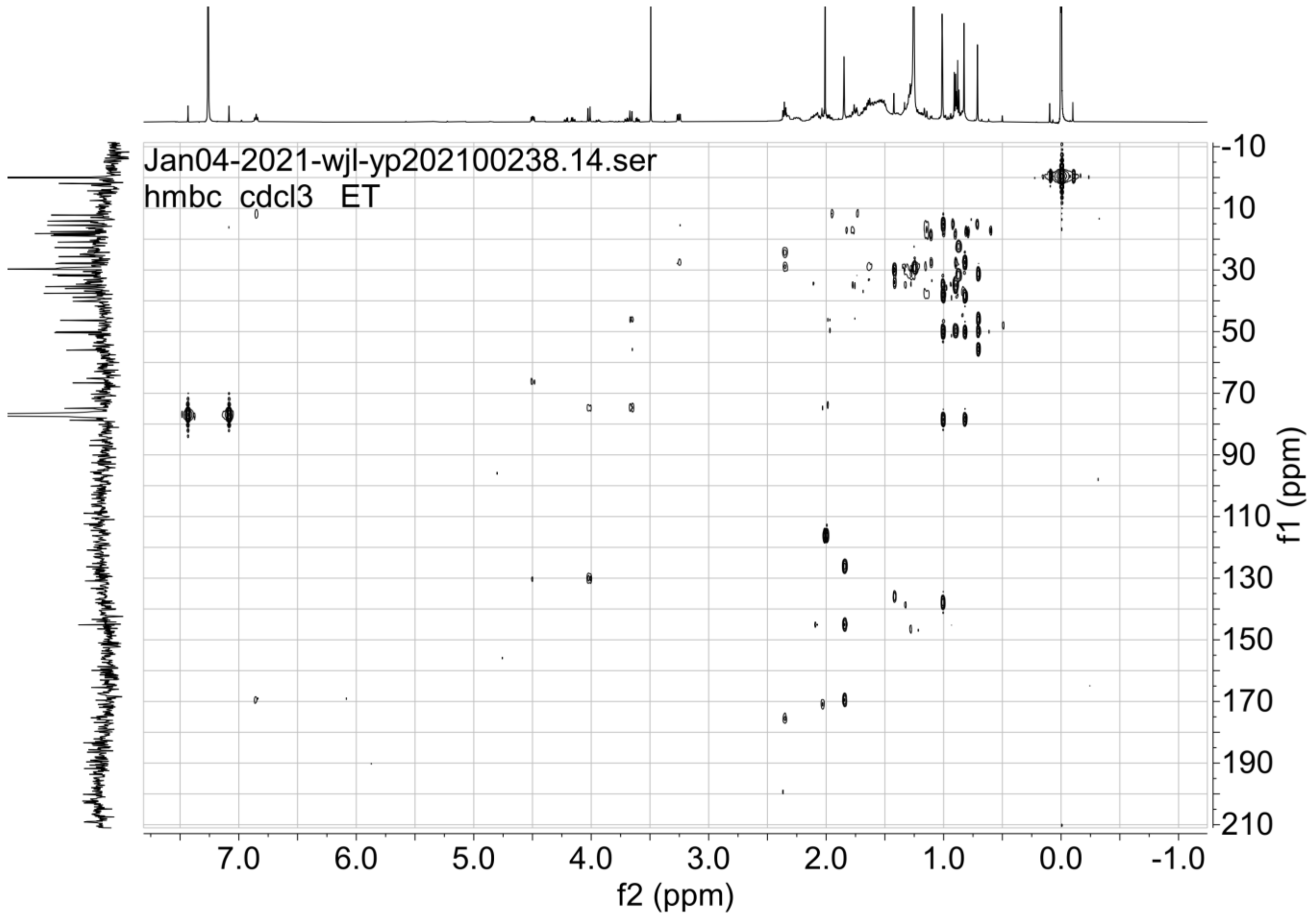

Supplementary Figure 39. HMBC spectrum of 6.

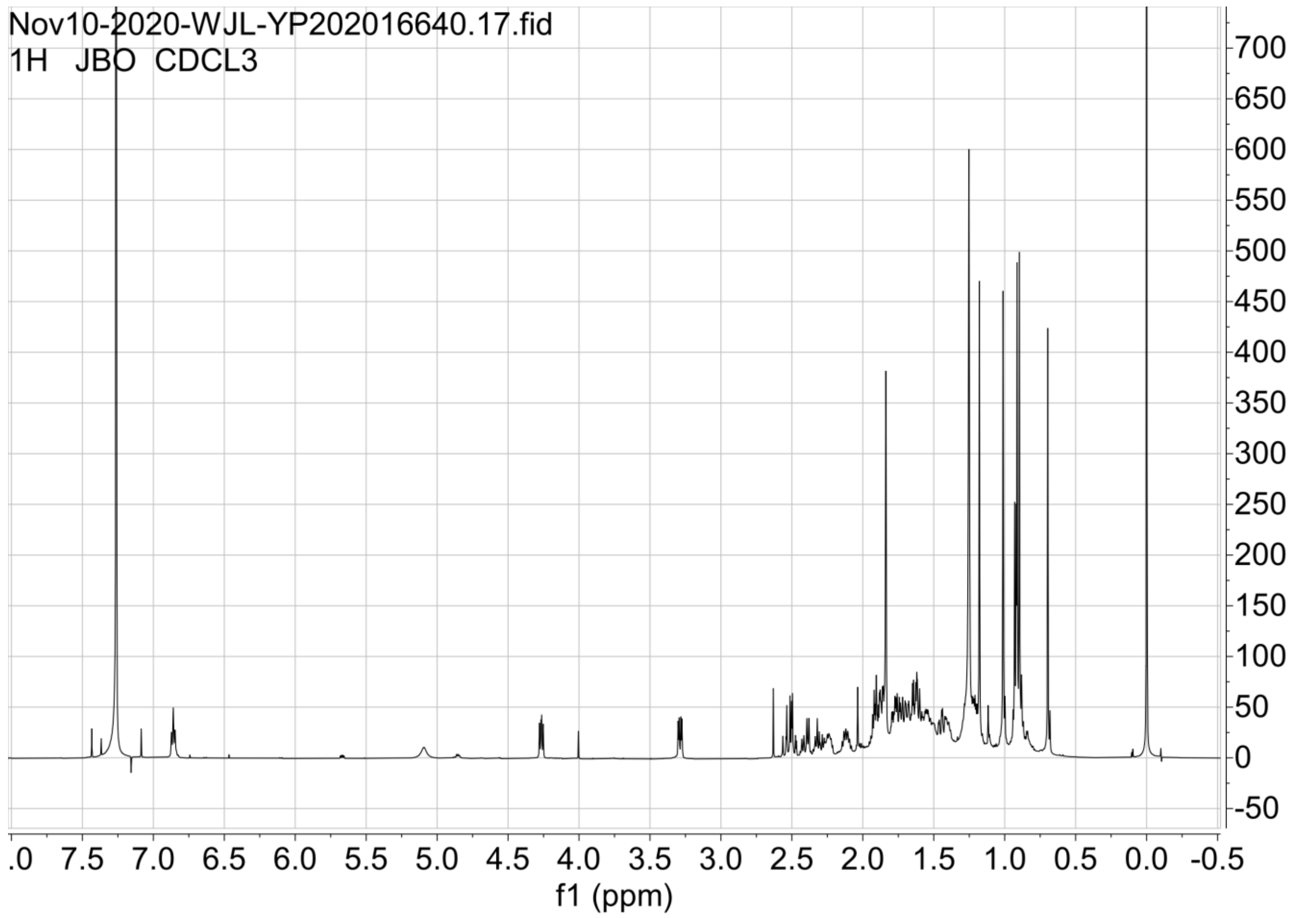

Supplementary Figure 40. ^{1}H NMR spectrum of 7.

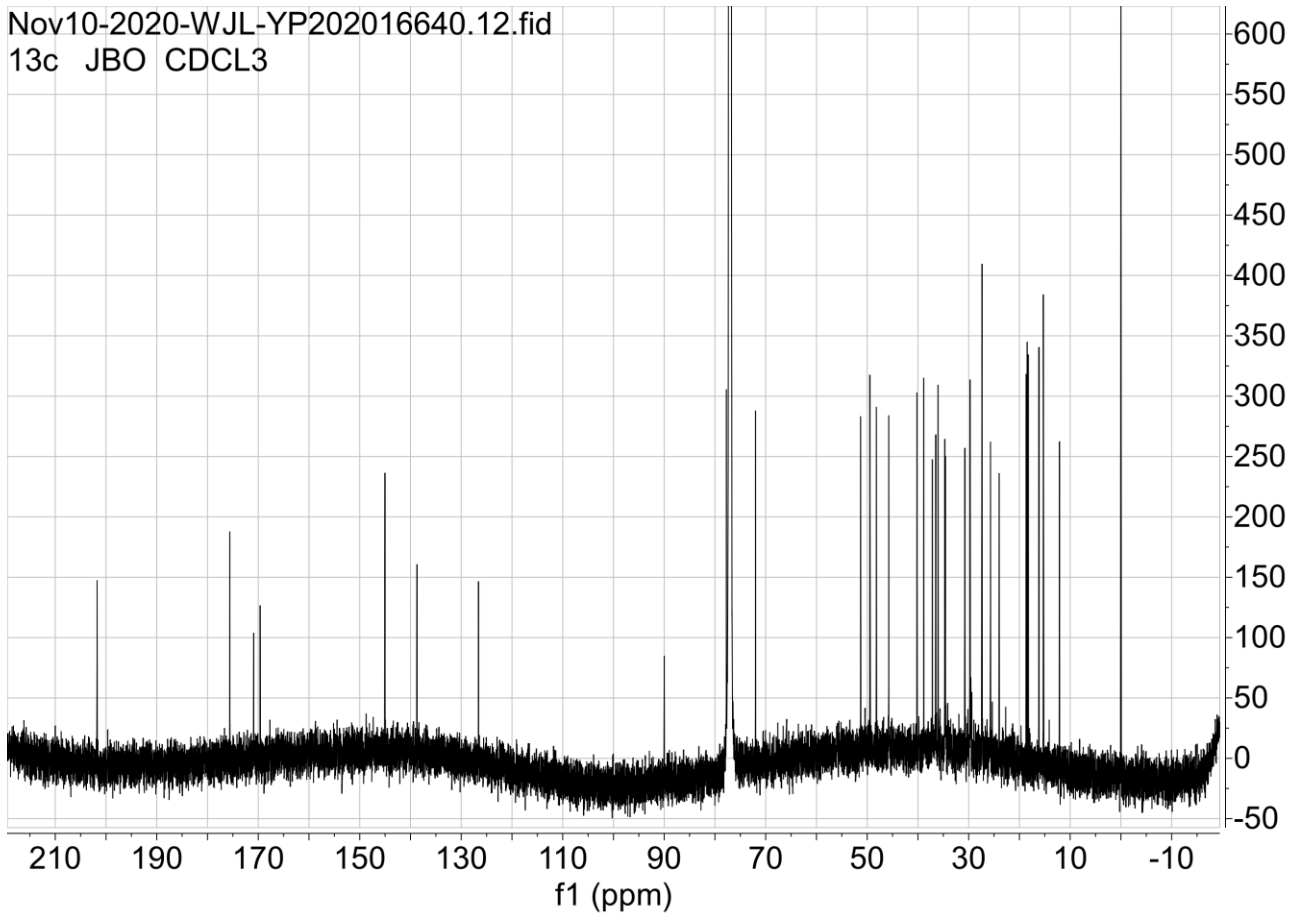

Supplementary Figure 41. ^{13}C NMR spectrum of 7.

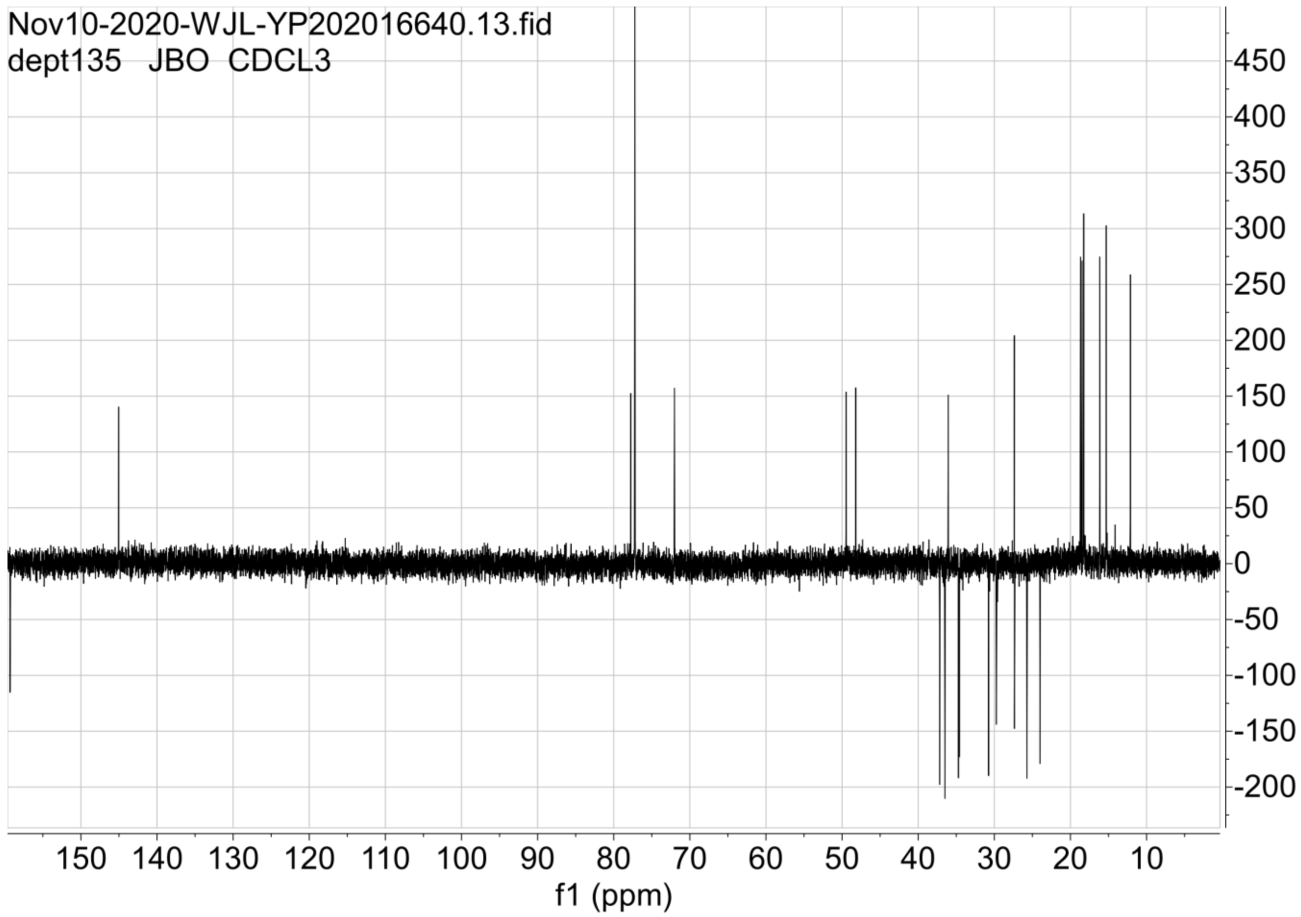

Supplementary Figure 42. DEPT-135 spectrum of 7.

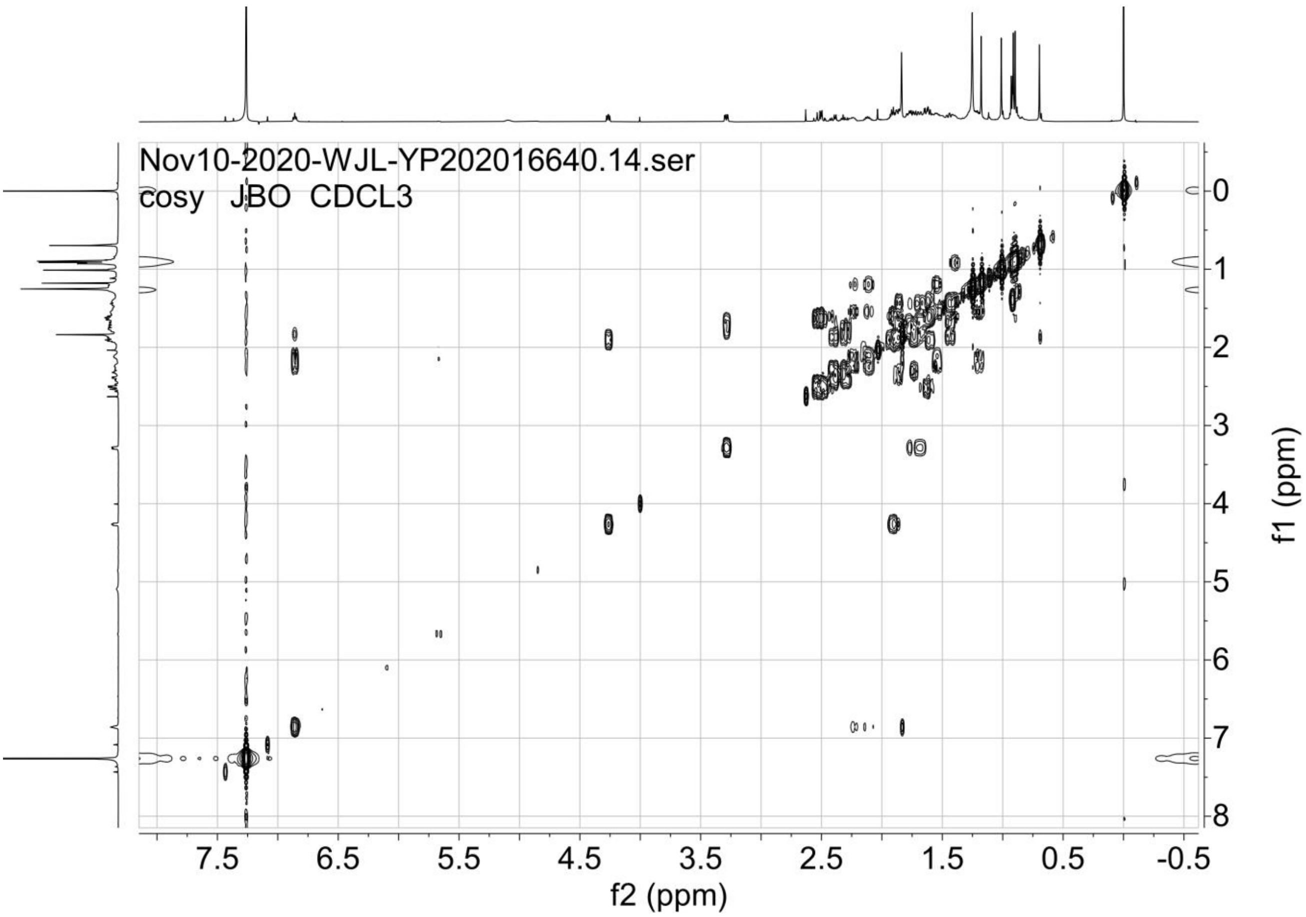

Supplementary Figure 43. COSY spectrum of 7.

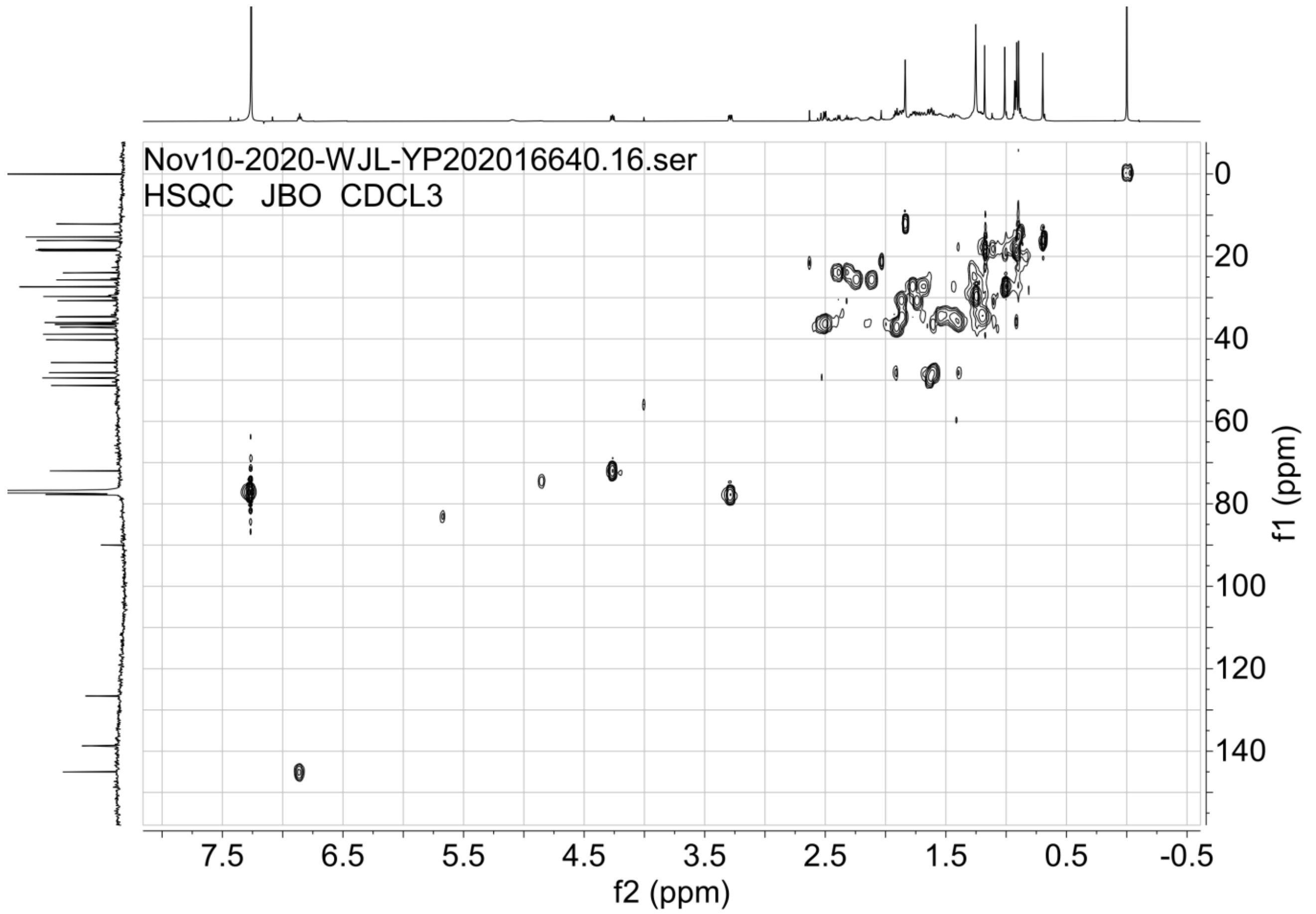

Supplementary Figure 44. HSQC spectrum of 7.

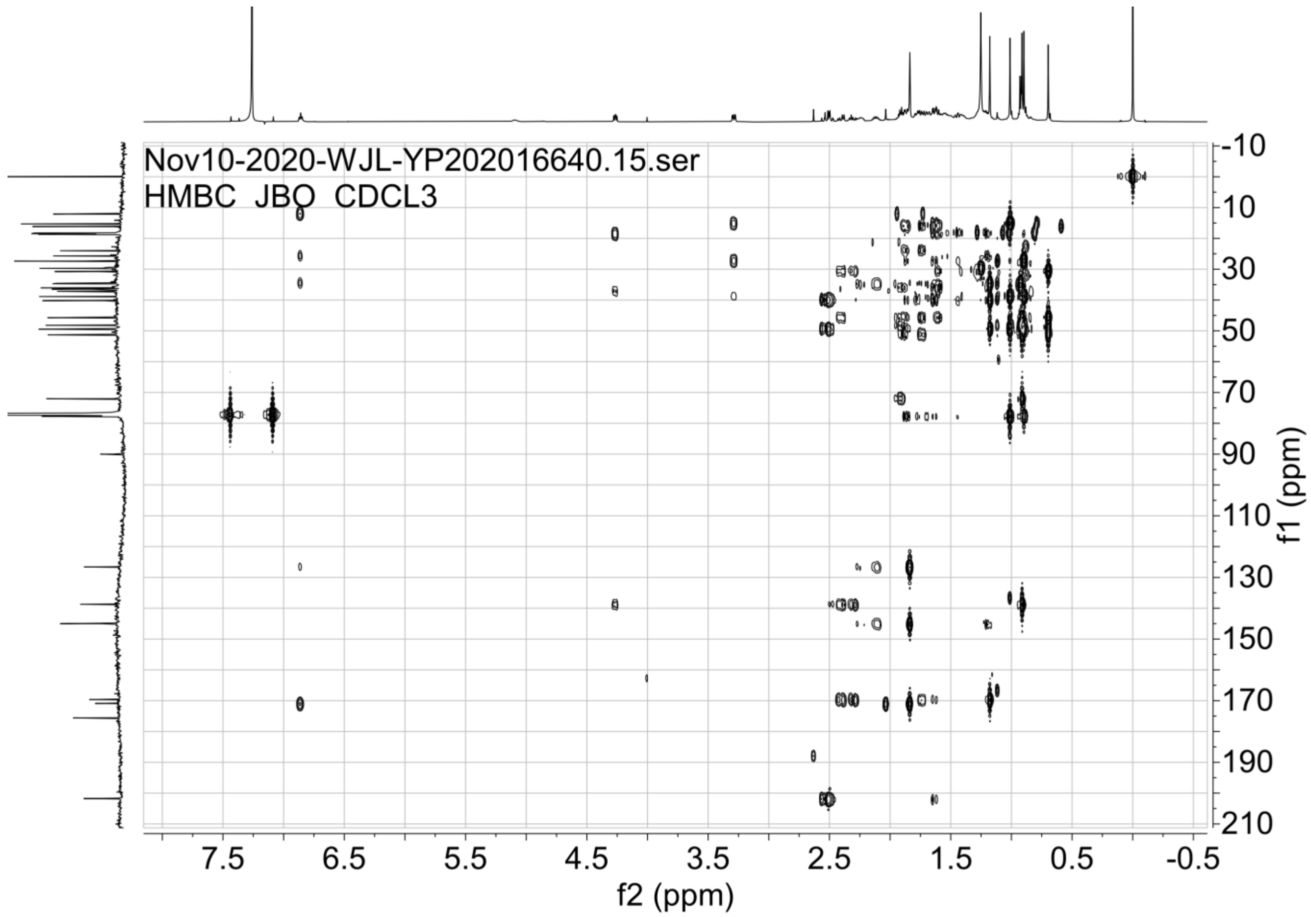

Supplementary Figure 45. HMBC spectrum of 7.

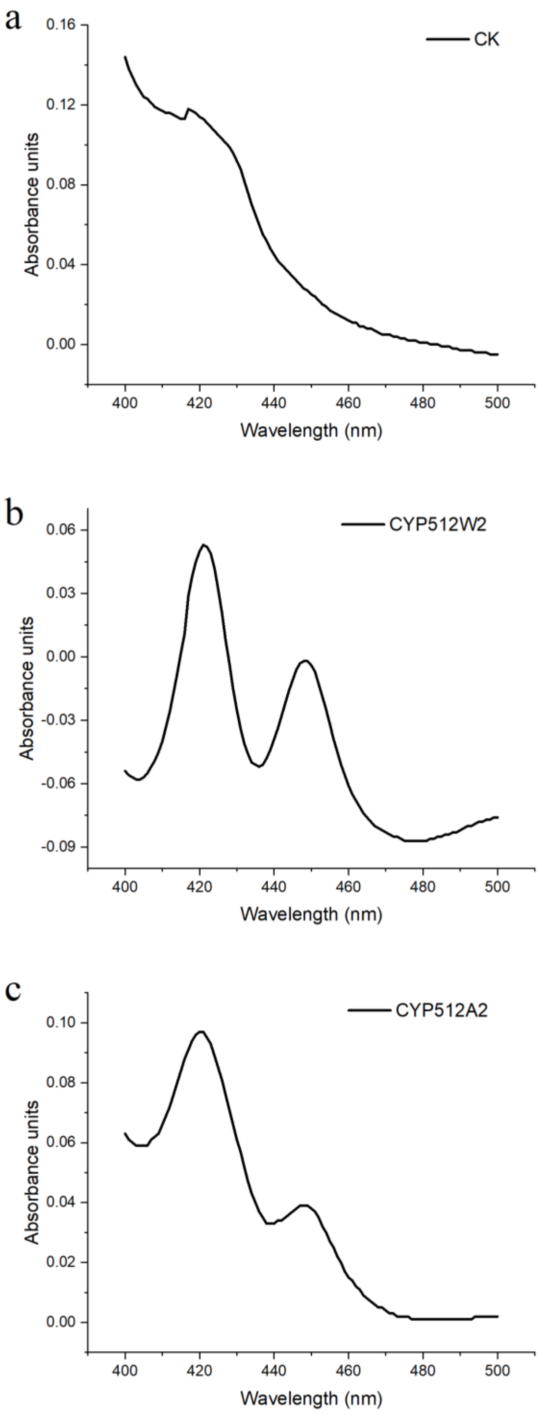

Supplementary Figure 46. P450 spectral analysis using yeast microsomes. a CK, the control strain CK-r-iGLCPR-r; **b** CYP512W2, strain CYP512W2-r-iGLCPR-r; **c** CYP512A2, strain CYP512A2-r-iGLCPR-r. For CK, CYP512W2, and CYP512A2, they were cultured in fermentation medium supplemented with 0 mg/L, 800 mg/L, and 300 mg/L of G418 for 60 h to prepare microsomes, respectively. Three mL of microsomes from 200 mL fermentation broth were obtained for each strain. The P450 spectral analysis was performed by using UV-VIS spectrophotometer (UV-2600, Shimadzu, Japan) and UVProbe (v 2.43)[1]. Significant CO-shift from 420 nm to 450 nm was detected for the CYP512W2 or CYP512A2 containing microsome (Supplementary Figure 46b and c). While for the control strain CK-r-iGLCPR-r derived microsome, we did not observe the CO-shift from 420 nm to 450 nm (Supplementary Figure 46a). Source data are provided as a Source Data file.

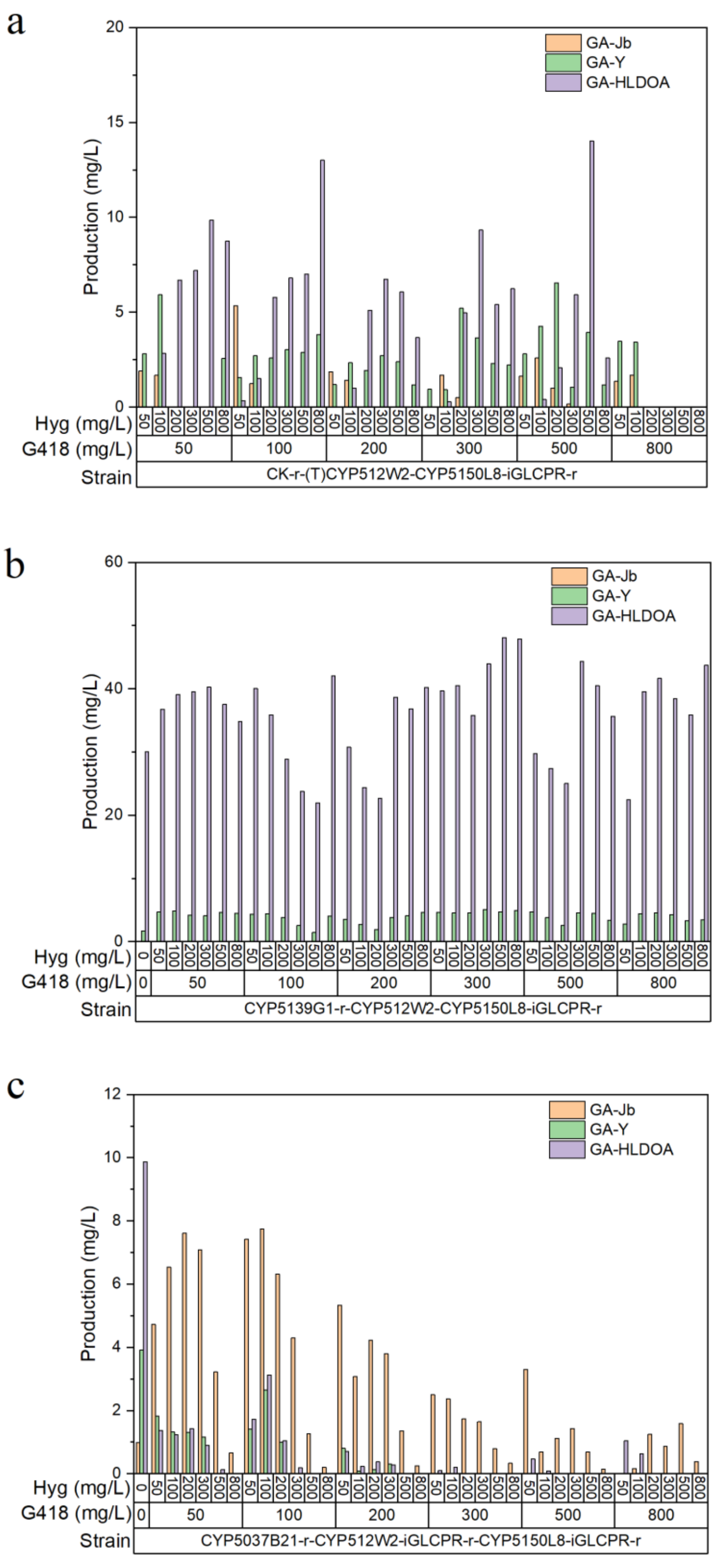

Supplementary Figure 47. Production of GA-Jb, GA-Y, and GA-HLDOA after 120 h fermentation of three strains. a CK-r-(T)CYP512W2-CYP5150L8-iGLCPR-r, **b** CYP5139G1-r-CYP512W2-CYP5150L8-iGLCPR-r, and **c** CYP5037B21-r-CYP512W2-iGLCPR-r-CYP5150L8-iGLCPR-r, under different concentrations G418 and hygromycin. All data represent the value of one sample. Source data are provided as a Source Data file.

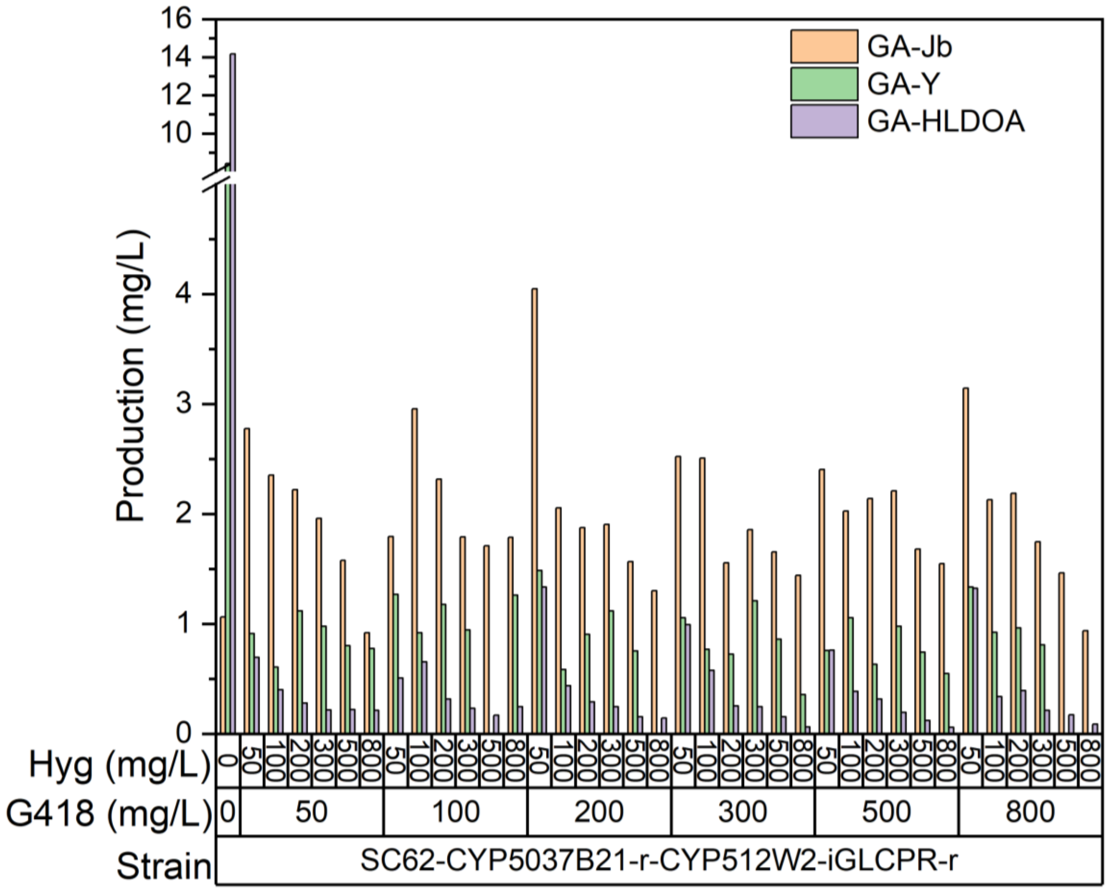

Supplementary Figure 48. Production of GA-Jb, GA-Y, and GA-HLDOA after 120 h fermentation of strain SC62-CYP5037B21-r-CYP512W2-iGLCPR-r, under different concentrations of G418 and hygromycin. All data represent the value of one sample. Source data are provided as a Source Data file.

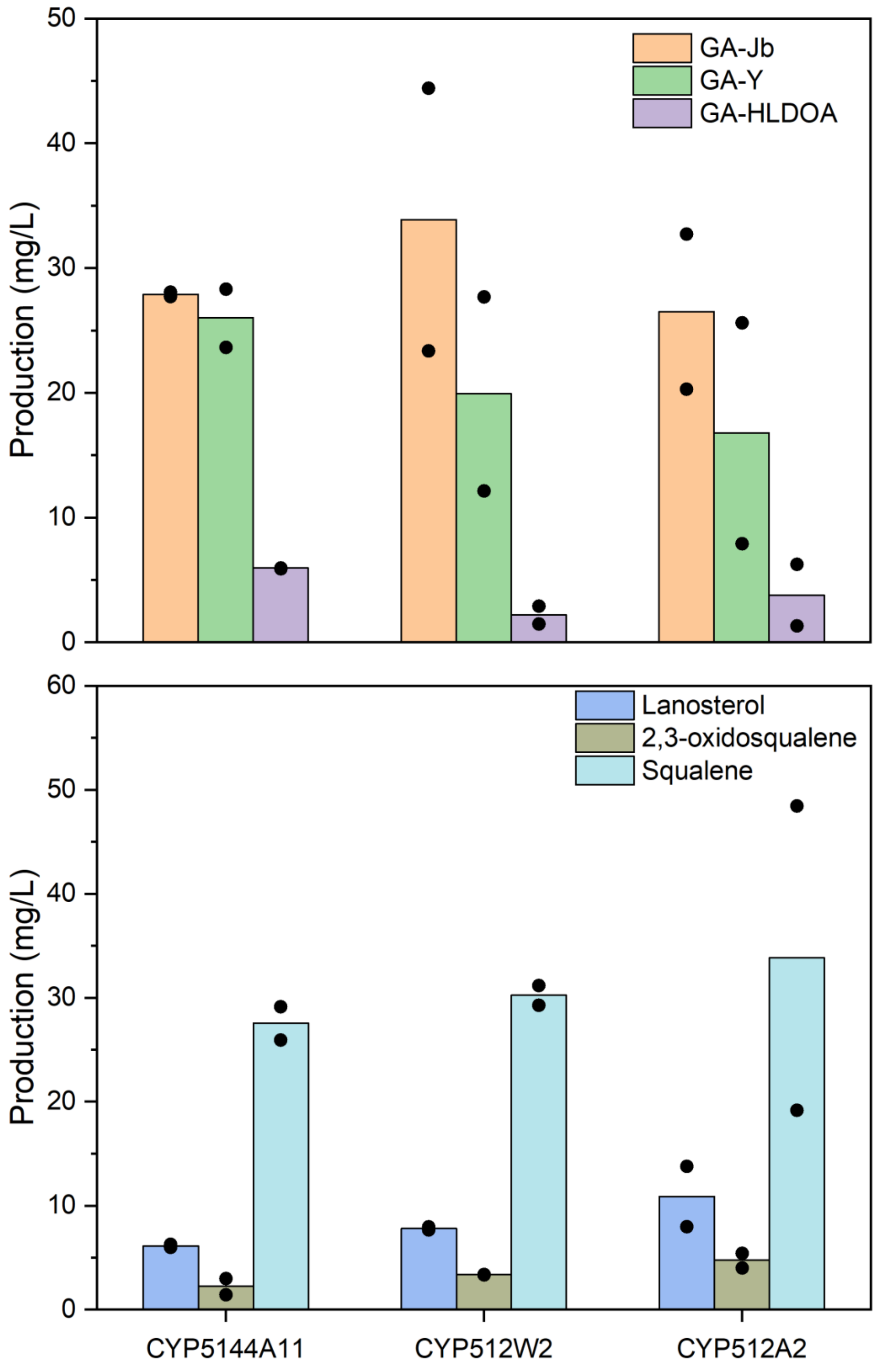

Supplementary Figure 49. Production of GA-Jb, GA-Y, GA-HLDOA, lanosterol, 2,3-oxidosqualene, and squalene after 120 h fermentation of strains SC62-CYP5144A11-r-CYP512W2-r, SC62-CYP512W2-r-CYP512W2-r, and SC62-CYP512A2-r-CYP512W2-r. CYP5144A11, strain SC62-CYP5144A11-r-CYP512W2-r; CYP512W2, strain SC62-CYP512W2-r-CYP512W2-r; CYP512A2, strain SC62-CYP512A2-r-CYP512W2-r. All data represent the mean of two biologically independent samples (solid circles). Source data are provided as a Source Data file.

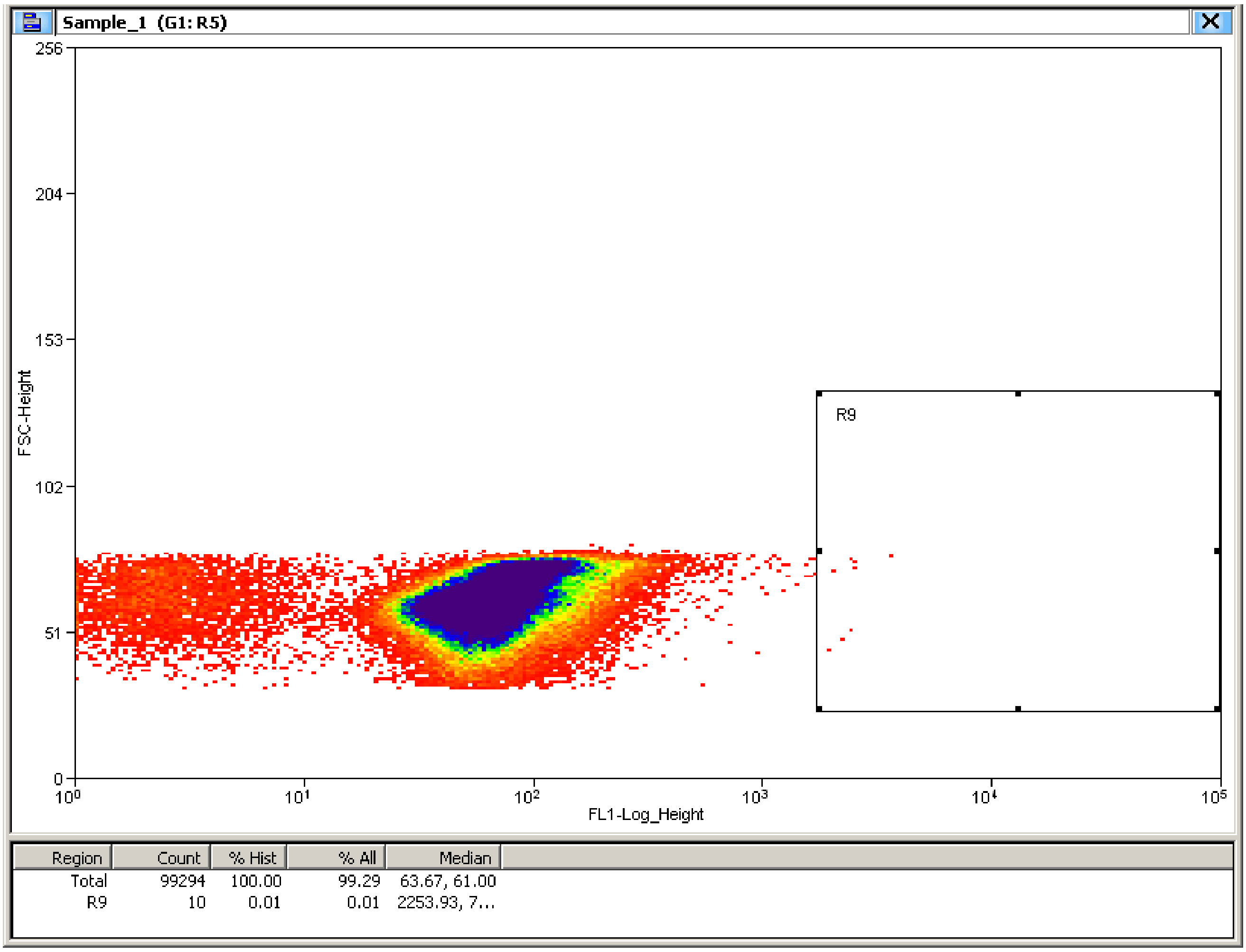

Supplementary Figure 50. Gating strategy for identifying high eGFP-expressing yeast cells. Yeast cell gating strategy followed: FSC (voltage 180 V), SSC (voltage 400 V), and the threshold was set as 3% (triggered on FSC channel). All captured events were used for fluorescence analysis. GFP fluorescence was analyzed on FL1 channel (voltage 400 V, excitation at 488 nm, emission fluorescence at 529 ± 14 nm). Cells with the top 0.01% fluorescence signal were collected.

Supplementary Table 1. Delta ppm for the detected *m/z* values.

**Compounds**	**Formula**	**Characteristic molecular fragment ions**	**Caculated *m/z***	**Detected *m/z***	**Delta ppm**
Lanosterol	C30H50O	$[M\text{-}H_2O+H]^+$	409.382878	409.38356	1.67
GA-HLDOA	C30H48O3	$[M\text{-}H_2O+H]^+$	439.357057	439.35777	1.62
**1**	C30H50O2	$[M\text{-}H_2O+H]^+$	425.377793	425.37796	0.39
**2**	C30H48O4	$[M\text{-}2H_2O+H]^+$	437.341407	437.34027	2.60
**3**	C30H46O3	$[M\text{-}H_2O+H]^+$	437.341407	437.34122	0.43
**4**	C30H46O4	$[M\text{-}H_2O+H]^+$	453.336322	453.33709	1.69
**5**	C30H48O4	$[M\text{-}2H_2O+H]^+$	437.341407	437.34111	0.68
**6**	C30H48O5	$[M\text{-}2H_2O+H]^+$	453.336322	453.33706	1.63
**7**	C30H46O5	$[M+H]^+$	487.341801	487.34079	2.07

Supplementary Table 2. Production of GA-Y and GA-Jb in previous reports.

Structures	Compounds	Description	Mushroom species	Sources	Content or titer	Production efficiency [a]	Ref.
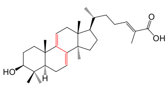	Ganoderic acid Y	(24E)-3-ol-5α-lanosta-7,9 (11),24-trien-26-oic acid	*G. lucidum*	200 g of dried mushroom fruiting bodies	300 mg/kg-DW	3.33 mg/kg-DW/d	2
			G. lucidum	10 kg of dried fruiting bodies	0.9 mg/kg-DW	0.01 mg/kg-DW/d	3
			G. resinaceum	25 kg of purchased fruiting bodies	2.32 mg/kg-DW	2.58×10^{-2} mg/kg-DW/d	4
			G. hainanense	2.5 kg of dry fruiting bodies	2.8 mg/kg-DW	3.11×10^{-2} mg/kg-DW/d	5
			G. concinna Ryv. Nov. sp	170 g of dried fruiting bodies	23.53 mg/kg-DW	0.26 mg/kg-DW/d	6
			G. leucocontextum	3 kg of air-dried fruiting bodies	16.7 mg/kg-DW	0.19 mg/kg-DW/d	7
			G. resinaceum Boud. (Ganodermataceae)	48 kg of dried fruiting bodies	4.96 mg/kg-DW	5.51×10^{-2} mg/kg-DW/d	8
			G. lucidum	2 kg of air-dried fruiting bodies	1.5 mg/kg-DW	1.67×10^{-2} mg/kg-DW/d	9
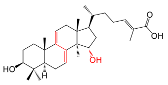	Ganodermic acid Jb	lanosta-7,9(11),24-trien-3β, 15α-dihydroxy-26-oic-acid, Ganoderic acid Jb, GAJb	*G. resinaceum* Boud. (Ganodermataceae)	48 kg of dried fruiting bodies	0.84 mg/kg-DW	9.33×10^{-3} mg/kg-DW/d	8
			G. lucidum (strain TP-1)	Mycelia harvested from 9 L liquid culture	0.22 mg/L	7.41×10^{-3} mg/L/d	10
			G. calidophilum	2.5 kg of dry fruiting bodies	8.92 mg/kg-DW	9.91×10^{-2} mg/kg-DW/d	11
			G. cochlear	68 kg of fruiting bodies	0.18 mg/kg-DW	0.002 mg/kg-DW/d	12

[a] Generally, it takes about 90 days for *Ganoderma* to complete a cycle of sexual generation under artificial cultivation conditions[13]. While fermentation of yeast needs about 6 days. The production efficiency is defined as the average daily content of products (mg/kg-DW/d) for fruiting body and yeast, or the average daily titer of products (mg/L/d) for fermentation-derived mycelia and yeast.

In our work, after 144 h fermentation, strain CYP512W2-r-CYP5150L8-iGLCPR-r produced 51.30 mg/L of (**3**) GA-Y and 56.44 mg/L of (**4**) GA-Jb, with OD_{600} at 30.35 ± 0.3446. According to the conversion method as published previously[14], strain CYP512W2-r-CYP5150L8-iGLCPR-r produced about 5.45 g/kg-DW of (**3**) GA-Y and 6.00 g/kg-DW of (**4**) GA-Jb. The production efficiencies of strain CYP512W2-r-CYP5150L8-iGLCPR-r for (**3**) GA-Y and (**4**) GA-Jb were 908.33 mg/kg-DW/d and 1000 mg/kg-DW/d, respectively.

Supplementary Note 1. A series of efforts to engineer a 3 and 4 producing yeast for subsequent screening.

We first constructed strain CK-r-(T)CYP512W2-CYP5150L8-iGLCPR-r, in which promoter Tdh3p and terminator Tef1t were adopted for CYP512W2 expression. After fermentation using different concentrations of G418 and hygromycin, the strain was able to produce 5.35 mg/L of **4** in the presence of 100 mg/L of G418 and 50 mg/L of hygromycin, and 6.55 mg/L of **3** in the presence of 500 mg/L of G418 and 200 mg/L of hygromycin after 120 h fermentation (Supplementary Figure 47a). However, current **4** and **3** production levels are not enough to facilitate an easy HPLC analysis in downstream screening. Next, we utilized the former used HXT7p instead of Tdh3p for driving the expression of CYP512W2. The resultant strain CYP5139G1-r-CYP512W2-CYP5150L8-iGLCPR-r, which harbored a randomly selected CYP expression plasmid pRS426-CYP5139G1-G418r, was able to produce 5.08 mg/L of **3** in the presence of 300 mg/L of G418 and hygromycin, but almost no **4** in all the tested fermentation conditions (Supplementary Figure 47b). Then, we considered to overexpress CYP512W2 and CYP5150L8 on pRS425 and pRS424 derived plasmids, respectively. Plasmid pRS424-HXT7p-CYP512W2-FBA1t-iGLCPR-Hygr was according constructed for CYP512W2 expression. The resultant reconstructed strain CYP5037B21-r-CYP512W2-iGLCPR-r-CYP5150L8-iGLCPR-r, which harbored a randomly picked CYP expression plasmid pRS426-CYP5037B21-G418r, was able to produce 7.75 mg/L of **4** and 2.66 mg/L of **3** in the presence of 100 mg/L of G418 and hygromycin (Supplementary Figure 47c). However, when other randomly selected CYP expression plasmids were replaced with pRS426-CYP5037B21-G418r, production of **4** and **3** from the corresponding strains were quite unstable, and sometimes even undetectable. The same promoter and terminator used for different gene expression cassettes on plasmids may cause plasmid instability due to the inherent high efficiency of homologous recombination in *S. cerevisiae*.

Thus, we considered to integrate the expression cassettes of CYP5150L8 and iGLCPR into yeast chromosome to obtain a strain capable of producing GA-HLDOA, and then use the tunable plasmid expression strategy for expressing CYP512W2 to produce **3** and **4**. To achieve multi-copy integration of CYP5150L8 and iGLCPR, retrotransposon Ty long terminal repeat (delta) sites were first selected for CRISPR-Cas9 assisted multi-copy integration[15]. However, we failed to obtain positive clone. Since gene expression cassettes P_{PGK1}-*tHMG1*-T_{ADH1}-

P_{TEF1}-*LYS2*-T_{CYC1} has already been integrated into the delta site of *S. cerevisiae* YL-T3[16], it may hinder the integration of CYP5150L8 and iGLCPR to similar delta sites with high sequence homology. Then, we switched to select the multi-copy rDNA loci for CRISPR-Cas9 assisted integration. However, we only obtain less than 30 colonies, which significantly reduced the possibility to obtain the desired phenotype.

Supplementary Note 2. Efforts to verify whether additional expression of iGLCPR was required for production of 3 and 4 in strain SC62.

To achieve stable production of **3** and **4**, we set to express CYP512W2 in strain SC62. Although iGLCPR expression cassette had been integrated into the chromosome of SC62, we wondered whether additional expression of iGLCPR was required for supporting the enzymatic activity of CYPs. Thus, we constructed six yeast strains, including SC62-CYP512W2-iGLCPR-r, SC62-CYP512W2-r, SC62-CYP5037B21-r-CYP512W2-iGLCPR-r, SC62-CK-r-CYP512W2-iGLCPR-r, SC62-CYP5037B21-r-CYP512W2-r, and SC62-CK-r-CYP512W2-r (Supplementary Data 4). We first conducted fermentation of strain SC62-CYP5037B21-r-CYP512W2-iGLCPR-r with different concentrations of G418 and hygromycin ranging from 0 to 800 mg/L. But the production of **4** and **3** were no more than 4.5 mg/L (Supplementary Figure 48). Then, fermentation of these six engineered strains were performed in 24-well plate containing different concentrations of G418 and hygromycin. As a result, strain SC62-CYP512W2-iGLCPR-r and SC62-CYP512W2-r produced no more than 1 mg/L **4** and 3-8 mg/L **3**, without the presence of hygromycin in the fermentation media. When hygromycin was added, for unknown reasons, **4**, **3** and GA-HLDOA were almost undetectable from these strains. In contrast, strains SC62-CK-r-CYP512W2-iGLCPR-r, SC62-CK-r-CYP512W2-r, SC62-CYP5037B21-r-CYP512W2-iGLCPR-r and SC62-CYP5037B21-r-CYP512W2-r had higher **4** and **3** production, and lower production of GA-HLDOA in the presence of 100-200 mg/L of G418 and hygromycin than those without antibiotics. In another aspect, strain SC62-CK-r-CYP512W2-iGLCPR-r produced only slightly higher average amount of **4** and **3** than those from strain SC62-CK-r-CYP512W2-r at their optimal fermentation conditions (Fig. 5d). Moreover, strain SC62-CYP5037B21-r-CYP512W2-r could generate even more **4** and **3** than those from strain SC62-CYP5037B21-r-CYP512W2-iGLCPR-r at their optimal fermentation conditions (Fig. 5d). Taken together, these results suggested that additional expression of iGLCPR in SC62 was not necessary.

Supplementary references

1. Guengerich FP, Martin MV, Sohl CD, Cheng Q. Measurement of cytochrome P450 and NADPH-cytochrome P450 reductase. *Nat Protoc* **4**, 1245-1251 (2009).

2. Hajjaj H, Mace C, Roberts M, Niederberger P, Fay LB. Effect of 26-oxygenosterols from *Ganoderma lucidum* and their activity as cholesterol synthesis inhibitors. *Appl Environ Microbiol* **71**, 3653-3658 (2005).

3. Lee I, Ahn B, Choi J, Hattori M, Min B, Bae K. Selective cholinesterase inhibition by lanostane triterpenes from fruiting bodies of *Ganoderma lucidum*. *Bioorganic Med Chem Lett* **21**, 6603-6607 (2011).

4. Peng XR, Liu JQ, Han ZH, Yuan XX, Luo HR, Qiu MH. Protective effects of triterpenoids from *Ganoderma resinaceum* on H_2O_2-induced toxicity in HepG2 cells. *Food Chem* **141**, 920-926 (2013).

5. Ma QY, Luo Y, Huang SZ, Guo ZK, Dai HF, Zhao YX. Lanostane triterpenoids with cytotoxic activities from the fruiting bodies of *Ganoderma hainanense*. *J Asian Nat Prod Res* **15**, 1214-1219 (2013).

6. Gonzalez AG, *et al.* New lanostanoids from the fungus *Ganoderma concinna*. *J Nat Prod* **65**, 417-421 (2002).

7. Wang K, *et al.* Lanostane triterpenes from the Tibetan medicinal mushroom *Ganoderma leucocontextum* and their inhibitory effects on HMG-CoA reductase and α-glucosidase. *J Nat Prod* **78**, 1977-1989 (2015).

8. Chen XQ, Zhao J, Chen LX, Wang SF, Wang Y, Li SP. Lanostane triterpenes from the mushroom *Ganoderma resinaceum* and their inhibitory activities against alpha-glucosidase. *Phytochemistry* **149**, 103-115 (2018).

9. Cheng CR, *et al.* Cytotoxic triterpenoids from *Ganoderma lucidum*. *Phytochemistry* **71**, 1579-1585 (2010).

10. Shiao MS, Lin LJ, Yeh SF. Triterpenes in *Ganoderma lucidum*. *Phytochemistry* **27**, 873-875 (1988).

11. Huang SZ, *et al.* Lanostane-type triterpenoids from the fruiting body of *Ganoderma calidophilum*. *Phytochemistry* **143**, 104-110 (2017).

12. Peng XR, Wang X, Zhou L, Hou B, Zuo ZL, Qiu MH. Ganocochlearic acid A, a rearranged hexanorlanostane triterpenoid, and cytotoxic triterpenoids from the fruiting bodies of *Ganoderma cochlear*. *Rsc Advances* **5**, 95212-95222 (2015).

13. Sun C, *et al.* *Ganoderma lucidum*: an emerging medicinal model fungus for study of the biosynthesis of natural medicines. *Scientia Sinica(Vitae)* **43**, 447-456 (2013).

14. Katahira S, Mizuike A, Fukuda H, Kondo A. Ethanol fermentation from lignocellulosic hydrolysate by a recombinant xylose- and cellooligosaccharide-assimilating yeast strain. *Appl Microbiol Biotechnol* **72**, 1136-1143 (2006).

15. Shi S, Liang Y, Zhang MM, Ang EL, Zhao H. A highly efficient single-step, markerless strategy for multi-copy chromosomal integration of large biochemical pathways in *Saccharomyces cerevisiae*. *Metab Eng* **33**, 19-27 (2016).

16. Dai Z, *et al.* Producing aglycons of ginsenosides in bakers' yeast. *Sci Rep* **4**, 3698 (2014).
