# Supplementary material for: Biosynthesis of mushroom-derived type II ganoderic acids by engineered yeast
Source: Nat Commun. 2022 Dec 14;13:7740. doi: 10.1038/s41467-022-35500-1 (PMC9748899; doi:10.1038/s41467-022-35500-1)
Supplement: Supplementary file 2 — Description of Additional Supplementary Files [file 41467_2022_35500_MOESM2_ESM.pdf]

Description of Additional Supplementary Files

File Name: Supplementary Data 1

Description: CYPs used in this study.

File Name: Supplementary Data 2

Description: RNA sequencing data of CYPs in G. lucidum.

File Name: Supplementary Data 3

Description: Primers used in this study for CYPs amplification.

File Name: Supplementary Data 4

Description: Scheme for plasmids and strains construction.

File Name: Supplementary Data 5

Description: 13C-NMR and 1H-NMR data of compounds.
