# Supplementary material for: Biosynthesis of mushroom-derived type II ganoderic acids by engineered yeast
Source: Nat Commun. 2022 Dec 14;13:7740. doi: 10.1038/s41467-022-35500-1 (PMC9748899; doi:10.1038/s41467-022-35500-1)
Supplement: Supplementary file 8 — Reporting Summary [file 41467_2022_35500_MOESM8_ESM.pdf]

nature portfolio

Corresponding author(s): Han Xiao

Last updated by author(s): Dec 2, 2022

Reporting Summary

Nature Portfolio wishes to improve the reproducibility of the work that we publish. This form provides structure for consistency and transparency in reporting. For further information on Nature Portfolio policies, see our Editorial Policies and the Editorial Policy Checklist.

Statistics

For all statistical analyses, confirm that the following items are present in the figure legend, table legend, main text, or Methods section.

n/a	Confirmed	
☐	☒	The exact sample size (*n*) for each experimental group/condition, given as a discrete number and unit of measurement
☐	☒	A statement on whether measurements were taken from distinct samples or whether the same sample was measured repeatedly
☐	☒	The statistical test(s) used AND whether they are one- or two-sided<br>*Only common tests should be described solely by name; describe more complex techniques in the Methods section.*
☒	☐	A description of all covariates tested
☒	☐	A description of any assumptions or corrections, such as tests of normality and adjustment for multiple comparisons
☐	☒	A full description of the statistical parameters including central tendency (e.g. means) or other basic estimates (e.g. regression coefficient) AND variation (e.g. standard deviation) or associated estimates of uncertainty (e.g. confidence intervals)
☐	☒	For null hypothesis testing, the test statistic (e.g. *F*, *t*, *r*) with confidence intervals, effect sizes, degrees of freedom and *P* value noted<br>*Give P values as exact values whenever suitable.*
☒	☐	For Bayesian analysis, information on the choice of priors and Markov chain Monte Carlo settings
☒	☐	For hierarchical and complex designs, identification of the appropriate level for tests and full reporting of outcomes
☒	☐	Estimates of effect sizes (e.g. Cohen's *d*, Pearson's *r*), indicating how they were calculated

Our web collection on statistics for biologists contains articles on many of the points above.

Software and code

Policy information about availability of computer code

Data collection

1. OpenLAB CDS ChemStation Edition for Agilent 1260 Infinity II HPLC system (Agilent, Waldbronn, Germany) was used for collection of HPLC data.
2. UNIFI (v 1.9.4.053, Waters, Corp.) for Q-TOF MS (Waters, Wilmslow, UK) was used for collection of LC-MS data.
3. TopSpin 3.2 and TopSpin3.2.5 for Avance III 600 MHz Nuclear Magnetic Resonance instrument (Bruker, Karlsruhe, Germany) were used for collection of NMR data.
4. GFP fluorescence was collected by flow cytometry (FACS, Beckman Coulter MoFlo XDP) with the following parameters: excitation fluorescence at 488 nm, detection fluorescence at 529 ± 14 nm.
5. UV-VIS spectrophotometer (UV-2600, Shimadzu, Japan) was used to perform P450 spectral analysis.

Data analysis

1. All P values were generated from two-tailed t-tests by using OriginPro (v 9.8.0.200, OriginLab Corporation) software.
2. LC-MS data were analyzed by using UNIFI (v 1.9.4.053, Waters, Corp.).
3. NMR data were analyzed by using MestReNova (v 14.0.0)
4. FACS data were analyzed by using the MoFlo XDP Summit Software(v 5.2).
5. Genome assembly was performed using SMRT Link v5.0.1 (Pacific Biosciences, Menlo Park, CA, USA).
6. Illumina reads were used to reduce the degree of heterozygosity using Purge Haplotigs software (https://bitbucket.org/mroachawri/purge_haplotigs/src/master/)
7. RagTag software (https://github.com/malonge/RagTag) was used for reference-guided scaffolding.
8. Illumina reads were used for error correction using Pilon software (https://github.com/broadinstitute/pilon).
9. RNA sequencing and protein data from G. lucidum were subjected to accurate gene structure annotation by Braker2 (https://anaconda.org/bioconda/braker2).
10. The clean reads of transcriptome data were aligned to the genome assembly using Hisat2 (https://daehwankimlab.github.io/hisat2/

download/).
11. Stringtie2 (http://ccb.jhu.edu/software/stringtie/index.shtml) was used for fragments per kilobase of sequence per million mapped reads (FPKM) calculations.
12. The FPKM value of each transcript was calculated based on the length of the transcript and number of reads mapped to the transcript.
13. UVProbe 2.43 was used for P450 spectral analysis.

For manuscripts utilizing custom algorithms or software that are central to the research but not yet described in published literature, software must be made available to editors and reviewers. We strongly encourage code deposition in a community repository (e.g. GitHub). See the Nature Portfolio guidelines for submitting code & software for further information.

Data

Policy information about availability of data

All manuscripts must include a data availability statement. This statement should provide the following information, where applicable:

- Accession codes, unique identifiers, or web links for publicly available datasets
- A description of any restrictions on data availability
- For clinical datasets or third party data, please ensure that the statement adheres to our policy

The data supporting the findings of this work are available within the paper and the Supplementary Information files. A reporting summary for this article is available as a Supplementary Information file. Source data are provided with this paper. The genome and transcriptome sequencing data can be found in the website (https://www.ncbi.nlm.nih.gov/bioproject/PRJNA796760). The annotation data are available in GitHub (https://github.com/TIBseqlab/Galuci_5_616).

Human research participants

Policy information about studies involving human research participants and Sex and Gender in Research.

Reporting on sex and gender	There were no human research participants in our research. So no sex or gender data were collected and analyzed.
Population characteristics	See above.
Recruitment	See above.
Ethics oversight	See above.

Note that full information on the approval of the study protocol must also be provided in the manuscript.

Field-specific reporting

Please select the one below that is the best fit for your research. If you are not sure, read the appropriate sections before making your selection.

☒ Life sciences ☐ Behavioural & social sciences ☐ Ecological, evolutionary & environmental sciences

For a reference copy of the document with all sections, see nature.com/documents/nr-reporting-summary-flat.pdf

Life sciences study design

All studies must disclose on these points even when the disclosure is negative.

Sample size	For one CYP expressing plasmid transformed into the yeast strain, when conducting functional CYPs screening, two transformants were randomly picked for fermentation assay. So for screening 158 CYP expression plasmids in one round, 318 yeast transformants (including two randomly picked transformants of control strain) were picked for assay. When performing functional identification of one gene, in general, it is sufficient to select a single clone for fermentation assay. As only qualitative analysis of the fermentation products is needed, parallel settings were required. Here in this work, we chose two clones for fermentation assay, just to avoid the false positive clones with a remote probability.<br>When conduction shake-flask fermentation to verify production of GA-Jb and GA-Y, four transformants were randomly picked. In general, no less than three repeats were necessary to perform statistical analysis.
Data exclusions	No data were excluded from the analyses.
Replication	For all the screened out yeast strains, the replication of their fermentation assay were successful. All replicates performed in this study were biological replicates.
Randomization	To investigate whether new ganoderic acids or relevant compounds were generated in the engineered strains, the CYP(s) containing yeasts were grouped into the experimental group. In contrast, yeast strain harboring the void plasmid served as the control group. Therefore, the allocation was not relevant to this study.
Blinding	To investigate whether new ganoderic acids or relevant compounds were generated in the engineered strains, the CYP(s) containing yeasts

Blinding	were grouped into the experimental group. In contrast, yeast strain harboring the void plasmid served as the control group. Therefore, the blinding was not relevant to this study.

Reporting for specific materials, systems and methods

We require information from authors about some types of materials, experimental systems and methods used in many studies. Here, indicate whether each material, system or method listed is relevant to your study. If you are not sure if a list item applies to your research, read the appropriate section before selecting a response.

Materials & experimental systems

n/a	Involved in the study
☒	☐ Antibodies
☐	☒ Eukaryotic cell lines
☒	☐ Palaeontology and archaeology
☒	☐ Animals and other organisms
☒	☐ Clinical data
☒	☐ Dual use research of concern

Methods

n/a	Involved in the study
☒	☐ ChIP-seq
☐	☒ Flow cytometry
☒	☐ MRI-based neuroimaging

Eukaryotic cell lines

Policy information about cell lines and Sex and Gender in Research

Cell line source(s)	The Saccharomyces cerevisiae strain YL-T3 were provided by Dr. Xueli Zhang (Tianjin Institute of Industrial Biotechnology, Chinese Academy of Sciences, China) as described in our previous work (Biotechnol Bioeng, 2018, 115 (7): 1842-1854).
Authentication	The origin strain YL-T3 used in this study was not authenticated by the authors of this study.
Mycoplasma contamination	The yeast cell lines were not tested for mycoplasma contamination.
Commonly misidentified lines (See ICLAC register)	Yeast strain SC62 could be cultured in YPD medium or synthetic complete drop-out medium lacking histidine and tryptophan.

Flow Cytometry

Plots

Confirm that:

☒ The axis labels state the marker and fluorochrome used (e.g. CD4-FITC).

☒ The axis scales are clearly visible. Include numbers along axes only for bottom left plot of group (a 'group' is an analysis of identical markers).

☒ All plots are contour plots with outliers or pseudocolor plots.

☒ A numerical value for number of cells or percentage (with statistics) is provided.

Methodology

Sample preparation	The CYP5150L8 and iGLCPR expression cassettes were integrated at rDNA loci of yeast strain YL-T3 by simple homologous recombination. The eGFP expression cassette was incorporated into the donor, allowing fluorescence activated cell sorting (FACS) for screening of successfully integrated transformants. The donor was transformed into YL-T3. SC-HT solid plate was used for transformants selection. After incubation at 30 °C for 2 days. Over 5,000 transformants were obtained, mixed, re-suspended by 3 mL of sterilized water, and inoculated with a volume ratio of 1% into 4 mL of SC-HT liquid medium in a 16 mL glass tube and cultivated at 30 °C and 220 rpm to an OD600 of 1.5-2.5. Then the culture was inoculated at an initial OD600 of 0.2 to an OD600 of 1.5-2.5. Cells were harvested by centrifugation (10000 g, 30 s), washed once using PBS (pH 7.4), and re-suspended in PBS (pH 7.4) to an OD600 of 0.1-0.2. The cells were dispersed by ultrasound for 2 min. Then, 10 mL of the cell suspension was used for FACS to isolate GFP-positive cells.
Instrument	MoFlo XDP high-speed sorter, Beckman Coulter, Fullerton, CA, USA
Software	MoFlo XDP Summit Software (v 5.2)
Cell population abundance	After FACS, 960 cells (accounting for 0.01% of all the analyzed yeast cells) were isolated and individually inoculated for further assay.
Gating strategy	Yeast cell gating strategy followed: FSC (voltage 180 V), SSC (voltage 400 V), and the threshold was set as 3% (triggered on FSC channel). All captured events were used for fluorescence analysis. GFP fluorescence was analyzed on FL1 channel

(voltage 400 V, excitation at 488 nm, emission fluorescence at 529 ± 14 nm). Cells with the top 0.01% fluorescence signal were collected.

☒ Tick this box to confirm that a figure exemplifying the gating strategy is provided in the Supplementary Information.
